# Techno-economic evaluation of microalgae high-density liquid fuel production at 12 international locations

**DOI:** 10.1186/s13068-021-01972-4

**Published:** 2021-06-07

**Authors:** John Roles, Jennifer Yarnold, Karen Hussey, Ben Hankamer

**Affiliations:** 1 Institute for Molecular Bioscience, The University of Queensland, 306 Carmody Road, Brisbane, QLD 4072 Australia; 2 Centre for Policy Futures, Faculty of Humanities and Social Sciences, The University of Queensland, Brisbane, QLD 4072 Australia

**Keywords:** Renewable fuels, Algae-based fuel, Techno-economic analysis, Energy production, Fuel security

## Abstract

**Background:**

Microalgae-based high-density fuels offer an efficient and environmental pathway towards decarbonization of the transport sector and could be produced as part of a globally distributed network without competing with food systems for arable land. Variations in climatic and economic conditions significantly impact the economic feasibility and productivity of such fuel systems, requiring harmonized technoeconomic assessments to identify important conditions required for commercial scale up.

**Methods:**

Here, our previously validated *Techno-economic and Lifecycle Analysis* (TELCA) platform was extended to provide a direct performance comparison of microalgae diesel production at 12 international locations with variable climatic and economic settings. For each location, historical weather data, and jurisdiction-specific policy and economic inputs were used to simulate algal productivity, evaporation rates, harvest regime, CapEx and OpEx, interest and tax under location-specific operational parameters optimized for Minimum Diesel Selling Price (MDSP, US$ L^−1^). The economic feasibility, production capacity and CO_2-eq_ emissions of a defined 500 ha algae-based diesel production facility is reported for each.

**Results:**

Under a for-profit business model, 10 of the 12 locations achieved a minimum diesel selling price (MDSP) under US$ 1.85 L^−1^ / US$ 6.99 gal^−1^. At a fixed theoretical MDSP of US$ 2 L^−1^ (US$ 7.57 gal^−1^) these locations could achieve a profitable Internal Rate of Return (IRR) of 9.5–22.1%. Under a public utility model (0% profit, 0% tax) eight locations delivered cost-competitive renewable diesel at an MDSP of < US$ 1.24 L^−1^ (US$ 4.69 gal^−1^). The CO_2-eq_ emissions of microalgae diesel were about one-third of fossil-based diesel.

**Conclusions:**

The public utility approach could reduce the fuel price toward cost-competitiveness, providing a key step on the path to a profitable fully commercial renewable fuel industry by attracting the investment needed to advance technology and commercial biorefinery co-production options. Governments’ adoption of such an approach could accelerate decarbonization, improve fuel security, and help support a local COVID-19 economic recovery. This study highlights the benefits and limitations of different factors at each location (e.g., climate, labour costs, policy, C-credits) in terms of the development of the technology—providing insights on how governments, investors and industry can drive the technology forward.

**Graphic abstract:**

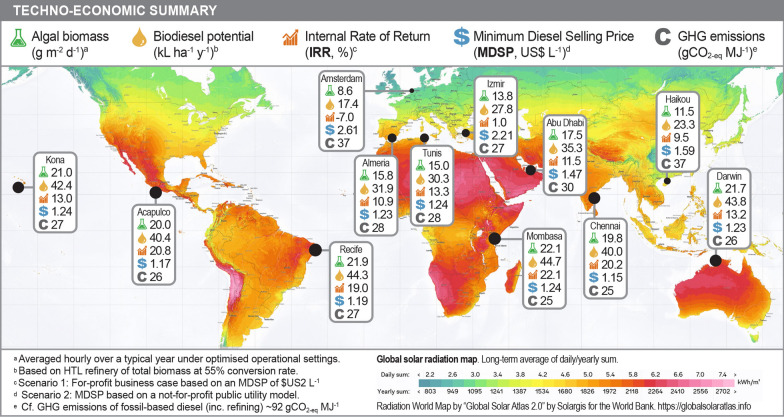

**Supplementary Information:**

The online version contains supplementary material available at 10.1186/s13068-021-01972-4.

## Introduction

In 2018, global energy consumption grew at twice the average rate recorded in 2010 [1], driven by a growing economy valued at US$ 136 trillion [2] and increased heating and cooling demands [1]. Despite global commitments on climate action, significant growth in renewables failed to keep pace with energy demand, resulting in a rise in greenhouse gas emissions (GHGs). Previously, the OECD called upon governments to develop enabling policy frameworks that will catalyze private sector investment to drive the large-scale transformation needed for a low carbon energy sector [3]. Substantial progress in renewable wind and solar PV technologies is driving a significant increase in renewable electricity supply and, coupled with battery technologies, is also transitioning the small vehicles market. However, high-density liquid fuels are critically underdeveloped and are expected to remain essential for the heavy transport, aviation, shipping, and logistics sectors for the foreseeable future, which combined, account for 12.7% of global energy demand [4]. As these fuels account for approximately 10% of global anthropogenic CO_2_ emissions [5], the development of low carbon alternative fuels is essential to meet international COP21 Paris CO_2_ emission reduction commitments and UN Sustainable Development Goals [6].

Advanced microalgae-based renewable fuel systems have significant potential to address these needs and to support a globally distributed and dispatchable fuel network to contribute to political, economic, social, environmental, fuel and climate security [7]. Current first-generation biofuel technologies, reliant on food crops, such as bioethanol from corn or sugar and biodiesel from soy or palm oil, compete with food production for arable land and fresh water and contribute to eutrophication [8, 9]. In contrast, microalgae systems can utilize saltwater and/or nutrient-rich wastewater and be deployed on non-arable land or in the oceans. These factors, coupled with high solar conversion efficiencies, can tap into the abundance of available solar energy (~ 3000 ZJ year^−1^ or ~ 5000 × global energy demand) to capture CO_2_, provide feedstocks for renewable fuel production, and expand global photosynthetic productivity. Ringsmuth et al. [10] estimated that supply of global diesel, aviation and shipping fuel needs could theoretically be provided by microalgae-based fuel production [4, 10] using only 0.18% of global surface area [10]—less than 10% of the area currently used by agriculture.

Advancing microalgae-based fuel technologies to a sustainable and commercial scale requires detailed and robust techno-economic and lifecycle analysis. This, in turn, is critical to attract an appropriate share of the renewable energy investment pool (cumulative US2.9 trillion since 2004) [11] that can advance the technology further. It can also support governments to define key areas of policy development, more quickly [12].

A number of reported models have evaluated the potential of algae-based renewable fuel systems [12–24]. Such studies have considered the effects of factors related to climate (e.g., solar radiation, temperature); operating conditions (e.g., nutrients, mixing regime, light regime, cell density); biology (growth, light tolerance, metabolic profile); or processes (e.g., harvest regime, fuel conversion method) on output variables categorized by: productivity (e.g., photosynthetic conversion efficiency, biomass yield, lipid yield or biofuel yield); economic feasibility (e.g., internal rate of return (IRR), minimum selling price (MSP); environmental performance (e.g., energy return on energy invested (EROEI), CO_2_ emissions per unit energy, life cycle analyses); scalability; or a combination thereof.

Naturally, the key determinants of economic feasibility are to produce the most fuel at the minimum cost. Selection of appropriate locations to establish microalgae-based biofuel production facilities is, therefore, critical due to the dual effects of climatic conditions on algae growth and production potential, and widely differing economic and policy settings between jurisdictions that effect the production cost.

Comparisons between locations, to date, have mostly assessed the productivity potential of microalgae systems as a function of climatic variables, particularly solar radiation [22, 24] and temperature [24, 25]. For example, Moody and co-authors (2014) integrated historical meteorological data with a growth model to evaluate lipid productivity of *Nannochloropsis* at 4388 global locations and reported the highest annual average lipid yields to be in the range of 24 and 27 m^3^ ha^−1^ year^−1^, in Australia, Brazil, Colombia, Egypt, Ethiopia, India, Kenya, and Saudi Arabia [26]. In contrast, techno-economic assessments (TEA) evaluate the economic feasibility and often combine process-based modelling related to reactor or facility designs and technologies with economic input values. Many TEAs are limited to one climatic zone or several climatic zones within one jurisdiction. For example, a study by Davis et al. [24] modelled the costs, resource requirements and emissions for production of five billion gallons of fuel at various locations across the US. Biomass peak productivities of up to 25–30 g m^−2^ day^−1^ were assumed to be achievable and fuel produced at a minimum diesel selling price (MDSP) of < US$ 1.82 L^−1^ (US$ 7 Gal^−1^) [24].

In general, wide variations between model assumptions and approaches has made it difficult to compare like with like*,* to identify the most suitable systems, processes, and locations for deployment at scale. A comprehensive review of algae-based biofuel models by Quinn and Davis [27] emphasized the importance of harmonized assessments to enable direct comparisons, and highlighted the need to consider the exact location of the production plant which has important impacts on productivity, CapEx, OpEx [28] as well as financial inputs. Our recent work confirmed these findings and further revealed the critical influence of policy settings which vary markedly across global jurisdictions [12]. The lack of harmonization in current assessments has resulted in large discrepancies between estimated algae-based renewable fuel costs that range from US$ 0.43 L^−1^ (US$ 1.64 gal^−1^) to over US$ 7.92 L^−1^(US$ 30.00 gal^−1^) [27].

This study builds on Roles et al. [12] to address this critical knowledge gap by benchmarking the economic feasibility of microalgae-based biodiesel production across 12 international locations to identify important conditions required for commercial scale up. The specific objectives of this study were to:

1. Simulate the operation of a microalgae high-density liquid fuel production facility benchmarked with the same key system and operational assumptions at 12 international locations.
2. Assess the production capacity across sites by accounting for variations in light- and temperature-dependent algal biomass production potential of each location.
3. Determine the lowest theoretical Minimum Diesel Selling Price (MDSP) based on the 12 locations analyzed, compare the range in MDSP variations across these sites and explore a process for the identification of promising locations for global microalgae fuel production.
4. Identify and prioritize the factors including financial drivers that created the largest differences in MDSP.

Our analysis accounts for critical location-dependent variables that affect production capacity, production cost and net emissions. It is based on extensive work on the development and validation of our integrated Techno-Economic and Life Cycle Assessment (TELCA) model of the microalgae liquid fuel production facility detailed in Roles et al. [12] (see also Additional file 1). This work demonstrated an economic, energy-efficient, and low CO_2_ emission pathway to deliver micro-algae-based high-density liquid fuels through a combination of technology, scale, policy and location-specific cost settings. The study highlighted the critical importance of factors other than technological advancements on the economic feasibility of fuel production—in particular, the role of policy settings. Here, our simulation is extended with location-specific inputs to provide a techno-economic evaluation of microalgae-based high-density liquid fuel production across a diverse range of locations and jurisdictions at a commercially optimized scale of 500 ha total pond area (see “Methods” and Additional file 1). Actual temporally and spatially resolved weather data including solar radiation, temperature, and humidity were used as inputs to enable dynamic modelling of biomass productivity and evaporation. Materials, labour costs, tax and interest rates were applied for each jurisdiction. The analysis provides a direct performance comparison of a well-defined microalgae renewable diesel production system [29] across 12 locations distributed throughout six continents, and covering a broad range of climatic (Graphical abstract, temperate to tropical) and economic conditions (Table 2). A base system was fixed for all locations, while process modelling was used to optimize a range of operational settings to improve the economics for each location including: strain selection, pond depth, culture density, harvesting regime and water sourcing**.**

Significantly, we identify important operational factors that can be improved for individual locations to increase productivity while driving down price and emissions; evaluate the impact of different economic and policy settings between jurisdictions and demonstrate the use of our TELCA platform to assist in model guided systems optimization to de-risk scale up and support business development.

## Methods

### Analytical framework

All techno-economic analyses are limited by the quality of the input data, the assumptions made, and the calculations conducted. Extensive work has previously been completed to validate the input data, the response of each process module, subprocesses and the whole process described by the 500 ha renewable high-density liquid fuel production facility [12]. Additional file 1 details the simulation used, and within it, Section 4 provides the model validation. Following internal data, module, subprocess and process validation, the TELCA model was next validated against a broad range of independent techno-economic and life-cycle analyses (Additional file 1: Figure S26). Of these, we consider the NREL model [13] (Additional file 1: Figure S26) to be the most comprehensive. Given the complexity of our TELCA model and that of the NREL model, and the fact that when set to the same production conditions they yielded a mean diesel selling Price within 1% of one another, we conclude that the NREL and TELCA models independently validate each other. This analysis not only confirms the robustness of TELCA but also of the NREL model. Finally, we conducted validation against an operational demonstration scale 0.4 ha microalgae production facility; the TELCA simulation of this facility identified the facilities CapEx to within 5% of the actual construction cost. Indeed, the TELCA evaluation delivered a calculated CapEx cost 5% above the actual construction costs suggesting that the assumptions were reasonably conservative (i.e., US$ 52.5 m^2^) at the 0.4 ha scale (i.e., US$ 525,000 ha^−1^).

**Fig. 1:**
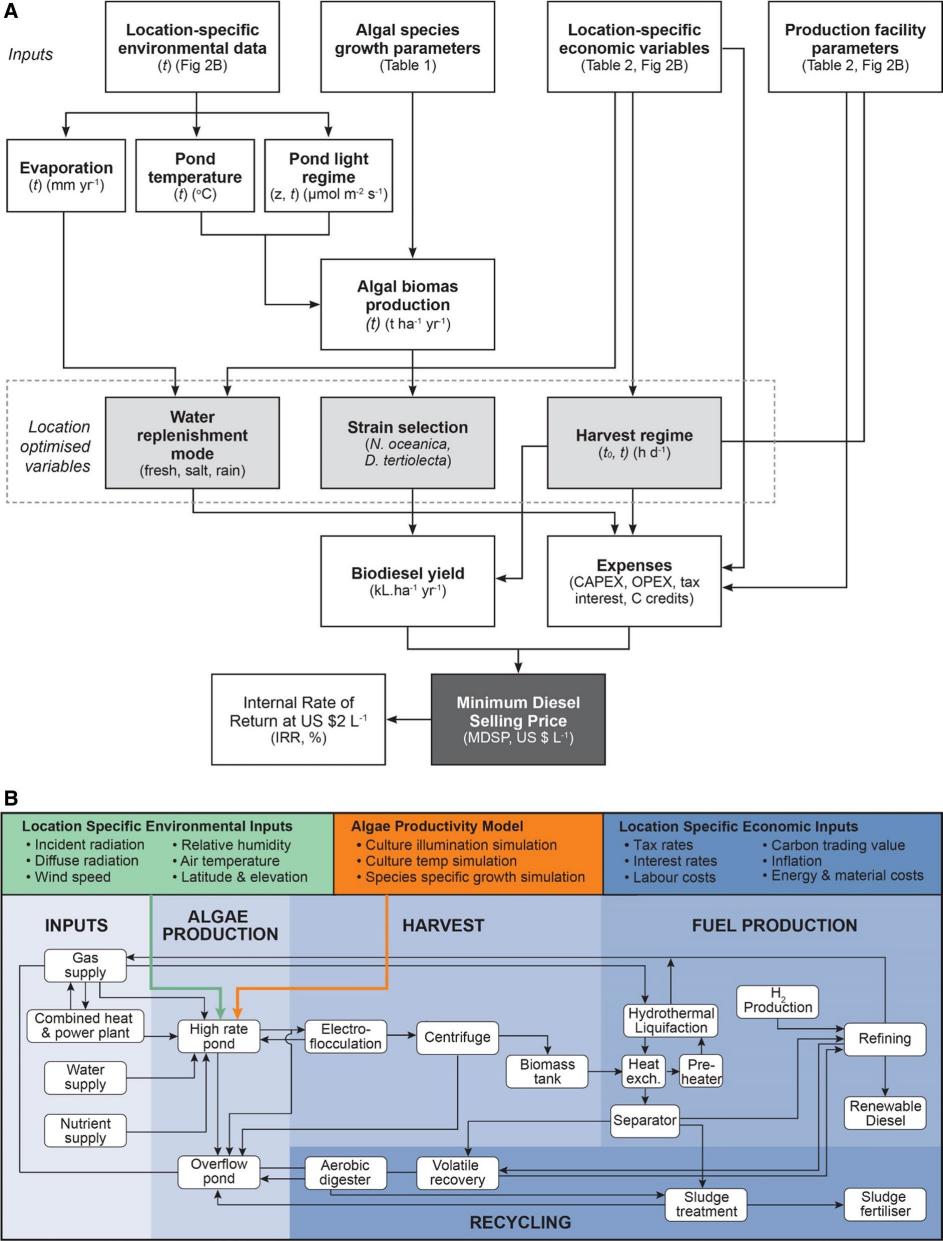
Overview of analytical framework. **a** Techno-economic calculation scheme. **b** Microalgae-based renewable diesel production process flow diagram and model inputs (modified from [12]). International Location Specific Environmental Inputs (green) and the Algae Productivity Model (orange) connect with the high-rate pond module of TELCA, to enable location, system, and strain specific growth modelling (1 h temporal resolution). Location Specific Economic Inputs (blue, top right) influence the final minimum diesel selling price and internal rate of return

The Algae Productivity Model incorporated into TELCA 2, here (Fig. 1) enables a more dynamic evaluation of spatiotemporal effects on the biological response of algae which is a critical determinant of success. One limitation of this study was the extrapolation of reported algae growth parameters to outdoor conditions. We recognize that such an approach does not take into account the many other potential factors that can affect productivity in natural systems, such as grazing, contamination, and culture crash, nor does the input weather data take into account severe weather events. However, it also does not include future improvements. The average annual values that we have calculated and used for our analyses range from 8.6 to 22.1 g m^−2^ day^−1^ and these productivities have been shown to be achievable in long-term outdoor experimental conditions [25, 30]. Future perspectives of this model are to integrate long-term actual productivity data.

The economic feasibility, biodiesel production capacity as well as embodied and process associated greenhouse gas (GHG) emissions were evaluated for 12 international locations using an expanded version of our previously reported *Techno-Economic and Life Cycle Analysis* (TELCA) tool (Fig. 1a, b) [29]. The updated TELCA2 simulation used for this study is described in detail in Additional file 1. It includes:

- *location-specific environmental inputs* (Fig. 1; Additional file 1: Section 2) to model spatio-temporal pond culture irradiance, temperature and evaporation profiles,
- the *algae productivity model* (Fig. 1, Table 1) to model growth performance under different climatic conditions (Fig. 1; orange, Additional file 1: Section 2) and
- *location specific economic inputs* (Table 2; Additional file 1: Section 1), such as the costings of capital and operational expenditure, interest, labour and tax (Fig. 1b; blue, Additional file 1: Section 1.1).

**Table 1:** Growth characteristics of *D. tertiolecta* and *N. oceanica* used for Eq. 2

		*D. tertiolecta*	*N. oceanica*
Min temp, °C [37]	*T*_*min*_	5	− 0.2
Optimum temp (max growth), °C [37]	*T*_*opt*_	32.6	26.7
Max temp, °C [37]	*T*_*max*_	38.9	33.3
max growth rate, day^−1^ [37]	*µ*_*max*_	3.5	1.85
Half saturation irradiance, μmol m^−2^ s^−1^ [39]	*K*_*s*_	58	29
Optimum irradiance, μmol m^−2^ s^−1^ [39]	*I*_*opt*_	275	203
Mass absorption coefficient, m^2^ kg^−1^ [40]	*E*_*a*_	141	178
Basal respiration rate, day^−1^ [41]	*R*_*b*_	0.2	0.2

**Table 2:** Variables and parameter inputs summary table used for TELCA (see Additional file 1 for sources)

		Darwin, Australia	Haikou, China	Chennai, India	Amsterdam, The Netherlands	Abu Dhabi, UAE	Recife, Brazil	Almeria, Spain	Mombasa, Kenya	Kona, US	Tunis, Tunisia	Izmir, Turkey	Acapulco, Mexico
Base skilled Labour rate	USD h^−1^	24.11	4.97	1.61	33.58	16.13	3.49	12.56	2.03	26.00	4.30	2.40	2.20
Rate factors													
Unskilled		78%	72%	80%	86%	73%	80%	75%	71%	80%	42%	81%	83%
Supervisor		119%	128%	140%	114%	140%	147%	110%	135%	120%	130%	114%	149%
Operator		120%	118%	150%	125%	196%	162%	121%	148%	130%	143%	126%	164%
Management		157%	175%	225%	137%	270%	206%	154%	189%	150%	182%	148%	194%
Base hours	h	38	40	42.5	39	40	40	40	40	40	40	45	48
Overtime loading		160%	100%	200%	100%	100%	150%	100%	100%	100%	100%	150%	100%
Labour efficiency		79%	54%	50%	80%	62%	55%	76%	46%	87%	57%	70%	58%
Labour non-wage costs		37%	50%	22%	20%	35%	52%	25%	20%	23%	17%	32%	17%
Contractor margin		15%	20%	20%	15%	20%	20%	15%	20%	15%	20%	20%	20%
Currency value	USD	0.75	0.15	0.01	1.17	0.27	0.27	1.17	0.010	1.00	0.38	0.21	0.054
Inflation rate	Per annum	2.1%	2.2%	4.2%	2.1%	3.8%	4.5%	2.2%	4.4%	2.9%	7.5%	15.9%	4.8%
Interest (scenario 1: for profit model)	Per annum	5.4%	8.7%	11.2%	3.7%	6.3%	11.2%	3.7%	14.0%	6.0%	11.4%	24.0%	12.6%
Government bond rate (scenario 2: public utility model)	Per annum	2.7%	3.5%	7.8%	0.5%	4.2%	11.0%	1.4%	13.2%	2.9%	6.6%	17.7%	7.8%
Company tax rate		29%	25%	30%	25%	0%	34%	25%	30%	27%	25%	22%	30%
Carbon price	US$ T^−1^CO_2_e	0	0	5.85	8.20	0	0	8.20	0	0	0	0	3.5
Land price	USD ha^−1^	2250	7500	18,296	66,731	8212	4091	27,623	6250	9063	998	42,000	6250
Electricity price	USD kWh^−1^	0.16	0.14	0.09	0.10	0.01	0.11	0.11	0.06	0.03	0.06	0.07	0.27
Water price	USD kL^−1^	0.06	0.075	0.04	0.22	0.86	0.02	0.12	0.08	0.06	0.05	0.02	0.03
Steel fabrication price	USD T^−1^	2107	1741	1296	2400	1943	2019	2540	1895	2303	1910	1807	1962
Average Irradiance	kWh m^−2^ day^−1^	5.78	4.43	5.47	3.02	5.97	5.89	4.93	5.48	5.28	4.83	4.68	5.34
Average rainfall	mm year^−1^	1730	1652	1392	838	57	2418	200	1059	467	519	718	1517
Average evaporation	mm year^−1^	2139	1419	1689	450	2235	1239	1585	1408	1856	1330	1482	1522
Algae species cultivated		Dun	Nanno	Dun	Nanno	Dun	Dun	Nanno	Nanno	Dun	Nanno	Nanno	Dun

Algae strain: *Dun.* = *Dunaliella tertiolecta, Nanno* = *Nannochloropsis oceanica*

Using this information systems were optimized to reduce MDSP at each location.

Two business case scenarios at each location were assessed: a standard commercial *for-profit business model* (Scenario 1); and a *public utility not-for-profit model* (Scenario 2). For Scenario 1, economic feasibility was calculated using the Internal Rate of Return (IRR, %) based on the difference between the MDSP and a fixed theoretical diesel selling price of US$ 2 L^−1^. For Scenarios 1 and 2, the MDSP is reported (Table 3). Microalgae-based biodiesel production capacity is defined as kL diesel ha^−1^ year^−1^ based on optimized conditions for biomass production and harvesting regimes which resulted in the lowest MDSP.

### CO_2_ emissions

*CO*_*2*_* emissions* were calculated from CO_2_ (gCO_2eq_ MJ^−1^) absorbed during the overall photosynthetic biomass production and fuel production processes, offset against the amount of fossil-based CO_2_ released during construction (e.g., via embodied emissions in the construction, equipment supply and supply of consumable items), operation of the facility (external CO_2_ supply for biomass production—11% CO_2_ concentration (Additional file 1: Appendix 1), and embodied CO_2_ emissions in the production and supply of nutrients), as well as emissions from subsequent fuel use. To minimise emissions, the model has been structured around a fully self-sufficient energy design (i.e., all of the energy required to operate the plant including electro-flocculation and hydrogen production was produced internally with solar PV (Additional file 1: Section 3). All emissions have been fully incorporated into the net energy and CO_2_ accounting, and balanced over the productive life of the facility (30 years). Reduction in emissions was assessed as the difference between the overall emissions from the process and emission from conventional fossil-based diesel fuel production and use, that it displaces (i.e., displaced fossil fuel (gCO_2eq_ MJ^−1^)—renewable diesel (gCO_2eq_ MJ^−1^) = CO_2_ emission reductions (gCO_2eq_ MJ^−1^).

### System boundaries and key assumptions

Simulations were performed for a facility comprising 177 high-rate microalgae production ponds of 4.27 ha each, (total pond area = 500 ha), on-site harvest, processing and refining facilities (Fig. 1b, Additional file 1). Algal biomass was harvested using electro-flocculation and concentrated via centrifugation (Additional file 1: Section 3), before being converted to crude oil via hydrothermal liquefaction (HTL) with a biomass to green crude conversion of 55% [12, 31, 32] (Additional file 1: Appendix 1). Renewable diesel was refined using conventional hydrotreatment/hydrocracking and fractionation processes [12] (Additional file 1: Section 3). Based on reported values [33] 75% nitrogen recovery was assumed in the HTL aqueous phase with nutrients further treated via anaerobic digestion. Overall, the model allowed for 40% of all nutrients to be recycled back to the high-rate ponds [12], where they have previously been reported to support good growth rates [34] (Additional file 1: Appendix 1). CO_2_ supply was taken from a free issue source (11% CO_2_ concentration) (Additional file 1: Appendix 1) immediately adjacent to the production facility with all piping, cooling, filtration, and compression accounted for in the cost analysis (Additional file 1: Section 3). CO_2_ was supplied to the algae culture at a concentration of 1% and utilisation efficiency was set to 80% (Additional file 1: Appendix 1). Nutrients were assumed to be non-limiting to growth. A complete description of assumptions and boundary conditions is provided in Roles et al. [12, 29] with advanced components and modifications detailed below and in Additional file 1: Sections 1–3.

### Location selection

Twelve geographical locations were selected across North and South America, Europe, Africa, the Middle East, India, Asia and Oceania (Table 2, Graphical Abstract) for comparative analyses. Sites were selected to cover a broad range of irradiance levels, temperatures and other climatic conditions and economic variables. All sites were chosen, because they provide access to seawater, suitable land and topography (low slope, low density or undeveloped) within a 100 km radius.

### Productivity modelling

Under non-limiting nutrient conditions, light and temperature are the most important variables affecting photosynthetic algal growth and the resultant yield of biomass. Light and temperature regimes vary widely between geographical locations and over time due to daily and seasonal cycles. To account for dynamic fluxes in light, temperature and growth, algal biomass productivity was modelled at 1 h intervals using typical weather data over 365 days of the year for each location. Input variables included: global horizontal radiation (W m^−2^), diffuse horizontal radiation (W m^−2^), wind speed (m s^−1^), relative humidity (kg kg^−1^) and air temperature (°C, *EnergyPlus*, US Department of Energy and the National Renewable Energy Laboratory, US). These inputs were used in a heat balance model to predict changes in culture media temperature [35] (Additional file 1: Methods, Section 3). Diffuse and global solar radiation values were used to predict light transfer through the culture [36].

The temperature of the pond’s liquid culture was predicted using a simplified mechanical heat balance described by Bechet et al. [35]. Although temperature gradients within the liquid phase can occur, the culture temperature is assumed to be homogenous due to paddlewheel mixing and gas supply. In contrast, the exponential decay of light as it is attenuated by algal pigments through the depth of the culture results in a light gradient ranging from photo-inhibitory light at the pond surface to photo-limited or dark areas toward the pond base. This causes specific growth rates to differ through the culture. Here, we modelled local irradiance along the optical pathlength (i.e., from the pond surface to the base), using a simple and validated radiative transfer model described by Lee et al. [36] that accounts for both direct beam radiation and diffuse, or scattered radiation. Hourly predictions of pond culture temperature, *T*_pond_ (*t*) (°C) and local irradiance through the pond depth *I*_loc_ (*t*, *z*) (μmol m^−2^ s^−1^) were used to predict the specific growth rate of algae using the light and temperature dependent algae growth model described by Bernard and Remond [37]. Growth rates were integrated over time, *t*, and pond depth, *z* (m^−1^) to estimate volumetric productivities. Productivity modelling algorithm development and simulations were performed in MATLAB (R2015b, MathWorks).

#### Governing equations

The full model algorithm is outlined in Additional file 1: Section 2. During the growth phase, the volumetric biomass productivity of the system, *P*_vol_ (g biomass dry weight L^−1^) was determined by the rate of change of the algal biomass concentration over time:

1 $$P_{{{\text{vol}}\left( t \right)}} = \frac{{{\text{d}}C_{x} }}{{{\text{d}}t}} = \overline{\mu }C_{x} - C_{x} R$$

where *C*_*x*_ is the biomass concentration (g L^−1^), *µ* is the specific growth rate (h^−1^) and *R* is the basal respiration rate (h^−1^). According to Bernard and Remond [37], *µ* is a function of irradiance and temperature:

2 $$\mu \left( {T,I} \right) = \mu_{{{\text{max}}}} \frac{{I_{{{\text{loc}}}} }}{{\frac{{\mu_{{{\text{max}}}} }}{\sigma }\left( {\frac{{I_{{{\text{loc}}}} }}{{I_{{{\text{opt}}}} }} - 1} \right)^{2} }} \Phi \left( T \right).$$

In Eq. 2., *µ*_max_ is the maximum growth rate of a given species (day^−1^); the light response parameters *σ* and *I*_opt_ define the irradiance values (µmol m^−2^ s^−1^) at half saturation rate of photosynthesis (µmol m^−2^ s^−1^) and at maximum growth, respectively; and *Φ* is the proportional effect of temperature (dimensionless), using the inflexion function of Rosso et al. [38]:

3 $$\Phi \left( T \right) = \frac{{\left( {T - T_{\max } } \right) \left( {T - T_{\min } } \right)^{2} }}{{\left( {T_{{{\text{opt}}}} - T_{\min } } \right) \left[ {\left( {T_{{{\text{opt}}}} - T_{\min } } \right)\left( {T - T_{{{\text{opt}}}} } \right) - \left( {T_{{{\text{opt}}}} - T_{\max } } \right)\left( {T_{{{\text{opt}}}} + T_{\min } - 2T} \right)} \right]}}$$

In Eq. 3, the parameters *T*_opt_, *T*_min_ and *T*_max_ represent three cardinal temperatures of biological significance, these being, respectively, the optimal temperature at which growth is highest at a given irradiance, and the minimum and maximum temperatures which define the threshold beyond which no growth occurs (Eq. 4):

4 $$\mu _{{\max }} = \left\{ {\begin{array}{*{20}l} 0 & {{\text{for}}~T < T_{{\min }} } \\ {\mu _{{\max }} \cdot \Phi \left( T \right)} & {{\text{for}}~T_{{\min }} < T < ~T_{{\max }} } \\ 0 & {{\text{for}}~T > T_{{\max }} } \\ \end{array} } \right.$$

To predict local irradiance, *I*_(*z*)_ along the culture depth, we use the simple two-flux approximation of light transfer for open ponds (Eq. 5) proposed by Lee et al. [36]:

5 $$I_{\left( z \right)} = I_{B\left( z \right)} + I_{D\left( z \right)} .$$

In Eq. 5, *I*_*B*(*z*)_ and *I*_*D*(*Z*)_ are the direct beam irradiance and diffuse irradiance, respectively, at a given point, *z,* through the reactor depth, L (m^−1^), with 0 being the illuminated surface, and

6 $$I_{B\left( z \right)} = I_{B} e^{{ - \frac{{\alpha C_{x} }}{{{\text{cos}}\left( \theta \right)}}z}}$$

7 $$I_{D\left( z \right)} = 2I_{D} e^{{ - 2\alpha C_{x} Z}} ,$$

where *α* is the mass extinction coefficient of the algae (m^2^ kg^−1^, averaged across the 400–700 nm photosynthetically active radiation range), and *θ* is the zenith angle of direct beam radiation hitting the surface of the pond.

The culture temperature was predicted using a heat flux model that provides an overall energy balance defined by *Q* (W), such that the change in temperature of the liquid media is defined as

8 $$\frac{{{\text{d}}T}}{{{\text{d}}t}}V\rho c_{p } = Q_{{{\text{solar}}}} + Q_{{{\text{evaporation}}}} + Q_{{{\text{thermal}}}} + Q_{{{\text{conduction}}}} ,$$

where the heat fluxes are solar radiation, *Q*_solar_ (W), evaporation, *Q*_evaporation_ (W), thermal radiation at the pond surface between the air and the water, *Q*_thermal_ (W) and conduction to the soil, *Q*_conduction_ (W).

#### Algal species selection

**Fig. 2:**
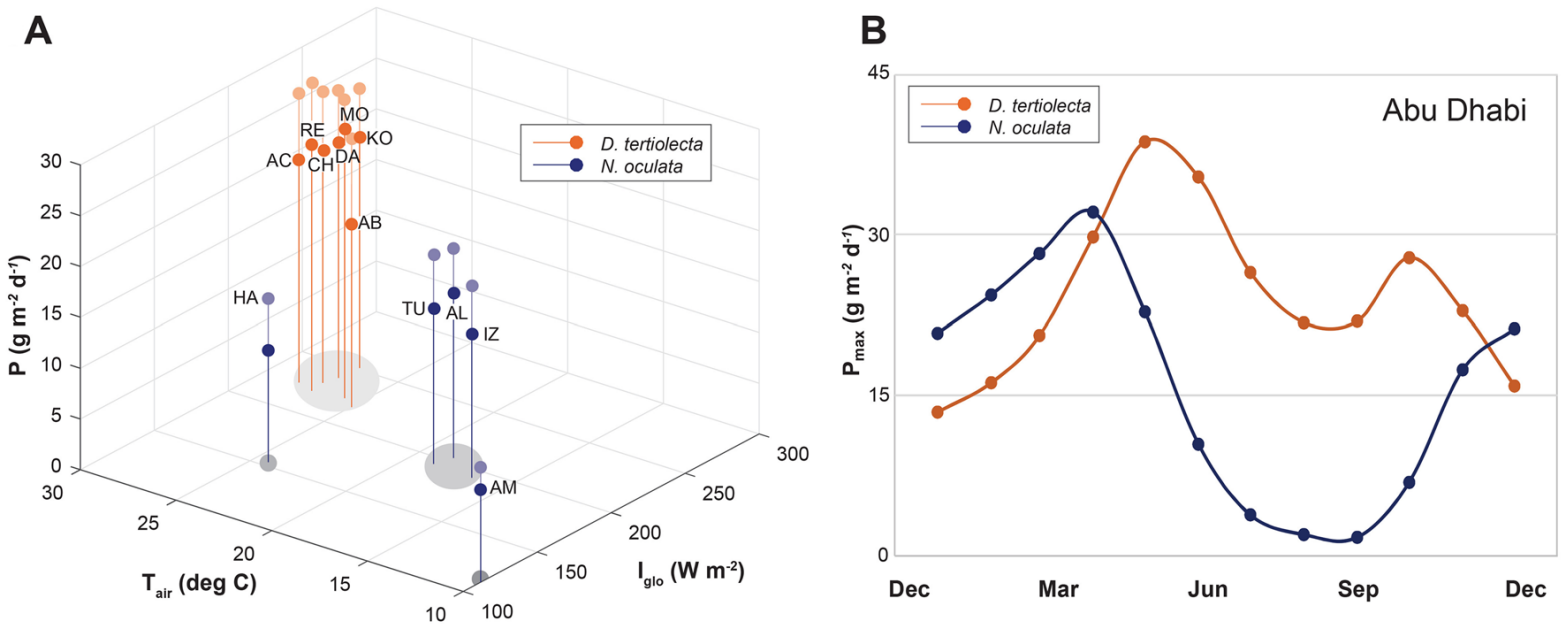
**a** Maximum and average location specific productivities as a function of solar irradiance and temperature for the best performing strain. Maximum productivity refers to the biomass productivity optimized for yield (Light blue: *N. oculate; Light* orange: *D. tertiolecta)* at 0.3 m pond depth and optimal operating biomass concentration (0.1 g L^−1^)**.** *Average productivity* refers to the biomass productivity optimized for IRR in the techno-economic evaluation (Dark blue: *N. oculate;* Dark orange: *D. tertiolecta*) to ensure a conservative techno-economic modelling position (8.6–22.1 g m^−2^ day^−1^; see also Fig. 1 and Table 3) *AB* Abu Dhabi, United Arab Emirates; *AC* Acapulco, Mexico; *AM* Amsterdam, Netherlands; *AL* Almeria, Spain; *CH* Chennai, India; *DA* Darwin, Australia; *HA* Haikou, China; *IZ* Izmir, Turkey; *KO* Kona, USA; *MO* Mombasa, Kenya; *RE* Recife, Brazil; *TU* Tunis, Tunisia; The light to dark grey shaded circles represent high to low annual irradiance levels. **b** Strain specific productivity in Abu Dhabi illustrates the benefit of dual strain cultivation over an annual cycle

Two industrially relevant marine microalgae species were chosen, *Nannochloropsis oceanica* and *Dunaliella tertiolecta*. Both strains exhibit high autotrophic growth rates, a lipid content of ~ 30–40%, and tolerance to wide ranges of temperature and high salinity [41–44]. This is particularly important for operations under high evaporation conditions which can result in rapid increases in salt content, up to double that of seawater. The growth response parameters to temperature and light (Eq. 2) were characterized and validated by Bernard and Remond [37], providing the coefficients listed in Table 1. *N. oceanica* exhibits optimal growth at a lower optimal temperature and light intensity compared to *D. Tertiolecta*, suggesting that these species will perform better under temperate and tropical conditions, respectively. For each location, productivity simulations were performed for each strain. The alga exhibiting the highest productivity at each location under the range of conditions analyzed was used for the results reports (Fig. 2, Table 2).

#### Productivity model validation

The three models used to estimate productivity (liquid culture temperature; local irradiance; and light- and temperature-dependent algal growth) have been previously validated within acceptable ranges against experimental data sets. Lee et al. [36] showed that the simple two-flux approximation predicted local irradiance in a photobioreactor with a variation of 2–13% compared to more complex radiative transfer models, depending on the time of the day. To ensure the accuracy of our model algorithm, we validated radiative transfer with their reported modelled predictions. The simple radiative transfer equation has been widely used within the literature to estimate light mediated growth. Moreover, Lee et al. [36] found that such differences in estimated PAR resulted in productivity estimations within a 2–10% variation.

For prediction of temperature of the algal culture, Bechet et al. [35] validated the heat transfer model (Eq. 8) with an accuracy of 2.4 °C against experimental data collected over a 28-day period consisting of 108 temperature measurements taken from the liquid culture of an outdoor 50 L column photobioreactor in Singapore. Because of the complexity of the various heat components of the model, we compared our model simulations against experimental temperature measurements taken within the culture of two 2000 L ponds at the Centre for Solar Biotechnology Pilot plant, Brisbane (Additional file 1: Section 2). The model produced a tight fit between the measured and predicted media temperature in both ponds over a 6-day period, (*R*^2^ ≥ 0.9).

The simulations of algal growth for *D. tertiolecta* and *N. oceanica* were compared with those against actual data by Bernard and Remond [37] for the species used in this study.

Beside strain selection, simulations were performed for variables of pond depth (0.1–0.3 m) and quasi-steady-state operating biomass concentrations (0.05–1 g BDW L^−1^). The former affects thermal mass and light regime and latter effects light regime (heat dissipation from algae is considered negligible). Algal productivity modelling algorithm development and simulations were performed in MATLAB (R2015b, MathWorks). All productivity simulations were exported from MATLAB as tables into the TELCA model to optimize harvest regime, depth and concentration to MDSP.

### Viability & feasibility

Under a for-profit business model (Scenario 1), the economic effectiveness of algae diesel was assessed using the Internal Rate of Return (IRR) over the life of the facility at a fixed product price. Here, IRR is calculated for each location based on a hypothetical fixed Minimum Diesel Selling Price (MDSP) of US$ 2.00 L^−1^. For Scenario 2, the feasibility was assessed on the cost-competitiveness of the MDSP that could be achieved. In this not-for-profit public utility scenario, profit and tax rates were reduced to zero. Interest rates were reduced from commercial rates to match government bond rates prevailing at each location (Table 2). The resulting MDSP was benchmarked between locations and against existing fossil fuel prices.

### Optimization

Optimization was performed at each location to minimize the MDSP for the following variables: algal strain selection (based on the highest annual-averaged productivity); freshwater replenishment for evaporation (MDSP minimized based on CapEx (e.g., piping, storage) and OpEx (e.g., water purchase, blowdown) requirements over the 30-year lifespan of the facility); operational algae concentration; and harvest duration (MDSP minimized based on CapEx and OpEx over the 30-year lifespan of the facility).

### Boundary conditions

*High-rate pond depth and concentration* The pond depth, harvest duration and steady-state biomass concentration were the primary set of optimized variables adjusted monthly to optimize MDSP for the entire production, harvest and product processing system. A fixed, rather than variable, harvesting rate was set for the operation as the extra cost for variable speed equipment could not be economically justified. Daily harvest duration was adjusted to optimize culture density for MDSP. In all cases optimum MDSP was obtained by minimizing pond depth. This optimisation, however, was limited to a minimum of 0.25 m by engineering constraints of the capacity to construct and operate very shallow depth ponds in conjunction with large 4.3 ha pond areas (see Additional file 1: Appendix 1).

*Water replenishment* Three options to balance evaporative losses after accounting for available rainwater were analyzed (Additional file 1: Section 1). Essentially, incorporation of water storage based on a percentage of total pond capacity, replacement with locally purchased fresh water, or replenishment with seawater which necessitates further discharge of pond water (blowdown) to avoid excess salt build-up was analyzed. Blowdown also results in the loss of valuable nutrients from the system. The ideal replenishment choice was location dependent and detailed in the results, but in each case was optimised based on the MDSP.

*Tax rates* Tax rates applied were corporate only, and did not include value added taxes. The latter, however, may have an impact in some jurisdictions (Table 2).

*Labour rates* were based predominantly on the tradingeconomics.com/labour-costs website. Rates were established for skilled labour and relative rates were then found for a range of labour categories (Table 2). The categories were identified and estimated for all construction and operational tasks. Base working hours, overtime loadings and non-wage costs were established from various sources (Additional file 1: Section 1).

*Labour efficiency* was primarily based on GDP per hour worked data, provided in World Bank and OECD databases (Additional file 1: Section 1). GDP output per hour worked for the construction industry differs from these numbers but construction specific and consistent data was only available for European Union countries. For labour efficiency (Table 2), the ratio between whole of economy and the construction sector from the Euro zone countries was assumed to be similar in all countries and was thus used for modelling.

*Employment and labour costs* The 500 ha microalgae facility simulation is based on a set of interconnected process modules (Fig. 1b). Each process module accounts for the associated construction and operation tasks. The work required (and associated cost) to complete each task is calculated based on a *fixed labour component* and *process variable labour component*. The component variable base was selected based on the most applicable process variable to each task (e.g., *Pond Area* for pond cleaning, *Flow Rate* for filter cleaning, Additional file 1: Appendix 1).

*Project finance rates* applicable to a variety of project types, conditions and risk profiles is generally regarded by industry participants as commercial in confidence. The project interest rates modelled here (Table 2) represent the rates applicable to well established technology being operated by a financially sound project proponent. To provide a broad approach for determining these rates a relationship between government benchmark interest rates and project finance rates was established from known data in the solar PV industry[42].

*The costs of supplied construction materials* were divided into two groups; fabricated items the price of which was determined in accordance with local labour costs and efficiencies, and equipment supply that would be purchased at internationally competitive rates [29].

*Rates for currency, inflation, water, land and electricity* are detailed in Table 2 and sources are detailed in Additional file 1: Section 1.

## Results

A summary of projections related to technoeconomic, productivity and emissions performance for each location is presented in Table 3, with detailed results discussed below.

**Table 3:** Summary results of techno-economic analysis by location

	TEA summary			Productivity (annual avg)			Carbon emissions	
	For-profit (S1)		Public utility (S2)	DW biomass	Max. DW biomass	Biodiesel	CO_2eq_	Reduction to fossil
	IRR	MDSP before profit	MDSP					
	(at US$2 L^−1^)	US$ L^−1^ (US$ gal^−1^)	US$ L^−1^ (US$ gal^−1^)	g m^−2^ day^−1^	g m^−2^ day^−1^	kL ha^−1^ year^−1^	g MJ^−1^	
Mombasa (KEN)	22.1%	$1.60 ($6.04)	$1.24 ($4.69)	22.1	29.1	44.7	25	− 72.8%
Acapulco (MEX)	20.8%	$1.54 ($5.82)	$1.17 ($4.42)	20.0	28.7	40.4	26	− 71.7%
Chennai (IND)	20.2%	$1.51 ($5.70)	$1.15 ($4.35)	19.8	28.4	40.0	25	− 72.8%
Recife (BRA)	19.0%	$1.57 ($5.93)	$1.19 ($4.50)	21.9	29.9	44.3	27	− 70.7%
Darwin (AUS)	13.2%	$1.53 ($5.80)	$1.23 ($4.67)	21.7	28.4	43.8	26	− 71.7%
Tunis (TUN)	13.3%	$1.55 ($5.87)	$1.24 ($4.68)	15.0	20.6	30.3	28	− 69.6%
Kona (USA)	13.0%	$1.53 ($5.79)	$1.24 ($4.68)	21.0	26.8	42.4	27	− 70.7%
Abu Dhabi (UAE)	11.5%	$1.54 ($5.84)	$1.47 ($5.58)	17.5	24.3	35.3	30	− 67.4%
Almeria (SPA)	10.9%	$1.48 ($5.61)	$1.23 ($4.66)	15.8	20.6	31.9	28	− 69.6%
Haikou (CHN)	9.5%	$1.85 ($6.99)	$1.59 ($6.01)	11.5	16.2	23.3	37	− 59.8%
Izmir (TUR)	1.0%	$2.57 ($9.73)	$2.21 ($8.35)	13.8	18.8	27.8	27	− 70.7%
Amsterdam (NDL)	− 7.0%	$2.79 ($10.55)	$2.61 ($9.87)	8.6	11.3	17.4	37	− 59.8%

### Optimisation of processes to minimise the diesel selling price

#### Matching the algae strain to suit the production location can significantly improve productivity

**Fig. 4:**
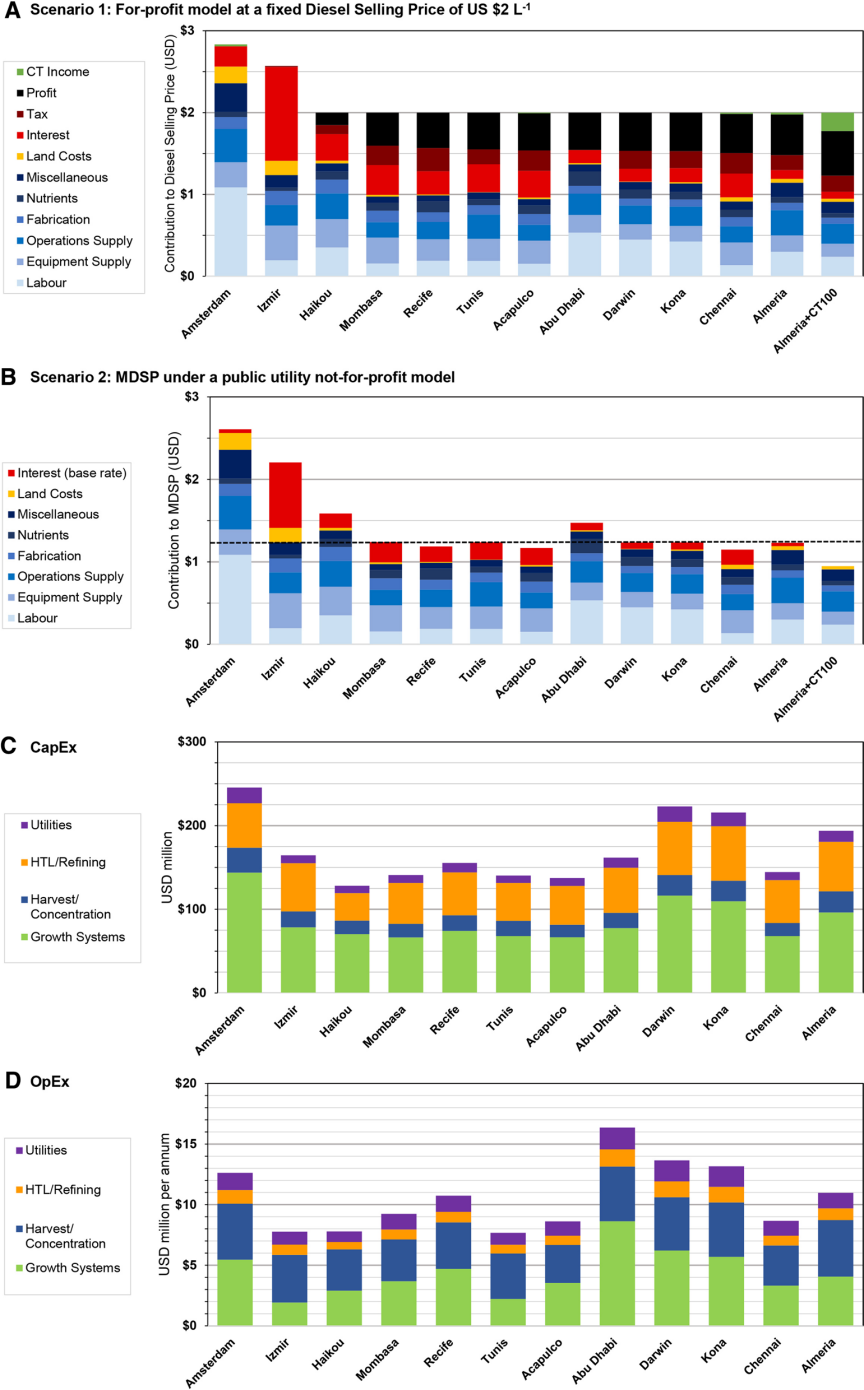
Breakdown of the key components contributing to the minimum diesel selling price (MDSP). **a** Scenario 1 (for profit model) shows that 10 locations are profitable at an MDSP of US$ 2 L^−1^. **b** Scenario 2 (public utility model, not for profit) includes production system costs (blue), land and misc. (yellow), and low interest (red) with 0% tax and 0% profit. Under this scenario, 8 locations (Mombasa, Kenya, Recife, Brazil; Tunis, Tunisia; Acapulco, Mexico; Darwin, Australia; Kona, USA; Chennai, India and Almeria, Spain) could achieve an MDSP of < US$ 1.25 (under black dotted line), almost at parity with maximum historical fossil diesel prices. Far right: shows an improvement in MDSP and profitability that could be achieved in Almeria (Spain) if the EU adopted a carbon tax (CT) of US$ 100 tonne^−1^. *Interest figures shown in this figure represent total interest payments over a 10-year loan repayment period at respective interest rates (values in Table 2). The inclusion of a carbon price in Scenarios 1 and 2 reduces the contribution of each costed item to the MDSP. **c** CapEx breakdown of the 500 ha facility ranged between US$ 128–245 Million (i.e., US$ 256,000–490,000 ha^−1^), **d** Annual OpEx ranged between US$ 7.7–16.4 Million (i.e., US$ 15,400–32,800 ha^−1^)

The *Algae Productivity Model* computed hourly growth rates as a function of solar irradiance and culture temperature, based on actual weather data. Simulations were performed over pond depths of 0.1–0.3 m and operating quasi-steady-state biomass concentrations ranging from 0.05 to 1 g L^−1^ at each location (Additional file 1: Figures S4–17). Figure 2a summarizes the simulated maximum (11.3–29.9 g m^−2^ day^−1^) and average (8.6–22.1 g m^−2^ day^−1^) productivities of the best performing algal species at each location. The annual-average range (8.6–22.1 g m^−2^ day^−1^) biomass corresponds to 31.4–80.7 T ha^−1^ year^−1^. For most locations, higher biomass productivities could be achieved at a greater depth of 0.3 m, as this provided more stable temperatures and reduced extreme fluctuations, but only under more dilute operational concentrations (≤ 0.1 g L^−1^). However, the economic optimization identified a 0.25 m depth and a higher operating concentration to reduce harvesting costs (see below). As expected, several near-equatorial locations (e.g., Mombasa, Kenya; Recife, Brazil; Acapulco, Mexico; Darwin, Australia; and Kona, USA) exhibiting relatively high irradiance and air temperature yielded the highest annual-average productivities between 20.0 and 22.1 g m^−2^ day^−1^. These values are within the range of achievable biomass yields in high-rate pond systems [25, 30]. Abu Dhabi (United Arab Emirates—UAE), also in this cluster, had lower average yields (17.5 g m^−2^ day^−1^) due to its desert climate of extreme temperatures and irradiance. A second cluster is shown for the sub-tropical locations of Tunis, Tunisia; Almeria, Spain; and Izmir, Turkey at ~ 13–16 g m^−2^ day^−1^. Haikou (China), with its high temperature but relatively lower irradiance due to relatively high rainfall yielded 11.5 g m^−2^ day^−1^ and the cool, temperate climate of Amsterdam (Netherlands) yielded the lowest at 8.6 g m^−2^ day^−1^. The alga *D. tertiolecta* (Fig. 3a, orange circles) performed best in equatorial regions that had consistently high temperatures, while *N. oceanica* (Fig. 2a, blue circles) performed better in locations with cooler climates and lower irradiance. Locations that had a broader temperature range over the year exhibited reduced productivities compared with less variable locations (e.g., Kona, USA and Recife, Brazil) using a single strain (Fig. 2a). For example, in the desert climate of Abu Dhabi (UAE), *N. oculata* exhibited higher productivity through winter (Dec–March), while *D. tertiolecta* performed significantly better in the summer (Mar–Nov) (Fig. 2b). For the technoeconomic evaluation (Fig. 4), average productivity values were used for the highest yielding strain (i.e., *D. tertiolecta* or *N. oceanica*) for each location to ensure a conservative modelling position. These results indicate that for certain locations, different strains could be used seasonally to improve yields.

**Fig. ﻿3:**
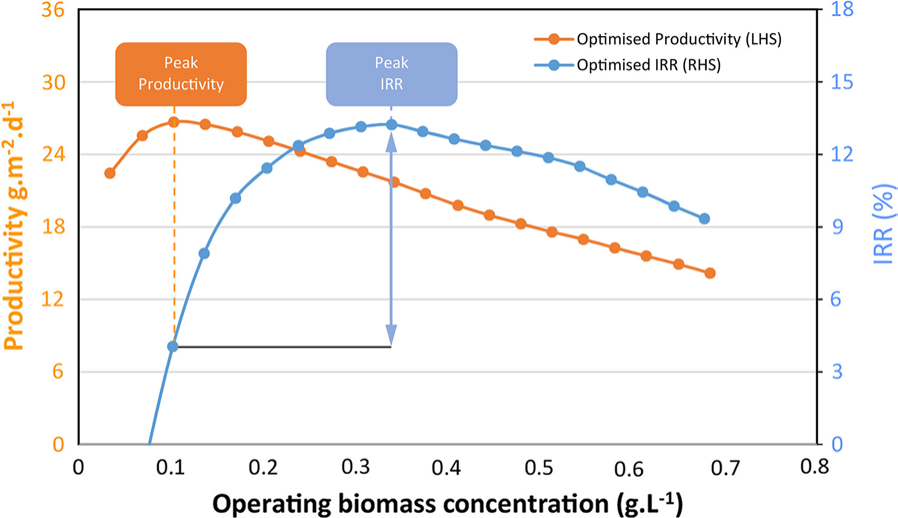
Comparison of systems optimized for productivity and IRR in Darwin, Australia. The blue arrow indicates an increase in IRR from ~ 4% (Peak productivity settings) to ~ 13% (Peak IRR settings)

#### The trade-off between productivity and harvest costs to minimise diesel selling price

Late afternoon semi-continuous harvesting is considered *optimal for productivity*, as it minimises biomass loss via respiration in the dark [43]. While this principle is correct, *financially optimised* systems (Fig. 3, Peak IRR) require the minimisation of harvesting CAPEX (i.e., the smallest harvesting system operated for the maximum duration). In addition, the lowest cost system involves fixed rate harvesting and so can only be adjusted through the *start time* and *operational duration.* At an industrial scale, harvesting is usually conducted continuously, to minimise the CapEx of the harvesting equipment (i.e., longer harvesting times = smaller harvest system requirements), with the proviso that this results in a net improvement in the MDSP. The TELCA model has been constructed to conduct such cost benefit analysis and to determine the optimum harvesting regime. We, therefore, focused on minimising the non-harvesting periods, to keep harvesting CapEx low. For all months and at all locations stopping harvest in the morning was found to be beneficial as it allowed cell numbers to increase rapidly during the morning, while harvesting from the afternoon onwards allowed harvesting at higher cell densities, making the process more efficient. Collectively, this high culture density/low harvesting CapEx strategy, yielded a better MDSP and IRR (Fig. 4, Table 3). This financially optimised system (Darwin, Australia) increased IRR from ~ 4% (at the peak productivity setting, ~ 0.1 g biomass dw L^−1^ operating concentration) to 13% (at a 3.5 fold higher operating concentration, ~ 0.35 g biomass dw L^−1^).

Productivity increased with pond depth (g m^−2^) in most locations, but IRR decreased. The use of shallower ponds (i.e., 25 vs. 30 cm) with higher concentration reduced harvesting costs. Construction and thermal stability constraints for large shallow ponds limited further depth reductions.

#### Balancing evaporation and water replenishment is important for optimal IRR and freshwater use

Saltwater systems are designed to minimise their environmental freshwater-use footprint. Ideally, evaporated water is replenished with rainwater but in practice an imbalance usually exists and must be corrected. Three options to balance evaporative losses after accounting for available rainwater were analyzed: (1) incorporation of water storage based on a percentage of total pond capacity; (2) replacement with locally purchased fresh water; or (3) replenishment with seawater which necessitates further discharge of pond water (blowdown) to avoid excess salt build-up, but also results in the loss of valuable nutrients from the system. For all locations, the option of water storage between rain events proved to be the *least* economic due to added CapEx and land requirements, and consequently required a combination of freshwater and seawater replenishment. For financial optimisation, the proportion of freshwater purchased vs. new seawater added with blowdown to maintain salinity was location dependent, ranging from 0% freshwater purchase in Abu Dhabi (UAE), where the cost of freshwater is high (US$ 0.86 kL^−1^), to 96% in Chennai (India), where the freshwater price is low (US$ 0.04 kL^−1^) (see Table 2). Consequently, the high discharge rates in Abu Dhabi resulted in an approximate twofold increase in nutrient costs compared to most of the other locations analyzed (Fig. 4a).

### Liquid fuel production capacity

Hydrothermal liquefaction-based biorefinery methods have shown conversion rates of total biomass to oil at an efficiency of 55% [31, 32] which is significantly higher than processes based on traditional oil extraction (e.g., Tri-acyl glyceride extraction). Based on the simulated average biomass productivities (8.6 g m^−2^ day^−1^ to 22.1 g m^−2^ day^−1^, see Fig. 2a, Table 3) and downstream processing, this equates to biodiesel yields ranging from 17 kL ha^−1^ year^−1^ (Amsterdam, Netherlands) to 44.7 kL ha^−1^ year^−1^ (Mombasa, Kenya). A summary of reported oil yields among 20 studies [44], showed estimations of algal-based biofuel ranging from 10 kL up to 130 kL ha^−1^ year^−1^; however, the vast majority of studies ranged from 15 to 60 kL, suggesting that our indications are in the mid-range of previous reports.

### Technoeconomic evaluation

#### Scenario 1—For-profit business model

Under a commercial for-profit business model scenario (Fig. 4a), the IRR is calculated for the described algae biodiesel production system at each of the 12 chosen locations on the basis of a US$ 2 L^−1^ MDSP (Graphical abstract, Table 3). This enables the identification of specific cost components that can be further optimized to drive the MDSP down towards cost parity with fossil-fuel-based diesel. At all locations, the *proportional contribution* (US$ L^−1^) rather than the *absolute cost* (US$ system^−1^) of each component is shown. To agglomerate construction and operational costs, all future costs were discounted at inflation-adjusted interest rates prevailing in each jurisdiction (Table 2). IRR values are presented for a full *for-profit business model* (at an assumed US$ 2 L^−1^ Minimum Diesel Selling Price (MDSP) at the factory gate) and range from –7% (Amsterdam, Netherlands) to close to 22.1% (Mombasa, Kenya), with four locations presenting an IRR > 18% (Table 3).

#### Scenario 2—not-for-profit public utility model

*The not-for-profit public utility model* excludes the need to generate profit and assumed 0% IRR, 0% tax and a base interest rate aligned to respective government bond rates. The achievable Minimum Selling Price and component breakdowns are shown in Fig. 4b. The achievable MDSP under a public utility model ranged from US$ 1.15–2.61 L^−1^ (US$ 4.35–9.87 gal^−1^): i.e., Chennai, India and Amsterdam, Netherlands.

#### Production component analysis

*Labor cost* in terms of the final MDSP ranged from 6.8% in Chennai to 38% in Amsterdam (Fig. 4a). It includes direct-wage costs, non-wage on-costs and labor productivity in each location (Table 2). Process automation could significantly impact the final product price.

*Equipment supply and operational supply costs* were reasonably consistent (21–33% of MDSP) across all jurisdictions (Fig. 4a), being based on international supply prices. The differences were predominantly due to different production rates and applicable discount rates (Table 2). It is not anticipated that major savings can be made here but incremental improvements are possible.

*Fabrication* (4–8% of MDSP) was modelled to occur in each local jurisdiction, except the Netherlands, Spain, USA and Australia (Fig. 4a). Importation of fabricated items (e.g., steelwork) into these excepted countries from lower cost centers resulted in minor real cost differences across the range. Notably, high discount rates reduce the contribution of future costs and consequently increase the impact of fabrication costs associated with upfront construction. In some countries (e.g., Turkey, Brazil and Kenya) this effect had a notable impact. Consequently, it is anticipated that any savings in fabrication will have little impact on MDSP.

*Nutrient costs* (2–9% of MDSP) are generally directly proportional to the biomass production in each location (Fig. 4a). The major exception to this was Abu Dhabi, where high evaporation rates required increased saline discharge resulting in high nutrient losses. The use of strains able to grow in hyper saline conditions can help to reduce nutrient losses and freshwater costs as discharge between rain events can potentially be reduced.

*Land costs* were a comparatively small contributor to overall MDSP and viability (Fig. 4a) but ranged from 0.2% in Tunisia and Australia, to 6.8% in Turkey (influenced by the discount rate) and 7.2% in the Netherlands. These figures represent the current value of suitable land. Ultimately microalgae system deployment, however, may be affected by the absolute land availability in some locations, such as Europe, USA and China. It is anticipated that significant savings in land costs are unlikely.

*Miscellaneous* remaining costs (e.g., insurance and land use charges) were small and had little effect on the MDSP.

Overall, the average production costs of the top 10 sites contributed US$ 1.15 to the US$ 2 L^−1^ MDSP. The other US$ 0.85 of the *for-profit* model were policy and profit related costs.

*CapEx and Opex:* the CapEx for the 500 ha facility (Fig. 4c) ranged between US$ 128–245 Million (i.e., US$ 256,000–490,000 ha^−1^), with the three largest cost components being growth, HTL/refining and harvest/concentration systems. On a per hectare basis the sub-component CapEx cost of the algae production portion for the 500 ha facility (Darwin, Australia) was US$ 446,000 ha^−1^. This is ~ 15% below the actual construction cost of a 0.4 ha facility (US$ 525,000). This cost saving is in line with expected economies of scale achieved through the scale up from 0.4 to 500 ha (1250-fold scale up). The annual OpEx (Fig. 4d) ranged between US$ 7.7–16.4 Million (i.e., US$ 15,400–32,800 ha^−1^) with the three largest cost components being growth, harvest and utilities.

However, CapEx and OpEx alone were insufficient to predict profitability. For example, despite Darwin and Amsterdam having similarly high CapEx values (~ US$ 223 and US$ 245 Mil, respectively) and OpEx (US$ 13.7 Mil year^−1^ and US$ 12.6 Mil year^−1^), due to its operational conditions Darwin yielded an IRR of 13.2% (vs. Amsterdam –7%) and an MDSP of US$ 1.23 (vs. Amsterdam US$ 2.61) in the not-for-profit scenario. This highlights the importance of local climatic and operational conditions.

*Employment:* the facility will typically employ around 290 personnel during 2 years of design and construction and 74 personnel on a continuous basis during the 30-year operational life of the plant (based on Darwin, Australia).

Local policy settings also have a major impact. For example, a public utility approach can considerably reduce the price of fuel providing a key step on the path to a profitable commercial renewable fuel industry by attracting the required investment needed to advance technology and commercial biorefinery co-production options.

#### Policy effects

*Interest rates* were the second largest factor effecting financial viability. Their effect on the MDSP varied between 5% in Spain and 44% in Turkey (Fig. 4a). The 24% project finance rate prevailing in Turkey during 2018 when this data was assembled was the primary reason that this location did not return a positive IRR.

*Corporate tax* rates varied between 0% (Abu Dhabi) and 34% (Brazil) and the impact was proportional to the profitability at each location.

*Profit*: the most profitable location was Almeria primarily due to its very low discount rates and despite its modest 10.9% IRR. At the set US$ 2 MDSP price, most of the locations (Fig. 4a) demonstrated surprisingly similar inflation adjusted profits. Haikou, China had a comparatively low 9.5% IRR further reduced by the 6.5% discount rate. This lower IRR may stem from the particular climatic conditions in the selected location of Haikou, whereas other locations within China may deliver better results. The least profitable locations were Izmir (Turkey) and Amsterdam (Netherlands). While interest rates were the primary negative factor for Turkey, the Netherlands was affected by a combination of high labor and land costs and the lowest biomass productivity (8.6 g m^−2^ day^−1^). This information suggests that Turkey would be more attractive for renewable fuel production under conditions of reduced sovereign risk; Amsterdam appears better suited to the expansion of microalgae industries focused on higher value products.

The CO_2-eq_ emissions of microalgae diesel correspond to about one-third of non-renewable diesel based on the boundary conditions set in this study and so process profitability would benefit from carbon pricing. *Carbon pricing* was (2018) only applicable in 4 of the 12 locations, Amsterdam, Netherlands (US$ 8.20T^−1^ CO_2_ equivalent greenhouse gas emissions—CO_2_eq), (Almeria, Spain (US$ 8.20T^−1^ CO_2_eq), Chennai, India (US$ 5.85T^−1^ CO_2_eq) and Acapulco, Mexico (US$ 3.50T^−1^ CO_2_eq). Nevertheless, the locations governed by a carbon price, and the price itself are forecast to rise in the coming years. The Carbon Pricing Leadership Coalition forecasts that a carbon price of US$ 100T^−1^ by 2030 [45] will be needed as one of a series of measures to stay within a 2 °C rise in global temperatures. The effect of carbon pricing was, therefore, also analyzed at US$ 100T^−1^ for Almeria (Fig. 4a) to measure its effects at this forecast future price point. Under a US$ 100T^−1^ carbon price the profitability rose from 10.9 to 14.1%.

## Discussion

A rapidly expanding body of advanced climate [46] and global energy-use data [7] has firmly established the urgent need for strategic leadership and action on CO_2_ emissions reductions. Failure to deliver this is forecast to influence the future for centuries, if not millennia [46]. Despite significant advances in renewable stationary energy and electric vehicles, parallel development of renewable fuels (e.g., methane, ethanol, high-density liquid fuels and H_2_) is critical to meet international COP21 Paris CO_2_ emission reduction commitments and key UN Sustainable Development Goals (in particular SDG 7: affordable and clean energy and SDG 13: climate action, and others indirectly) [47]. Microalgae-based renewable fuel systems are a frontrunner option that can help to support this energy mix as they can supply high-density liquid fuels for aviation, shipping and long-haul transport using existing infrastructure with relatively low environmental impact. Moreover, the vulnerability of global supply chain disruptions revealed during the COVID-19 crisis underscores the importance of decentralized and distributed energy networks consistent with algae-based fuel production.

Prices of non-renewable diesel over the past 20 years have ranged between US$ 0.19–1.04 L^−1^. For the purposes of this study, we have set a benchmark target for microalgae diesel to achieve price parity, to US$ 0.80 L^−1^ (US$ 3.02 gal^−1^). It should, however, be noted that in 2019 the International Monetary Fund (IMF) concluded that fossil fuel subsidies, ‘*defined as fuel consumption times the gap between existing and efficient prices (i.e., prices warranted by supply costs, environmental costs, and revenue considerations), for 191 countries*” ranged between US$ 4.7 (2015)–5.2 trillion (2017), corresponding to 6.3–6.5% of annual GDP, respectively. Furthermore, the IMF concluded that “*Efficient fossil fuel pricing in 2015 would have lowered global carbon emissions by 28 percent and fossil fuel air pollution deaths by 46 percent, and increased government revenue by 3.8 percent of GDP*’ [48]. These factors are likely to exert an upward pressure on the price of traditional non-renewable diesel into the future. In contrast, technical and policy advances foresee a downward trajectory of microalgae renewable fuel price toward an intersect in the price of non-renewable diesel—especially if meaningful carbon pricing can be implemented.

In terms of technical advances, Fig. 2b, shows that the use of the dual microalgae strain approach, designed to optimize biomass productivity over the full 12-month period can increase the IRR in Abu Dhabi from 11.5 to 14.4%. Recent advanced, synthetic cell engineering technologies have potential to greatly improve algae traits for increased photosynthetic efficiency, biomass and lipid yields. For instance, ExxonMobil (EM) has partnered with Synthetic Genomics, Inc (SGI) with the aim to produce 10,000 barrels of algae fuels per day by 2025 [49]. Using synthetic biology techniques, EM-SGI researchers doubled lipid content of *Nannochloropis gaditana* by fine tuning a genetic switch that partitions carbon to oil, without compromising growth [50].

Automated pond construction techniques and process automation are likely to reduce CapEx and OpEx. Atmospheric CO_2_ capture [51], optimisation of light capture [40, 52, 53], production conditions [54, 55], strain selection [56] and breeding [57, 58] can also increase productivity. Improved biomass productivity can also reduce harvesting costs due to increased cell densities (Fig. 3). Bio-refinery concepts for the co-production of fuel and other higher value co-products can also improve profitability (see below).

Our analyses show that under a *for-profit business* model focused only on diesel production, 10 of the 12 locations achieved a minimum diesel selling price (MDSP) under US$ 1.85 L^−1^/US$ 6.99 gal^−1^ and nine under US$ 1.60 L^−1^ (US$ 6.04 gal^−1^). While encouraging, US$ 1.60 L^−1^ is still US$ 0.80 L^−1^ above the non-renewable diesel benchmark price of US$ 0.80 L^−1^. Increased international carbon pricing could reduce this gap but has proven difficult and consequently this study highlights an alternative path to competitive low CO_2_ emissions renewable fuel systems [29].

Under the *not-for-profit utility model,* eight locations achieved an MDSP of less than US$ 1.25 (US$ 4.73 gal^−1^). This price comparison can be extended to most other fuel types (e.g., jet fuel, petrol and bunker fuel), as the production and processing costs are similar on an energy content basis. The establishment of fuel utilities could, therefore, bring microalgae fuel prices to within US$ 0.45 L^−1^ of the US$ 0.80 non-renewable diesel bench mark price and less in an environment in which fossil fuel subsidies are reduced. Chennai actually returned an MDSP of US$ 1.15 reducing this gap to US$ 0.35 L^−1^. While a fuel price of US$ 1.15–1.25 is still US$ 0.35–0.45 L^−1^ above the US$ 0.80 non-renewable diesel benchmark, the introduction of co-product streams (e.g., protein, biopolymers and nanomaterials) could bridge this gap on the path to fully commercial biorefineries under future policy setting in which the carbon-price increases over time.

Microalgae-based fuels also offer local benefits through the provision of employment. For example, the Darwin (Australia) facility would employ 290 personnel during 2 years of design and construction and 74 personnel on a continuous basis during the 30-year operational life of a 500 ha plant. It would also support sustainable economic development which in turn can generate tax income [12]. Internationally, these technologies could provide a series of advantages which range from economic resilience and increased fuel, climate, political, social and environmental security enshrined in the UN Sustainable Development Goals (in particular *Affordable and Clean Energy* and *Climate Action).* Microalgae also provide mechanisms to contribute to circular economies and to support initiatives to keep these within our planetary boundaries.

## Conclusions

The CapEx for the twelve 500 ha facilities simulated (Fig. 4c) ranged between US$ 128–245 Million (i.e., US$ 256,000–490,000 ha^−1^) and the annual OpEx (Fig. 4d) between US$ 7.7–16.4 Million (i.e., US$ 15,400–32,800 ha^−1^). CapEx and OpEx alone were insufficient to predict profitability as climatic, operational and economic conditions had major impacts (Figs. 1, 3 and 4) highlighting the importance of conducting the location specific analyses presented. Under a *for-profit business* model focused only on diesel production, 10 of the 12 locations achieved a minimum diesel selling price (MDSP) under US$ 1.85 L^−1^/US$ 6.99 gal^−1,^ while using the *not-for-profit utility model,* eight locations achieved an MDSP of less than US$ 1.25 (US$ 4.73 gal^−1^). Moving forward, the judicious use of technology and policy optimisation could help to bridge the gap on the path to fully commercial biorefineries under future policy setting in which the carbon-price increases over time. The TELCA model can now be used to enable model guided systems design, assist with systems optimization, de-risk scale up and advance business models. The analysis presented also provides governments and other investors with a solid basis on which to assess whether they wish to encourage establishment of a microalgae industry in their jurisdiction, and if so, which technical advances and policy settings are likely to be most favorable. The analysis indicates that microalgae high-density renewable liquid fuels could be produced close to competitively in a broad range of countries (Graphical abstract and Fig. 4) and that price parity is likely achievable through the introduction of scaleable and higher value co-product streams (e.g., protein and biopolymers). As has been demonstrated in numerous other industries, early adopters are likely to be best positioned to establish the critical mass necessary to develop beneficial value chains, supply local markets and expand export opportunities.

## Supplementary Information

**Additional file 1.** Supplementary information detailing the presented TELCA simulation.

## Data Availability

The data sets used and/or analyzed during the current study are either reported in additional Data or available from the corresponding author on reasonable request.
