# Supplementary material for: Techno-economic evaluation of microalgae high-density liquid fuel production at 12 international locations
Source: Biotechnol Biofuels. 2021 Jun 7;14:133. doi: 10.1186/s13068-021-01972-4 (PMC8183327; doi:10.1186/s13068-021-01972-4)
Supplement: Supplementary file 1 — Additional file 1. Supplementary information detailing the presented TELCA simulation. [file 13068_2021_1972_MOESM1_ESM.docx]

**ADDITIONAL FILE MATERIAL**

**Techno-economic evaluation of microalgae high-density liquid fuel production at 12 international locations**

**Authors:** John Roles*^, Jennifer Yarnold*#^, Karen Hussey#, Ben Hankamer*

* The University of Queensland, Institute for Molecular Bioscience, 306 Carmody Road, Brisbane Qld 4072, Australia.

# The University of Queensland, Centre for Policy Futures, Faculty of Humanities and Social Sciences, Brisbane Qld 4072, Australia.

^ Joint first authors

**Contents**

1. **TELCA 2.0 Model**
   1. Location Specific and Productivity Input module
   2. Harvest Analysis module
   3. Project Finance Rate module
   4. Construction Labour Productivity module
   5. Model Structure upgrade from TELCA 1 to TELCA2
2. **Algae Productivity Model**
   1. **Light-temperature dependent growth model**
   2. **Temperature model**
      1. Governing Equations
      2. Solar Radiation
      3. Evaporation
      4. Thermal Radiation
      5. Conductive Heat Flux to Ground
   3. **Light Model**
      1. Modelling light-limited biomass productivity
      2. Calculation of local light intensities through the mass culture
   4. **Model Validation**
   5. **Simulated Temperature and Weather Profiles**
   6. **Simulated Productivity Data**
3. **TELCA 1.0 Model**
   1. **TELCA 1.0 Background**
   2. **TELCA 1.0 Process Modules**
   3. **TELCA 1.0 Module Descriptions**
4. **TELCA 1.0 Model Validation**

**Appendix 1**

**References**

**1. TELCA 2.0 Model**

The construction of the TELCA 1.0 model is fully described and validated in the Supplementary Methods S7 to S10. The TELCA 2.0 model has been further modified with the following additional modules that have been provided for calculation and control of:

- Location specific and Productivity inputs for each of the locations identified
- Harvest Analysis
- Project Finance Interest Rate Analysis
- Construction productivity Analysis

**1.1 Location Specific Environmental Input** **module**

For each of the locations analyzed a separate TELCA (Excel worksheet) module was created. The worksheet and content were referenced into the main inputs to TELCA 2.0 through a selection panel in the Harvest Analysis module, which connects with the high-rate pond module (see Fig.2, main paper).

**Description**

All location-specific user defined variables were documented in each location’s respective module including:

**Productivity tables** downloaded from the Algae Productivity Model (MATLAB) and compiled for: Algae Strain, Culture Depth, Concentration, Month of Year and Time of Day. These tables enable lookup functions to access productivity data based on specific hourly conditions prevailing for each month of the year.

**Monthly evaporation** data downloaded from the Algae Productivity Model (MATAB).

**Monthly rainfall** data established from official meteorological websites for each of the jurisdictions.

**Solar Irradiance** data from the RETScreen database at: <https://www.nrcan.gc.ca/energy/software-tools/7465>

**Base Labour Costs and Labour Non-Wage costs** were established from:

- 1. <https://www.payscale.com/research/AU/Job=Structural_Metal_Fabricator_%2F_Fitter/Hourly_Rate>
  2. <http://ec.europa.eu/eurostat/documents/2995521/7968159/3-06042017-AP-EN.pdf/6e303587-baf8-44ca-b4ef-7c891c3a7517>
  3. <https://tradingeconomics.com/labour-costs>
  4. <https://www.zu.ac.ae/main/en/research/publications/_documents/The%20Cost%20of%20Foreign%20Labor%20in%20the%20United%20Arab%20Emirates.pdf>
  5. <https://dlca.logcluster.org/display/public/DLCA/3.4+Tunisia+Manual+Labor+Costs;jsessionid=ADD0F82D45B5920A7C0D46961B8EE3EB>
  6. <https://knowledge.leglobal.org/working-conditions-in-india/>
  7. <https://knowledge.leglobal.org/working-conditions-in-brazil/>
  8. <http://ec.europa.eu/eurostat/documents/2995521/7968159/3-06042017-AP-EN.pdf/6e303587-baf8-44ca-b4ef-7c891c3a7517>
  9. <https://africapay.org/kenya/salary/minimum-wages/>
  10. <https://www.nordeatrade.com/no/explore-new-market/tunisia/work-conditions>
  11. <https://tradingeconomics.com/turkey/social-security-rate-for-companies>
  12. <http://www.invest.gov.tr/en-US/investmentguide/investorsguide/employeesandsocialsecurity/Pages/TermsOfEmployment.aspx>

**Currency Values** established on 30 June 2018 were from <https://www.xe.com/currencyconverter/>

**Steel Fabrication Costs** transferred from the Construction productivity and Steelwork Fabrication cost analysis module.

**Power supply costs** were established from:

- 1. [https://www.statista.com/statistics/596254/electricity-industry-price- xxxxx/](https://www.statista.com/statistics/596254/electricity-industry-price-%20xxxxx/)
  2. <https://www.addc.ae/en-US/business/Pages/RatesAndTariffs2017.aspx>
  3. <https://www.tangedco.gov.in/linkpdf/ONE_PAGE_STATEMENT.pdf>
  4. <https://stima.regulusweb.com/>
  5. <https://www.hawaiianelectric.com/billing-and-payment/rates-and-regulations/average-price-of-electricity>
  6. <https://energypedia.info/wiki/Tunisia_Energy_Situation>
  7. <http://ec.europa.eu/eurostat/statistics-explained/index.php?title=File:Electricity_prices_for_non-household_consumers,_second_half_2017_(EUR_per_kWh).png>

**Water supply costs** were established from Water pricing and sustainable surface irrigation management J.S. Sindhu Department of Economics, A.M. Jain College, Chennai-600 114, India

- 1. <http://deltaproof.stowa.nl/pdf/Pricing_of_water_for_agriculture?rId=72>
  2. <http://www.fao.org/docrep/008/y5690e/y5690e0b.htm>
  3. <http://www.fao.org/3/a-i5074e.pdf>
  4. <https://www.oecd.org/unitedstates/45016437.pdf>
  5. <https://www.addc.ae/en-US/business/Pages/RatesAndTariffs2017.aspx>
  6. <https://www.oecd.org/eu/45015101.pdf>
  7. <https://www.oecd.org/turkey/45016347.pdf>

**Land Prices** established from interpolation of unit rate area costs from rural and agricultural land real estate sales sites. Note that while the location referenced identifies a particular local town or city land prices were established for the lowest cost, topographically suitable land within a 100 km radius of the identified location.

While TELCA has the capacity to assess the impact of different locations based on average land slope this function was not utilized for site comparison purposes as it was assumed that in all cases land with less than 1:1000 slope would be selected.

**Interest Rates** were established from the Project finance interest rate analysis module.

**Inflation Rates and Company Tax** rates were established from <https://en.portal.santandertrade.com> and supplemented with additional national government data where specific details were unclear or insufficient.

**CO_2_ Sources.** While TELCA has the capacity to assess the impact of CO_2_ sourcing on both construction and operational costs this function was not utilized for comparison purposes. In all cases it was assumed that the facility would be constructed adjacent to an existing low pressure CO_2_ emitter such as a gas fired power station. The CapEx and OpEx associated with collection from such a source, cooling, filtering and distribution of CO_2_ were all included in the analysis.

**Nutrients.** TELCA provides for the implementation of a range of primary nutrient solutions. Previous analysis has indicated that primary supply of nitrogen through urea is the most cost-effective solution. Urea is an internationally traded commodity with price subject to world and sometimes local natural gas supply pricing. As such we have elected to not consider localized pricing for Urea or supporting nutrients and used undifferentiated prices from <http://www.amis-outlook.org/indicators/outside/fertilizer/en/> .

**Inputs**: All above listed items were direct user inputs which were tabulated.

**Outputs**: The tabulated data is a database that is called on by a number of other modules described below and in Roles^1^.

**1.2 Harvest Analysis module**

This module is designed to determine the relationship between *harvest rates*, *harvest times*, culture *concentration* and *productivity* in the open ponds. To minimize CapEx and OpEx it was assumed that all harvesting equipment was designed and selected on a single volume throughput basis. Variation of harvest could therefore be achieved by selecting the start and completion of harvest times applicable independently for each month of the year. The other available variable is the *nominal concentration* (nominal because concentration will vary throughout the day from differences between productivity/respiration and harvesting) for each month.

The module was also used to select target locations and the marshalling of location specific data.

**Description**

A user defines a target location. For this location the program imports the defined reference data set from the *Location Specific Environmental Input module.* Productivity data for the selected locations and depths are copied into tables in this module.

Based on user defined values for *harvest rate*, *harvest start times (monthly)*, *harvest duration (monthly)* and *base concentration (monthly)* an iterative analysis was performed in Excel to ensure the *biomass harvest volume* matched with the accumulative biomass for each day after accounting for productivity/respiration. Excel Solver was used to optimize the harvest rate and optimal base concentration values for either optimum IRR or optimum biomass productivity.

Annual average and peak concentrations and biomass volumes were then forwarded to other TELCA modules (e.g. the high rate pond module) for economic and process analysis.

**Inputs**

- Location specific productivity tables
- Location specific economic data
- Growth pond configuration data
- Overflow pond configuration data
- Fresh water top-up rate

**Outputs**

- Average harvest operational hours
- Harvest rate water volume
- Average biomass harvest rate
- Peak biomass harvest rate
- Fresh water top-up demand
- Salt water top-up demand
- Pond water discharge rate
- Target location specific economic and process variables

**User Defined Variables**

- Selected target location
- Pond Depth
- Harvest Rate
- Base concentration for each month

**1.3 Project Finance Rate module**

This module was designed to establish probable project finance cost applicable in each of the jurisdictions.

**Description**

Project finance costs are generally closely held commercial in confidence information that is unique to each project. They are held by finance providers and project proponents and are a culmination of risk factor analysis by the parties involved. To define applicable conditions for a mature, well established technological application in the renewable energy field were considered for the use of project finance rates. Industrial PV applications were considered a suitable basis for this analysis. To provide comparative finance costs across the many locations examined, a relationship between these project finance rates, and the respective government benchmark interest rates was established. Two points in this relationship were established: US applicable rates from NREL and rates applicable in Turkey from recent personal involvement of one of our staff members in the renewable energy project finance industry. From these points we established the following relationship:

PFR (%) = 1.142 x BIR(%) + 3.73%

PFR represents the Project Finance Rate

BIR represents the location specific government Benchmark Interest Rate applicable.

**Inputs**: Location specific benchmark interest rates

**Outputs**: Project finance rates

**1.4 Construction Labour Productivity module**

This module was created to establish construction labour productivity across each of the investigated locations.

**Description**

Labour productivity rates specific to the construction industry were unavailable for all except Eurozone countries. It was therefore necessary to establish a relationship between the published overall economy wide productivity data and data specific to the construction industry. The Eurozone data was used to establish this relationship. Wage adjusted productivity data from,

<https://www.ons.gov.uk/economy/economicoutputandproductivity/productivitymeasures/articles/internationalcomparisonsoflabourproductivitybyindustry/2014>

and economy wide GDP data from,

<https://data.worldbank.org/indicator/SL.GDP.PCAP.EM.KD?locations=BR&view=chart>

was normalised to known Australian construction industry labour productivity rates from the petroleum refinery construction industry to establish the following relationships:

CO = 0.2267 x GDPe + 24863

Where,

CO is the Construction Product Output per employee in USD

GDPe is the national GDP per employee in USD

and

PF = CO (Australia) / CO(target) x 1.27

Where

PF is the target country productivity factor used for modifying labour costs

**Inputs**: Location

**Outputs**: Labour productivity factor

**1.5 Model Structure upgrade from TELCA 1 to TELCA2**

Subsequent to the above TELCA validations, the following changes were made to the model to improve accuracy, performance and to bring the model into a configuration that provides greater siting flexibility:

**2015 price base**: For comparative purposes all costs in the model were adjusted from 2015 prices to 2011 prices (the basis for both the 2011 and 2014 NREL/PNNL/ANL analyses). The adjustment factor used for this in TELCA was 0.924, based on the US Federal Reserve Bank Economic Index for Construction and Machinery Prices. These price adjustments were removed and all prices reverted to a 2015 base. *The effect of this was an increase in MDSP of USD$0.25 L^-1^ or 8.5% to USD$3.18 L^-1^*.

**Salt water vs. fresh water**: Salt water systems were modelled in TELCA, resulting in higher construction material costs for structures and equipment in direct contact with the culture or downstream process flows. Most of the piping systems are provided in HDPE and so will be unaffected. Internals of pumps and valves required upgrading to marine grade stainless steel or other non-corroding materials. Similarly, the paddlewheels required a high level of surface treatment or fabrication from stainless steel, to facilitate a 30 year operational life. The estimated additional cost associated with these provisions was AUD$4.8M resulting in an increase in MDSP of USD$0.06 L^-1^. This cost increase, was offset by a saving in water supply costs of approximately USD$0.045 L^-1^ (NREL/PNNL/ANL figures), and environmental benefits. It was later shown, that the extra loss of nutrients due to salt water blowdown contributes about USD$0.04 L^-1^ of additional cost to the saltwater system. *The net effect was an MDSP increase of USD$0.05 L^-1^ or 1.5% to USD$3.23 L^-1^*.

**Electro-flocculation vs. chemical flocculation**: The capital costs associated with electro-flocculation are approximately A$5.4M higher than the equivalent cost for chemical dosing, the difference amounting to US$0.05 L^-1^ extra MDSP. Annual operating costs for the electro-flocculation plant including labour would be approximately AUD$1.1M delivering an implied MDSP contribution of USD$0.09 L^-1^ compared to the NREL/PNNL/ANL implied cost for chemical flocculation of USD$0.27 L^-1^. *The net effect was an MDSP reduction of USD$0.13 L^-1^ or 4.2% to USD$3.10 L^-1^.*

**Changed working capital allowance**: The working capital allowance was changed to reflect differing requirements for construction and operations. Working Capital of 5% of CapEx was retained for the construction period, as per NREL/PNNL/ANL but changed to 15% of OpEx for the operational period. *The effect was an MDSP reduction of USD$0.08 L^-1^ or 2.6% to USD$3.02 L^-1^.*

**Farm size increase:** Using the optimising analysis capability of TELCA the farm size was optimised to 570 ha instead of the NREL/PNNL/ANL size of 405 ha. The effect was an MDSP reduction of USD$0.08 L^-1^ or 2.6% to USD$2.94 L^-1^.

**Pond size optimised:** Using the system optimisation capabilities of TELCA the individual pond sizes were adjusted to 1050m x 45m. The effect was an MDSP reduction of USD$0.04 L^-1^ or 1.4% to USD$2.90 L^-1^.

**Changed Operating Days**: The annual operating regime was changed from the universal 330 days per year used by NREL/PNNL/ANL for a temperate climate in the southern United States to more appropriate values for subtropics and tropics of, 355 days for the operation of the growth ponds, and 345 days for operation of the processing operations. The 10 days of pond downtime each year are estimated as follows: This adjustment allowed for *Scheduled Pond emptying* 2 days x 50% productivity as algae continue to grow, *Schedule Pond cleaning* 2 days x 0% productivity, *Schedule Pond filling* 2 days x 50% productivity as algae continue to grow, Unscheduled outage 8 days at 50% productivity. TELCA allows the user to specify operational algae growth downtime as well as operational processing and refining downtime.

*The effect was an MDSP reduction of USD$0.08 L^-1^ or 2.7% to USD$2.82 L^-1^.* A portion of the downtime days used by NREL/PNNL/ANL for their 330 days regime would have probably been selected to coincide with winter low production periods although this was not specified. The productivity used here reflects the consistent figure of 14.7 g.m^2^.d^-1^ rather than a reduction that would probably result from this change. Increasing production from 330 to 355 days under a typical temperate annual productivity profile would be equivalent to increasing average productivity by 4.5% from 14.7 to 15.4 g.m^2^.d^-1^.

**Corporate tax rate decrease.** The corporate tax rate was reduced from the 35% rate prevailing in 2011 to the current US rate of 21%. *The effect was an MDSP reduction of USD$0.10 L^-1^ or 3.5% to USD$2.72 L^-1^.*

**2. Algae Productivity Model**

The Algae Productivity Model was developed in MATLAB. Actual hourly meteorological data over a typical year for each of the 12 locations was used as an input to the model for the following variables: solar radiation, air temperature, relative humidity, windspeed, and rainfall (Energy Plus, National Renewable Energy Laboratories. <https://energyplus.net/weather>).

**2.1 Modelling light-limited biomass productivity**

During the growth phase, the volumetric biomass productivity of the system, *P_vol_* (reported as kg.m^-3^) is determined by the rate of change of the algal biomass concentration, *C_x_* over time is:

**Eq. 2.1** $P_{vol(t)}= \frac{dC_{x}}{dt}=\bar{\mu}C_{x}-RC_{x}$

Where $\bar{\mu}$ and R are the mean specific growth and respiration rates at a given time, *t*, (accounting for cell death, which is factored from empirical parameter values), integrated at all points, *z* over the reactor depth, *d* (and for stacked FPRs at all points, *y*, over the reactor height, *h*,

**Eq. 2.2**  $\bar{\mu}\left( t \right)=\frac{1}{h\cdot d}\int_{0}^{h} \int_{0}^{d} \mu\left( y,z,t \right)C_{x}dzdy$

Eq. 2.1 and 2.2 are valid for cultivation under batch mode and continuous mode, however for the latter, the dilution rate, *D* = $\bar{\mu}$ and *C_x_* will remain at a pseudo steady state.

The areal productivity at a given time, *P_areal_* is,

**Eq. 2.3** $P_{areal(t)}= \frac{P_{vol(t)}*{PBR}_{vol}}{{PBR}_{area}}$

Where *PBR_vol_* is the volume of the open pond *PBR_area_* is the system’s areal footprint, including the area of the system and spacing distances between adjacent reactors.

The specific growth rate, *µ* (h^-1^) is estimated based on the simple model of Bernard and Remond (2012) ^2^which estimates growth rate as a function of irradiance and temperature,

**Eq. 2.4** $\mu\left( T,I \right)=\mu_{max}\frac{I_{loc}}{\frac{\mu_{max}}{\sigma}\left( \frac{I_{loc}}{I_{opt}}-1 \right)^{2}} \Phi(T)$

In Eq 2.4, *µ_max_* is the maximum growth rate of a given species (day^-1^); the light response parameters σ and *I_opt_* define the half saturation rate of photosynthesis (µmol m^-2^ s^-1^) and the irradiance at which maximum growth occurs; and *Φ* is the proportional effect of temperature, using the inflexion function of Rosso et al^3^,

**Eq. 2.5** $\Phi\left( T \right)= \frac{\left( T- T_{max} \right) \left( T- T_{min} \right)^{2}}{\left( T_{opt}- T_{min} \right) \left[ \left( T_{opt}- T_{min} \right)\left( T- T_{opt} \right)-\left( T_{opt} - T_{max} \right)\left( T_{opt} + T_{min}-2T \right) \right]}$

In Eq. 2.5, the parameters Topt, Tmin and Tmax represent three cardinal temperatures of biological significance, these being respectively, the optimal temperature at which growth is highest at a given irradiance, and the minimum and maximum temperatures which define the threshold beyond which no growth occurs (Eq.4),

**Eq. 2.6** $\mu_{max}= \left\{ \begin{aligned} 0 for T<T_{min} \\ \mu_{max}\cdot\Phi\left( T \right) for T_{min}<T< T_{max} \\ 0 for T>T_{max} \end{aligned} \right.$

**2.2 Temperature Model**

To model the dynamic temperature changes of the culture broth in a microalgae open pond reactor, a model was developed using ODE15s to solve for the broth temperature over time in MATLAB (MathWorks). This model is based on a heat balance of the most relevant heat fluxes affecting the reactor as shown in Figure S1, based on the thermal model described by Bechet et al. 2010^4^. Key assumptions and parameter values used for validation are reported in Tables S3 and S4.


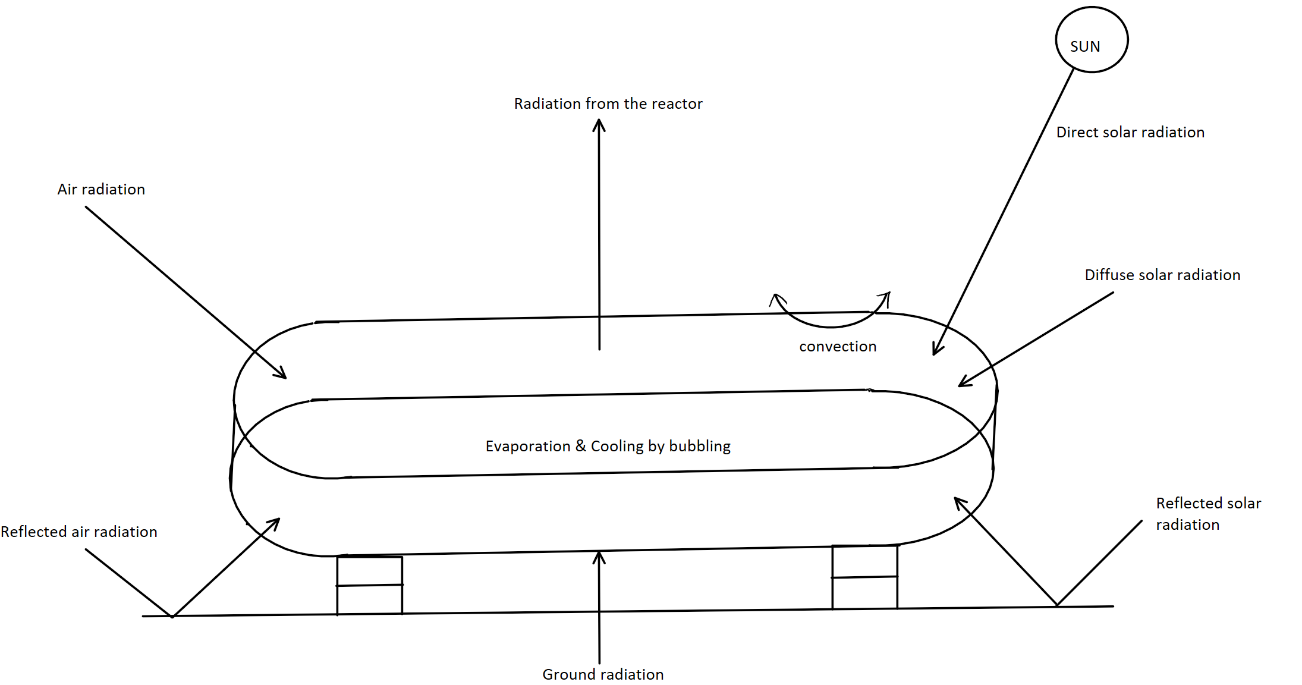


**Figure S1** Schematic of different sources of heat flux affecting the temperature of microalgae cultures.
Figure by Huda Rao, 2018.

The different heat fluxes that account for the most significant variations are solar radiation, *Q_sol_*, evaporation, *Q_ev_*, thermal radiation at the pond surface between the air and the water, *Q_ther_* and conduction to the soil, *Q_cond_*.

The model is an overall energy balance defined by *Q*, such that the change in temperature of the liquid media is defined as:

**Eq. 2.7**  $\frac{dT}{dt}V\rho c_{p}=Q_{solar}+Q_{evaporation} +Q_{thermal} +Q_{conduction}$

**S2.2.1 Solar Radiation (Q_solar_)**

In Eq. 2.7, the solar component is further divided into direct beam radiation (unscattered), *Q_sol,B_* and diffuse, Q*_sol,D_* radiation.

The proportion of direct solar radiation that is either transmitted and absorbed or reflected from the pond are dependent on the angle of radiation to the surface normal,

**Eq. 2.8**  $Q_{solar}={(Q}_{sol,B}+ Q_{sol,D}){\tau\varepsilon}_{1}$

where $\varepsilon$

ε is the emissivity of water (0.97) τ is the transmittance,

**Eq. 2.9**

$$=1-(r_{\perp}+r_{\parallel})$$

and *r*$\perp$ and *r*$\parallel$ are the proportions of reflected radiation,

**Eq. 2.10, 2.11**  $r_{\perp}=\frac{{{sin}^{2}(\theta}_{2}- \theta_{1})}{{{sin}^{2}(\theta}_{2}+ \theta_{1})}$ and $r_{\parallel}= \frac{{{tan}^{2}(\theta}_{2}- \theta_{1})}{{{tan}^{2}(\theta}_{2}+ \theta_{1})}$

where the refractive angle, θ_2_, is defined by Snell’s law,

**Eq. 2.12**  $\theta_{2}={sin}^{-1}\left[ \frac{n_{1}{sin\theta}_{1}}{n_{2}} \right]$

θ_2_ is the refracted angle after passing from one from one medium with a refractive index, n_1_ (air) to another medium with a second refractive index, n_2_ (water), where *ϴ_1_* is the angle of incident direct radiation. For a horizontal surface such as an open pond, this angle is the zenith angle, θ_z_

**Eq. 2.13**  $\theta_{z} ={cos}^{-1}(cos(\delta)\cdot cos(\varphi)\cdot cos(\omega)+sin(\delta)\cdot sin(\varphi))$

For diffuse radiation, we consider it to be isotropic (equal from all angles). Therefore, the fractions of transmitted and reflected light are found using the following equation by substituting θ with a fixed angle of 60^o^ (Duffie and Beckman^5^).

**2.2.2 Evaporation (Q_evaporation_)**

Various models have been proposed to estimate evaporation losses from reservoirs and lakes. The pan evaporation calculated can be converted into reservoir evaporation by multiplying with standard pan coefficient. The accepted standard coefficient for a 1.22m (4’) diameter US Class A land pan is 0.70. http://cwc.gov.in/main/downloads/Evaporation%20Control%20in%20reservoirs.pdf

The salt content in water affects the rate of evaporation. Experimental studies show that the rate of evaporation decreases with increase in salt content in water. In the case of sea water, the evaporation is 2 to 3% less as compared to fresh water, when other conditions are same.

Kohler^6^ and others have calculated evaporation from lakes by converting measured evaporation from pan to lake evaporation by applying a coefficient. Blaney^7^ studied the effects of high altitude on evaporation from pans and determined suitable coefficients. Studies by Bigelow^8^ showed that the location of pans relative to the water of a reservoir has significant effect on the calculated evaporation and concluded that evaporation from natural lakes or reservoirs is about 5/8 as fast from an isolated pan placed outside the vapour blanket. Further studies by Rohwer^9^, Kohler^6^, and Mansfield^10^ showed that the evaporation coefficient ranges anywhere between 0.2 to 1.5 and this factor is dependent upon size, depth and location. With this kind of evaporation measurement, it is essential that the coefficient of evaporation be measured under all different conditions, which is not practically feasible in large water storage systems.

http://cwc.gov.in/main/downloads/Evaporation%20Control%20in%20reservoirs.pdf

Reca et al.^11^ - Recently, research works on evaporation losses in reservoirs in semiarid regions have been conducted (Martínez-Álvarez^12^; Gallego-Elvira^13^). Other researchers performed studies to measure and analyse the evaporation in different types of waterbodies of varying size (Assouline^14^) and water depths (Assouline^15^). In these studies, significant advances were made in understanding the evaporation process and the measurement and modelling of water losses. Despite these findings, it is currently not common for them to be put into practice for the optimal management and operation of agricultural reservoirs.

**Eq. 2.14**  $Q_{evaporation}= h_{we} g_{s} A$

where *h_we_* is the evaporation heat of water (~2.45e^6^ J s^-1^), *A* is the surface area of the pond (m^2^) and *g_s_* is the amount of evaporated water (mm m^-2^ s^-1^) is found by,

**Eq. 2.15**  $g_{s}= \omega(x_{s}-x)/3600$

here, *ω* is the evaporation coefficient (kg m^-2^ h^-1^),

**Eq. 2.16**  $\omega=(25+19v)$

and *v* is the wind velocity above the water surface (m s^-1^).

*X_s_* is the maximum humidity ratio of saturated air at the same temperature of the water surface (kg kg^-1^) and *x* is the humidity ratio of air (kg kg^-1^).

Using tables relating to these values (Engineering Toolbox), we have established the following polynomial relations,

**Eq. 2.17**  $x_{s}=2.15e^{-5}T_{r}^{2}+1.12e^{-4}T_{r}+4.05e^{-3}$

where *T_pond_* is the temperature of the reactor media, and

**Eq. 2.18**  $x=exp\left( 0.06469 T+ln\left( 3.74e^{-5}RH+3.17e^{-5} \right) \right)$

where *T_air_* is air temp (^o^C) and RH is relative humidity (%).

**2.2.3 Thermal radiation (*Q_thermal_*)**

Thermal radiant heat transfer at the pond surface is defined as,

**Eq. 2.19**  ${(T}_{air}\varepsilon_{2}-T_{pond}\varepsilon_{1}){}^{4}$

where σ is the Stefan-Boltzmann constant (W m^-2^ K^-4^) and ε_2_ is emissivity of air.

**2.2.4 Conductive heat flux to the ground (*Q_conduction_*)**

Some heat is exchanged between the sides and the base of the pond to the ground below,

**Eq. 2.20**  $Q_{conduction}= k_{w}\frac{(T_{ground}-T_{pond})}{l_{w}}$

where *k_w_* is the thermal conductivity of the material (HDPE, W m-2 K-1), *l_w_* is the thickness of the wall (m).

The mean ground temperature is a function of the depth and can be quite complex. For simplicity, we have estimated it as follows,

**Eq. 2.21**  $T_{ground}= \bar{T}_{air}-T_{air(max)} exp(z/z_{o})cos(\left( \frac{360}{365} d- d_{air(\min)}-z/z_{o} \right)\frac{\pi}{180} )$

where $\bar{T}_{air}$ is the annual mean air temperature, *T_air(max)_* is the maximum daily temperature, d is the day and *d_air(min)_* is the day of the minimum temperature, *z* is the depth (m) and *z_0_* is found,

**Eq. 2.22**  $z_{o}=\sqrt[2]{\frac{2 d_{h}}{2 \pi}}$

where *d_h_* is the thermal diffusivity of soil (m^2^ s^-1^)

**2.3 Light Transfer Model**

**2.3.1 Calculation of local light intensities through the mass culture**

Irradiance through a culture with microorganisms is highly variable due to absorption and scattering of the cells in the medium. The local PAR-averaged PPFR, *I_loc_(z,ŝ)* at a given location, *z*, travelling along a particular path, *ŝ*, through a suspension of microorganism can be found by solving the radiative transfer equation. The total irradiance is the sum of the direct and diffuse components.^16^

For direct beam (collimated), *I_B,λ_(z,ŝ)*, the RTE is defined as follows:

**Eq. 2.23** $\frac{\partial I_{B,\lambda}(z,ŝ)}{\partial z}=-\beta_{eff,\lambda}I_{B,\lambda}(z,ŝ)$

While the steady state radiative transfer for diffuse, *I_D,λ_(z,ŝ)* light is found by:

**Eq. 2.24** $\frac{\partial I_{D,\lambda}\left( z,ŝ \right)}{\partial z}=-\beta_{eff,\lambda}I_{d,\lambda}\left( z,ŝ \right)+ \frac{\sigma_{eff,\lambda}}{4\pi} \int_{4\pi} I_{d,\lambda}\left( z,ŝ \right)\Phi_{\lambda}\left( ŝ_{i},ŝ \right)d\Omega_{i}$

$$+ \frac{\sigma_{eff,\lambda}}{4\pi} \int_{4\pi} I_{c,\lambda}\left( z,ŝ \right)\Phi_{\lambda}\left( ŝ_{i},ŝ \right)d\Omega_{i}$$

In Eq. 2.23 and 2.24, *β_eff,λ_*, is the extinction coefficient expressed as,

**Eq. 2.25** $\beta_{eff,\lambda}=\kappa_{eff,\lambda}+ \sigma_{eff,\lambda}$

Here, *κ_eff,λ_* and *σ_eff,λ_* are the spectral absorption and scattering coefficients of the microorganism respectively.

The scattering phase function of the microorganism, *Φ_λ_*, represents the probability that light travelling in the solid angle, *dΩi* around the direction *ŝ_i_* will be scattered into the solid angle *dΩ* around the direction ŝ. The first integral term corresponds to the in-scattered diffuse radiation and the second accounts for the in-scattered collimated radiation.

Several solutions of the RTE equation exist of varying complexity^17-19^. The most simple is the often used Beer-Lambert law,

**Eq. 2.26**  $I_{loc,\lambda}(z)=I\cdot exp(-E_{ext,\lambda} z)$

where *E_ext,λ_*, is the spectral mass extinction coefficient expressed as,

**Eq. 2.27**  $E_{ext,\lambda}=E_{a,\lambda}+ E_{s,\lambda}$

and *E_a,λ_* and *E_s,λ_* are the spectral mass absorption and scattering coefficients of the microorganism respectively.

Where *ϴ* is the angle of the direct beam radiation, *z* is the local position, *L* is the reactor depth, and the two flux extinction coefficients, *δ*_dir_ and *δ*_dif,_ for direct and diffuse respectively are,

**Eq. 2.28 and 2.29** $\delta_{dir}=\frac{\alpha C_{x}}{\cos\theta} (E_{a}+2bE_{s})$, and $\delta_{dif}=2\alpha_{1}C_{x} (E_{a}+2bE_{s})$,

In Eq. 2.28 and 2.29, *E_a_* and *E_s_* are the PAR-averaged mass absorption and scattering coefficients, *b* is the backward scattering fraction, and *α,* is the linear scattering modulus (Pruvost^20^),

**Eq. 2.30** $\alpha=\sqrt{\frac{E_{a}}{(E_{a}+2bE_{s})}}$

For open ponds with a non-reflecting, opaque base, total local irradiance is found by summing the direct and diffuse components:

**Eq. 2.31** $I_{loc}\left( z \right)=I_{loc,B}\left( z \right)+ I_{loc,D}(z)$

**Table S1. Input values and decision variables used for Algae Production Model simulations analysis.**

| **Description** | **Open Ponds** |
| --- | --- |
| ***Fixed parameters used for simulations*** |  |
| Time interval for hours in day, *ħ* | 1 |
| Time interval for days in year, *N* | 1:365 |
| Pond width (m) | 20 |
| Pond length (m) | 50 |
| Illuminated surface area (m^2^.reactor^-1^) | 820 |
| Harvest method | Continuous |
| ***Adjusted variables used for simulations*** |  |
| Cultivation location (latitude), *φ* | 12 locations |
| Spacing distance (m) | n/a |
| Spacing distance : reactor height ratio | n/a |
| Areal illuminated surface area, *SA_areal_* (m^2^.m^-2^ footprint) | 0.82 |
| No. of reactors (hectare^-1^) | 10 |
| Inoculating concentration, *C_x_* (g L^-1^) | 0.05–1 |
| Reactor depth, *L* (m) | 0.1–0.3 |
| Culture optical thickness (*C_x_L)* | 0.005 – 0.3 |
| Reactor volume (m^3^.reactor^-1^) | 205 |

**Table S2. Algal species-specific model parameters used for productivity modelling.**

|  | *D. tertiolecta* | *N. oceanica* |  |  |
| --- | --- | --- | --- | --- |
| *T_min_* | 5 | -0.2 | Min temp, ^o^C ^2^ | |
| *T_opt_* | 32.6 | 26.7 | Optimum temp (max growth), ^o^C ^2^ | |
| *T_max_* | 38.9 | 33.3 | Max temp, ^o^C ^2^ | |
| *µ_max_* | 3.35 | 3.5 | max growth rate, day^-1^ (mean from Bernard 2012)^2^ | |
| *K_s_* | 58 | 29 | Half saturation irradiance, μmol m^-2^ s^-1^ ^21^ | |
| *I_opt_* | 275 | 203 | Optimum irradiance, μmol m^-2^ s^-1^ ^21^ | |
| *E_a_* | 141 | 178 | Mass absorption coefficient, m^2^ kg^-1^ ^22^ | |
| *R_b_* | 0.2 | 0.2 | Basal respiration rate, d^-1^ ^23^ | |

**Table S3. Key assumptions of heat flux model.**

| **Assumptions** | **Justifications** |
| --- | --- |
| A1: Dynamic system | Since the reactor temperature changes over time, it is therefore in an unsteady state.  dTRdt≠0, dTgdt≠0 |
| A2: Lumped parameter system – perfect mixing | A paddlewheel and CO2 bubbling promoted mixing inside the reactor. There is a uniform temperature distribution at one point in time inside the reactor. |
| A3: Negligible kinetic and potential energy | There are no movement of equipment involved. The kinetic movement of the paddlewheel is considered constant over time. |
| A4: Negligible evaporative mass loss | The mass evaporation of the reactor will be neglected since the reactor volume is in large scale. No evaporation occurs at the top surface of the liquid assuming the air leaving the reactor is saturated in water and at the reactor temperature. |
| A5: Negligible conductive cooling | The reactor is raised from the ground using blocks placed on the corners. The contact surface area is small and thus conduction from the bricks is considered negligible. |
| A6: Negligible internal convection cooling | The reactor temperature is considered uniform and thus inlet and exit temperatures are equal. |
| A7: Radius of 0.65m | The arced reactor ends are treated as a circle with 0.65m radius. |
| A8: Uniform reactor surface temperature equal to liquid temperature | The resistance to heat transfer between the liquid and reactor walls is considered negligible. |
| A9: The temperature of air in headspace is assumed to be equal to temperature of broth | The volume of air in the headspace is small (need to quantify), and the convection between air and liquid broth at top surface is considered negligible. |
| A10: The fraction of solar radiation used by algae to carry out photosynthesis is neglected in the heat balance | Low fraction <4% ^4^ of total solar radiation used by algae (need to quantify for KBR ponds). |
| A11: Absorptivity = emissivity for ground surface, reactor walls and bottom surface (trough) and solution inside reactor. | These surfaces are considered opaque gray diffuse bodies since they are non-transparent and their radiation properties are independent of wavelength. |
| A12: Cooling by air bubbling can be expressed as eq. 20 | Air bubbles reach thermal equilibrium with liquid inside the reactor and at the top surface ^4^ |
| A13: Initial ground temperature is equal to the of average air temperature | The ground surface is assumed to be thin and at uniform temperature ^4^ |
| A14: The solution properties were assumed to be equal to that of water. | Algae dry weight and nutrient concentrations are low.  ^4^ |

| **Table S4: Problem data (parameters and constants) for temperature model validation data.** | | | | |
| --- | --- | --- | --- | --- |
| **Variable** | Definition | Value | Unit | References |
| **Reactor** | | | | |
| **Rl** | Reactor length | 6.5 | m | - |
| **Rw** | Reactor width | 1.12 | m | - |
| **Rh** | Reactor height | 0.3 | m | - |
| **SAt** | Surface area of top and bottom | 14.3 | m^2^ | Calculated based on geometry |
| **SAs** | Surface area of sides | 4.34 | m^2^ | Calculated based on geometry |
| **_Vr_** | Volume of reactor | 2.15 | m^3^ | Calculated based on geometry |
| **_Fb_** | Bubble flow rate | 0.00002 | m^3^/s | Estimate based on Béchet et al., 2010 air flow rate |
| **1** | Transmittance of white trough (opaque) | 0 | - | Opaque materials do not transmit radiation  Çengel and Ghajar, 2015 (p. 737)^24^ |
| **r** | Emissivity of reactor | 0.97 | - | Béchet et al., 2010^4^ |
| **Fs** | Form factor lateral | 0.5 | - | Reactor walls ‘see’ half sky and half ground |
| **Fbot** | Form factor bottom | 1 | - | Reactor bottom ‘sees’ all of ground |
| **Fw** | Velocity of water flow | 0.15 | m/s | Based on experimental data |
| Water | | | | |
| **w** | Density of water ground | 997 | kg/m^3^ | Engineering Toolbox (www.engineeringtoolbox.com) |
| **Cpw** | Heat capacity water | 4180 | J/kg.K | Engineering Toolbox |
| **Lw** | Latent heat of water | 2450000 | J/kg | Engineering toolbox |
| **r** | Dynamic viscosity of water | Calculated using equation for liquids | kg/m.s | (www-mdp.eng.cam.ac.uk, 2018) |
| **Ground** | | | | |
| **g** | Density of dense concrete | 4000 | kg/m^3^ | Béchet et al., 2010^4^ |
| **g** | Emissivity of ground | 0.88 | - | Çengel and Ghajar, 2015 (p.745)^24^ |
| **Cpg** | Heat capacity of ground | 2400 | J/kg.K | Béchet et al., 2010^4^ |
| **g** | Ground (concrete) thermal conductivity | 1.4 | W/.m.K | Çengel and Ghajar, 2015 (p. 919)^24^ |
| **Tgref** | Reference ground temperature | 293 | K | Béchet et al., 2010 Supporting info (SI-8) Average air temperature^4^ |
| **Fg** | Characteristic value for flux reaching ground surface | 300 | W/m^2^ | Béchet et al., 2010 (supporting info SI-8). Value can be calculated. Model is unsensitive to this value.^4^ |

| **Air** | | | | |
| --- | --- | --- | --- | --- |
| **a** | Density of air | 1.185 | kg/m^3^ | Engineering Toolbox |
| **Cpa** | Heat capacity of air | 1005 | J/kg.K | Engineering Toolbox |
| **a** | Emissivity of air | 1 | - | Béchet et al., 2010^4^ |
| **Pra** | Prandtl number air | 0.71 | - | Engineering toolbox |
| **va** | Kinematic viscosity | Calculated (linear equation) | m^2^/s | Engineering toolbox |
| **aa** | Air diffusivity | Calculated (linear equation) | m^2^/s | Engineering toolbox |
| **a** | Air thermal conductivity | Calculated (linear equation) | W/m.K | Engineering toolbox |
| **a** | Dynamic viscosity of air | Calculated using equation for gases | kg/m.s | (Www-mdp.eng.cam.ac.uk, 2018) |
| **Xa** | Concentration of water in air | 1.8 | kg/m3 | Jen’s value |
| **Input variables** | | | | |
| **z** | Zenith angle | Varies dynamically with model | Radians | Weather station data |
| vw | Wind speed | Varies dynamically with model | m/s | Weather station data |
| gr | Global irradiance | Varies dynamically with model | W/m^2^ | Weather station data |
| **Idiff** | Diffuse irradiance | Varies dynamically with model | W/m^2^ | Weather station data |
| **Idir** | Direct irradiance | Varies dynamically with model | W/m^2^ | Weather station data |
| **Ta** | Air temperature | Varies dynamically with model | K | Weather station data |
| _RH_ | Relative humidity | Varies dynamically with model | - | Weather station data |
| **Universal constants** | | | | |
| g | Gravity acceleration | 9.81 | m/s^2^ | Engineering toolbox |
| **Rec** | Critical Reynolds number | 50000 | - | Çengel and Ghajar, 2015 (p. 392)^24^ |
|  | Stefan Boltzmann’s constant | 5.67x10^-8^ | W/m^2^.K^4^ | Engineering toolbox |
| Note: for simplicity λ,μ,a and v do not need to be calculated using linear equation and can be assumed at a constant value under the assumption that water and air have constant physio-chemical properties. | | | | |

**2.4 Model Validation**

A numerical solution of a temperature model was validated against experimental data of an outdoor raceway pond and ground (Figure 2). The reactor geometry is rectangular with half-circular ends on both ends of the reactor. A schematic representation of the reactor can be seen in **Figure S3**. The desired accuracy should be +/-15% of the real process. The presence of a paddle wheel and air stone bubbler are assumed to provide a quasi-uniform temperature distribution and microalgae composition/ concentration profile inside the reactor. Therefore, the reactor tank is expected to have uniform temperature distribution at any point inside the solution in the tank.

The values used for productivity modelling were validated against Bernard and Redmond^2^. For light modelling, the values for spectral irradiance at depth are validated by Lee, et al.

^25^ . The model to predict changes in media temperature was validated with data collected from two 2000 L ponds at the Centre for Solar Biotechnology Pilot plant, Brisbane. Figure S1, shows a very tight fit between the measured and predicted media temperature found for both ponds over a 6 day period with an R2 value of 0.9 and 0.91 for ponds 7 and 8 respectively.

**
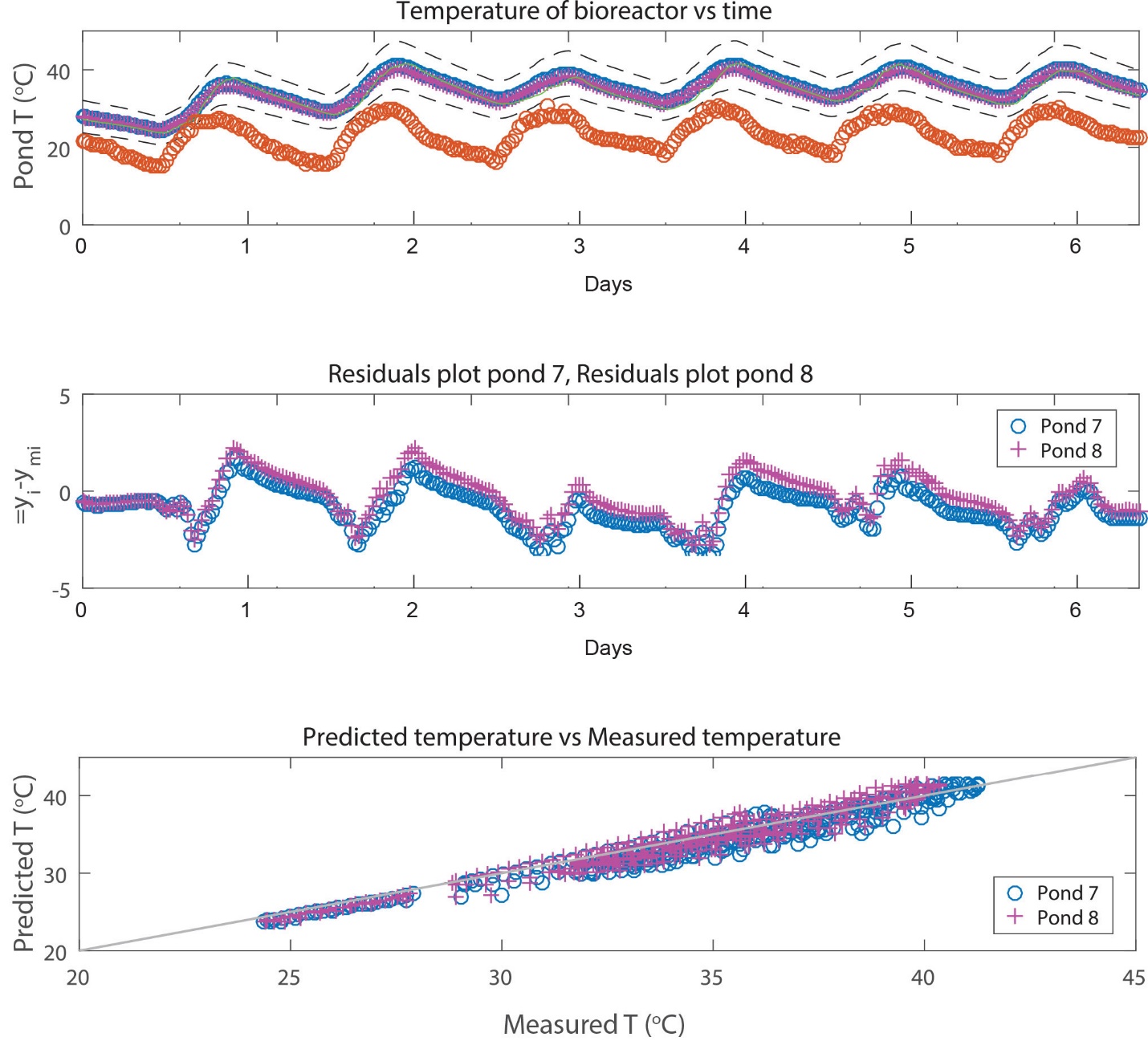
**

**Figure S2:** Comparison of modelled temperature profiles with experimental profiles in two 2000L ponds (i.e. Ponds 7, blue circles and 8, purple crosses) located in Brisbane, over a 6 day period. Top: model predicted culture broth temperature (grey line) and 95% confidence interval (dashed lines) in comparison to measured broth temperature for two 2000 L closed ponds (blue and purple markers). Measured air temperature (orange circles) was one input variable used in the model and is shown for comparison. Middle: residual plot showing the variation between measured and actual data (oC). Bottom: fitting of measured versus predicted culture broth temperature has a high correlation.


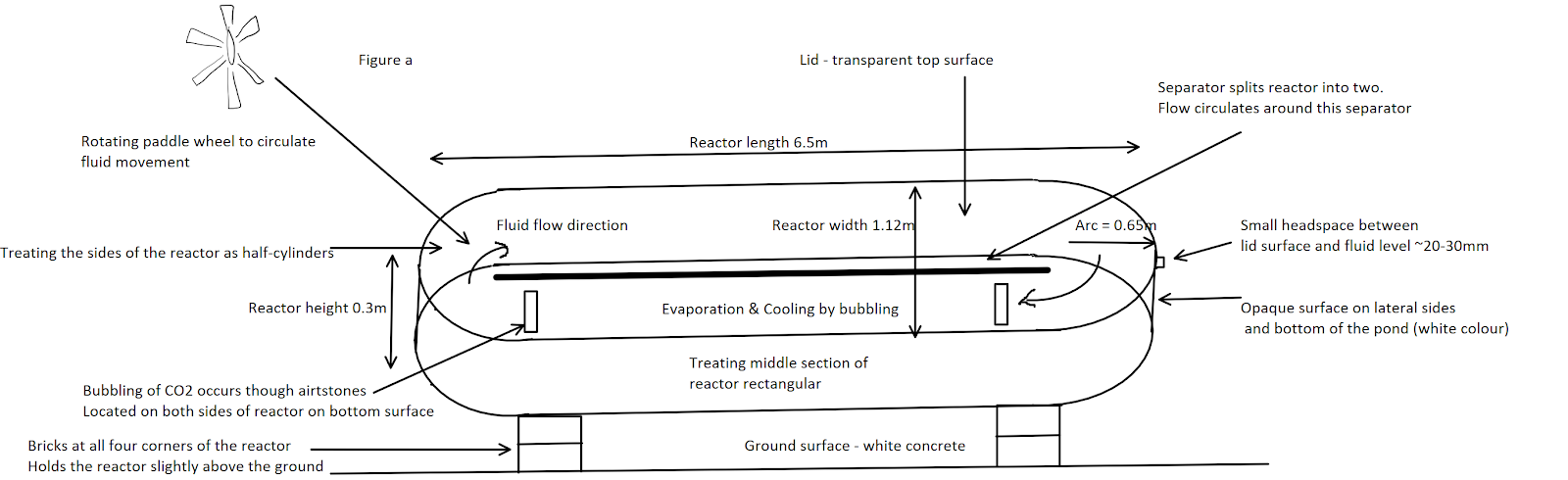


**Figure S3.** Schematic of open raceway pond system used to obtain experimental data for validation against simulations. Pond dimensions are listed in Table 3.

**Table S5.** Statistical results of actual vs modelled pond temperature data for two ponds (7 and 8).

| Mean residuals pond 7 | -0.86 | Mean residuals pond 8 | -0.3 |
| --- | --- | --- | --- |
| Normalised RMSE pond 7 | 0.08 | Normalised RMSE pond 8 | 0.07 |
| Relative RMSE pond 7 | 0.04 | Relative RMSE pond 8 | 0.03 |
| R-squared pond 7 | 0.9 | R-squared pond 8 | 0.91 |
| Sum of square of residuals pond 7 | 531.58 | Sum of square of residuals pond 8 | 398.55 |
| Total sum of squares pond 7 | 5133.28 | Total sum of squares pond 8 | 4600.32 |

**2.5 Temperature and weather profiles by location**


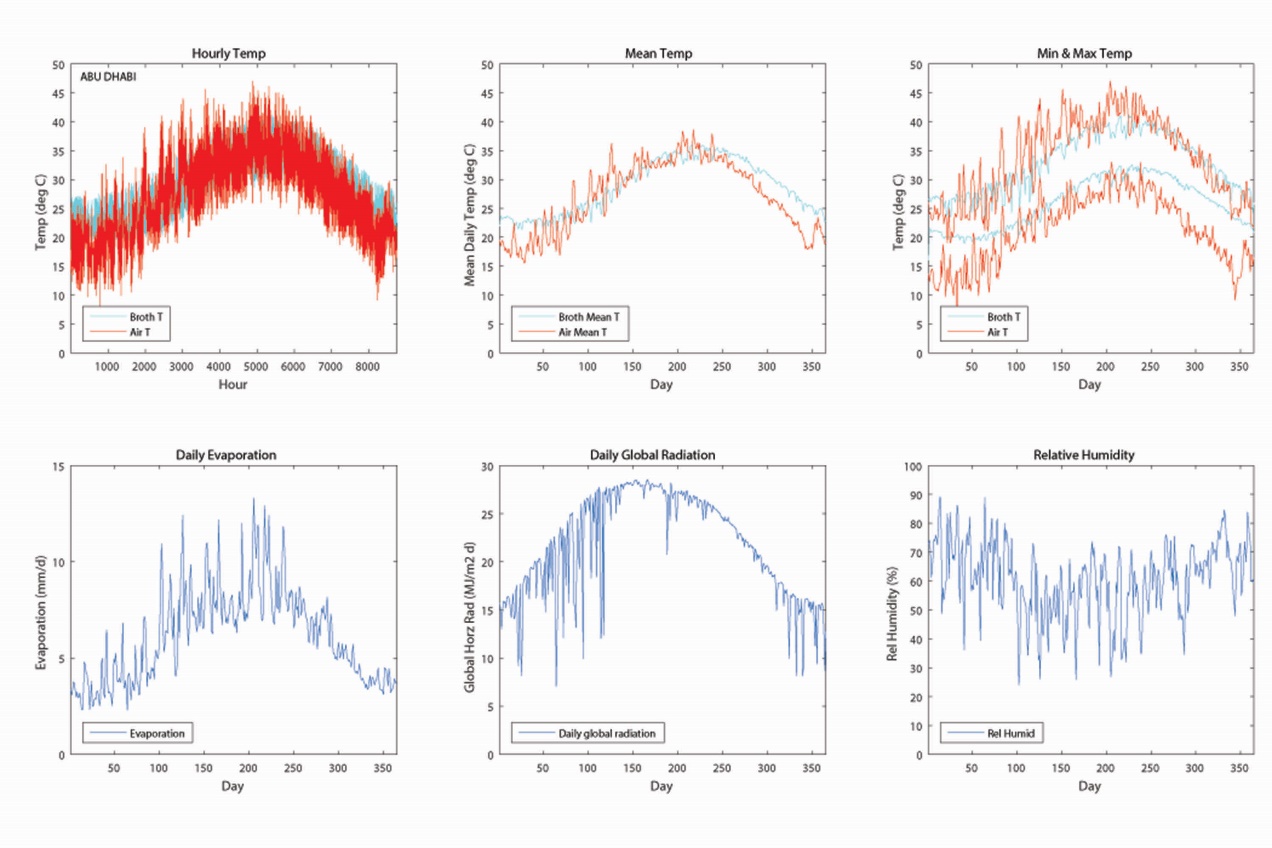

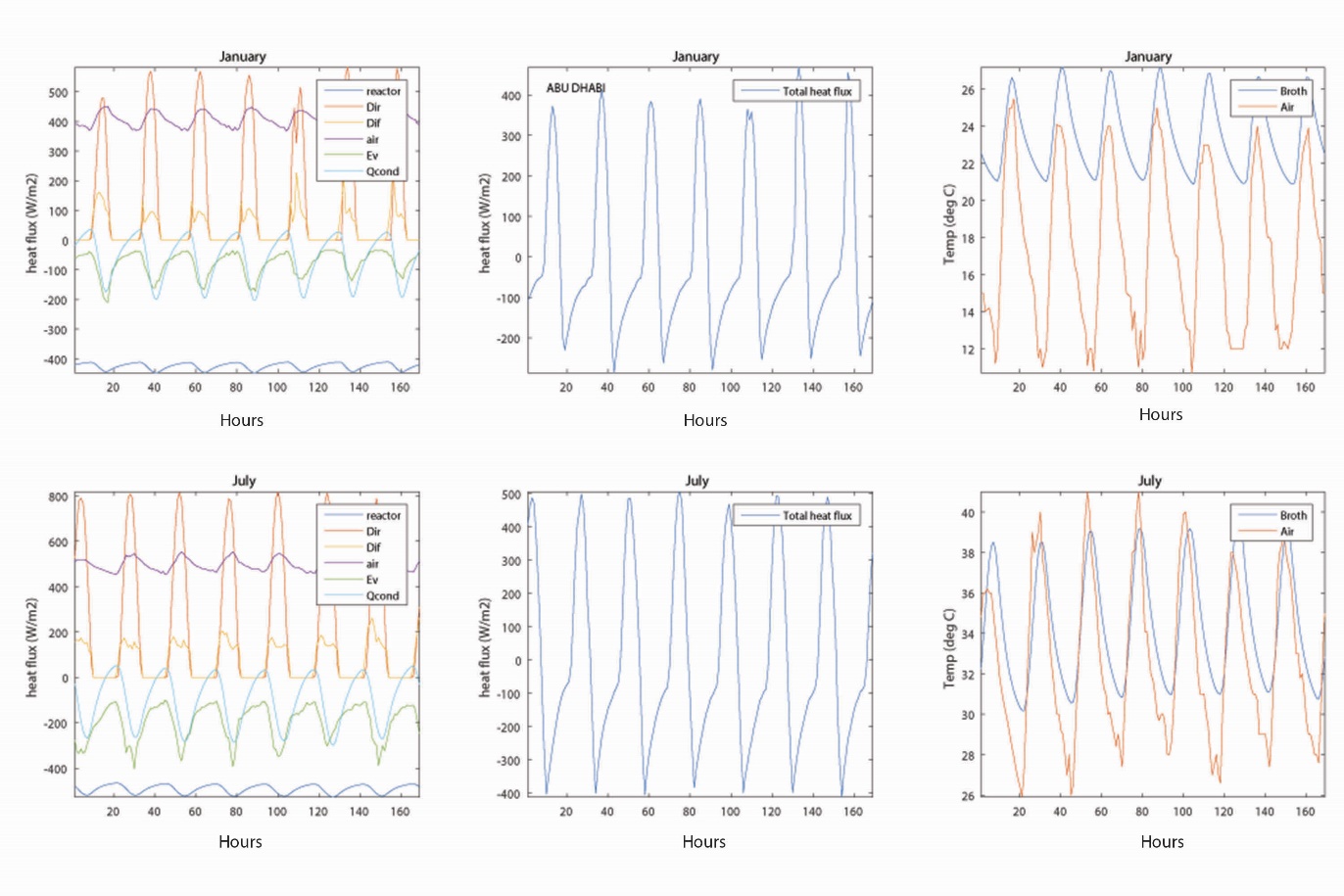


**Figure S4.** Temperature variations in **Abu Dhabi, United Arab Emirates**. **A)** Hourly temperature of air (actual) and culture media (modelled). Daily mean **(B)** and min and max **(C)** air (actual) and culture temperature (modelled). Daily mean **(D)** evaporation, **(E)** global horizontal solar radiation, and **(F)** relative humidity. Comparison of a typical week in January and July showing relative contributions of radiation sources **(G, J)** to total changes in heat flux **(H, K)** and; the consequence to differences of model predicted culture temperature relative air temperature **(I, L)**.


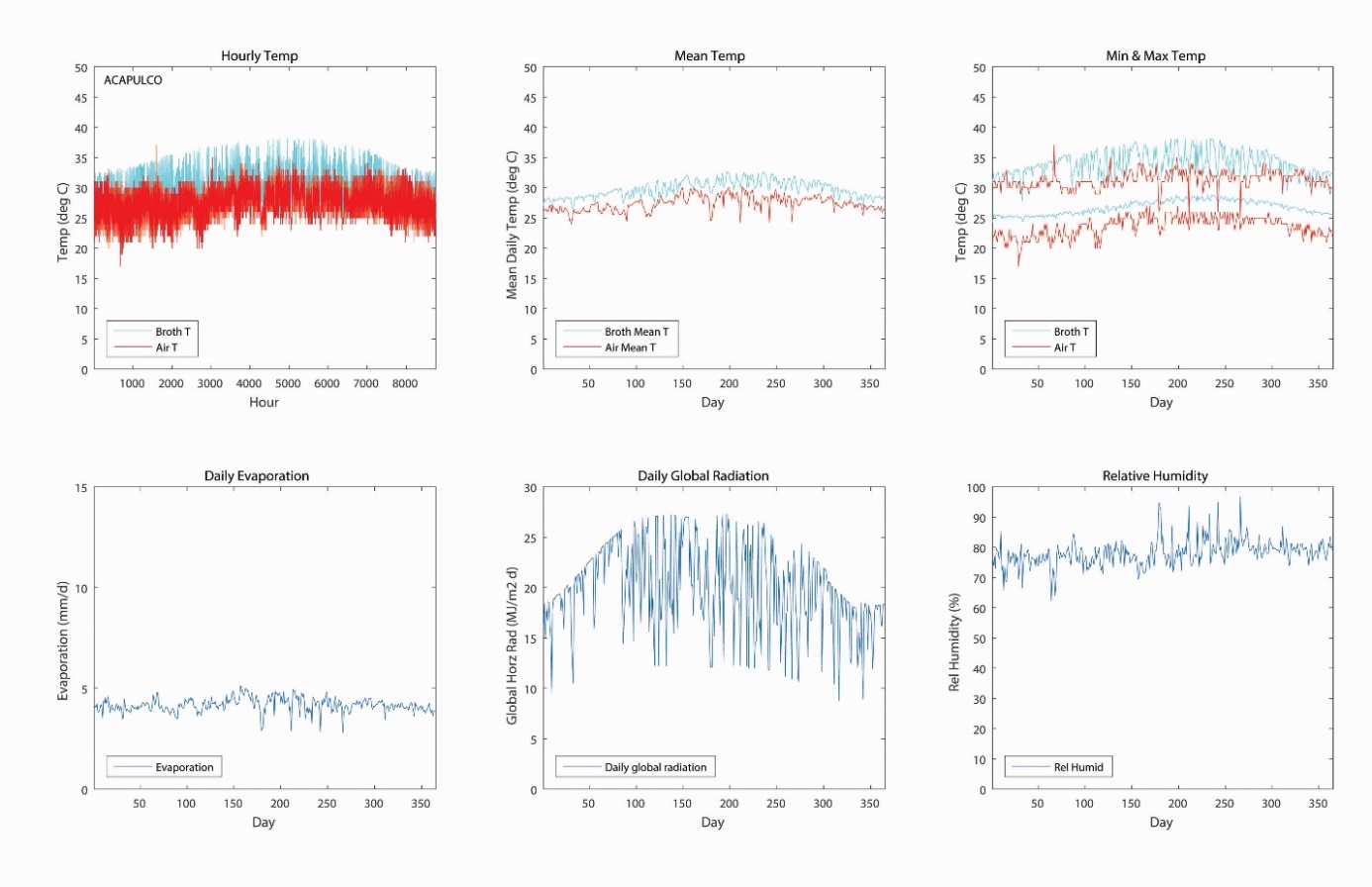

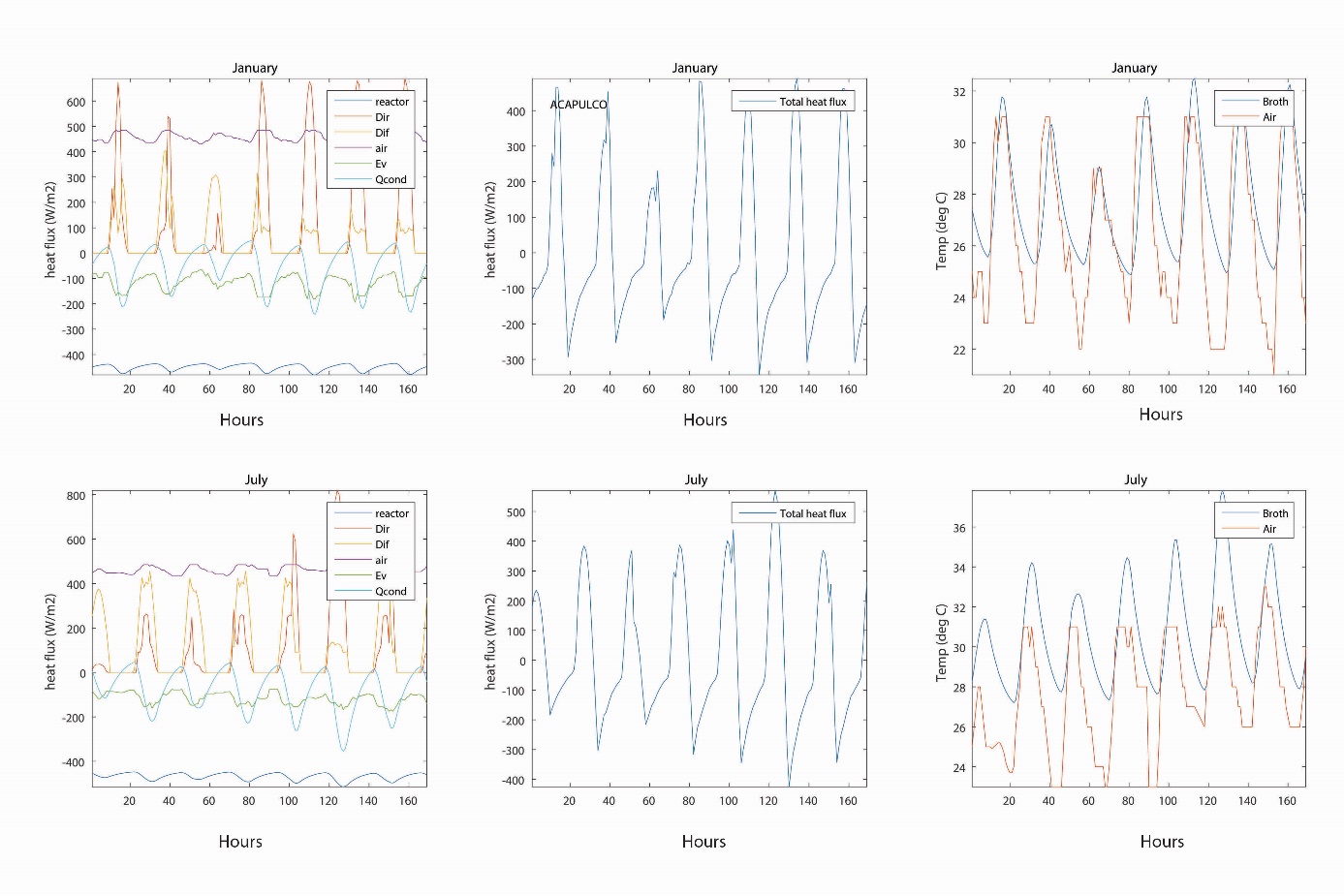


**Figure S5.** Temperature variations in **Acapulco, Mexico**. **A)** Hourly temperature of air (actual) and culture media (modelled). Daily mean **(B)** and min and max **(C)** air (actual) and culture temperature (modelled). Daily mean **(D)** evaporation, **(E)** global horizontal solar radiation, and **(F)** relative humidity. Comparison of a typical week in January and July showing relative contributions of radiation sources **(G, J)** to total changes in heat flux **(H, K)** and; the consequence to differences of model predicted culture temperature relative air temperature **(I, L)**.


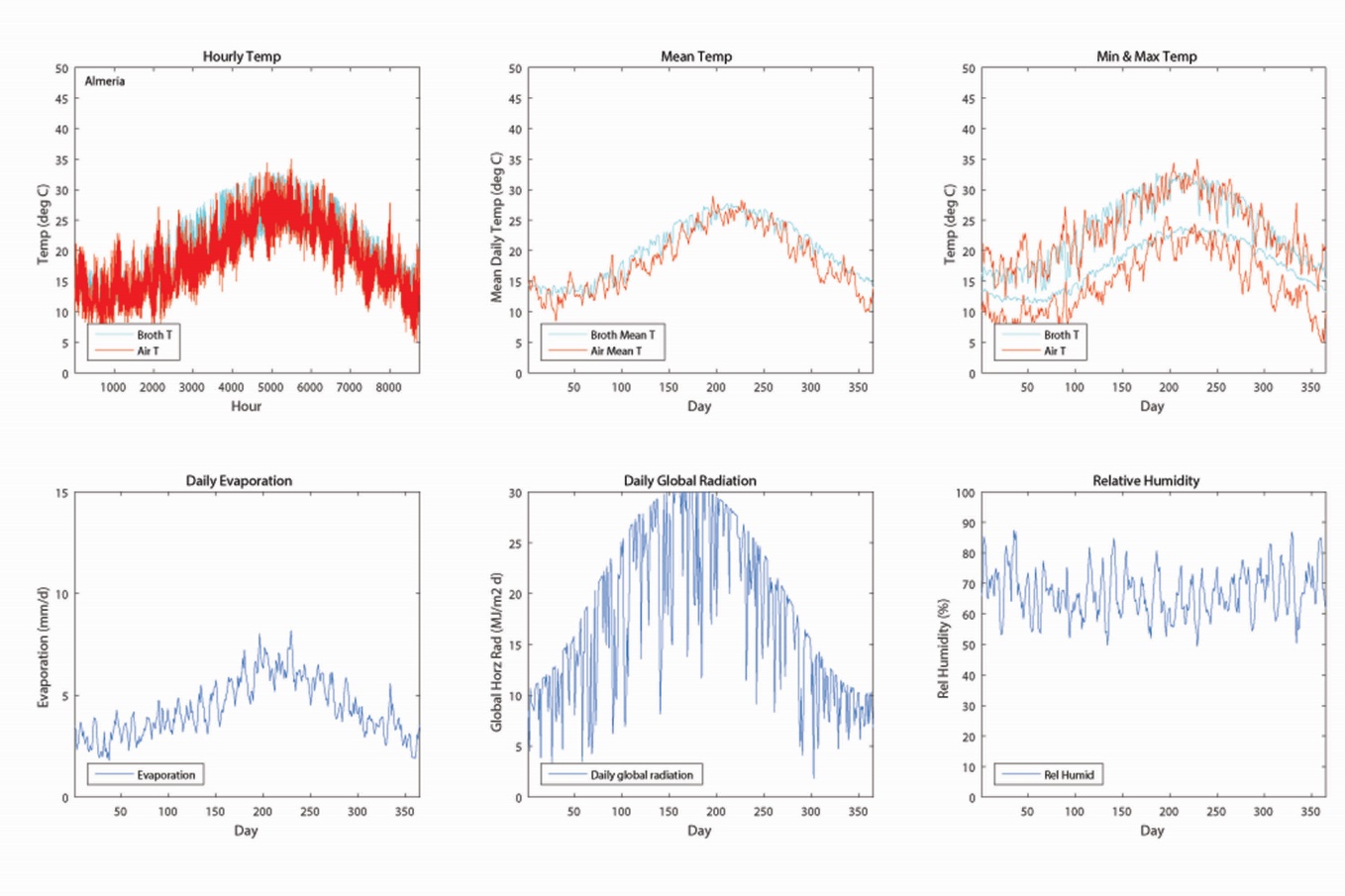

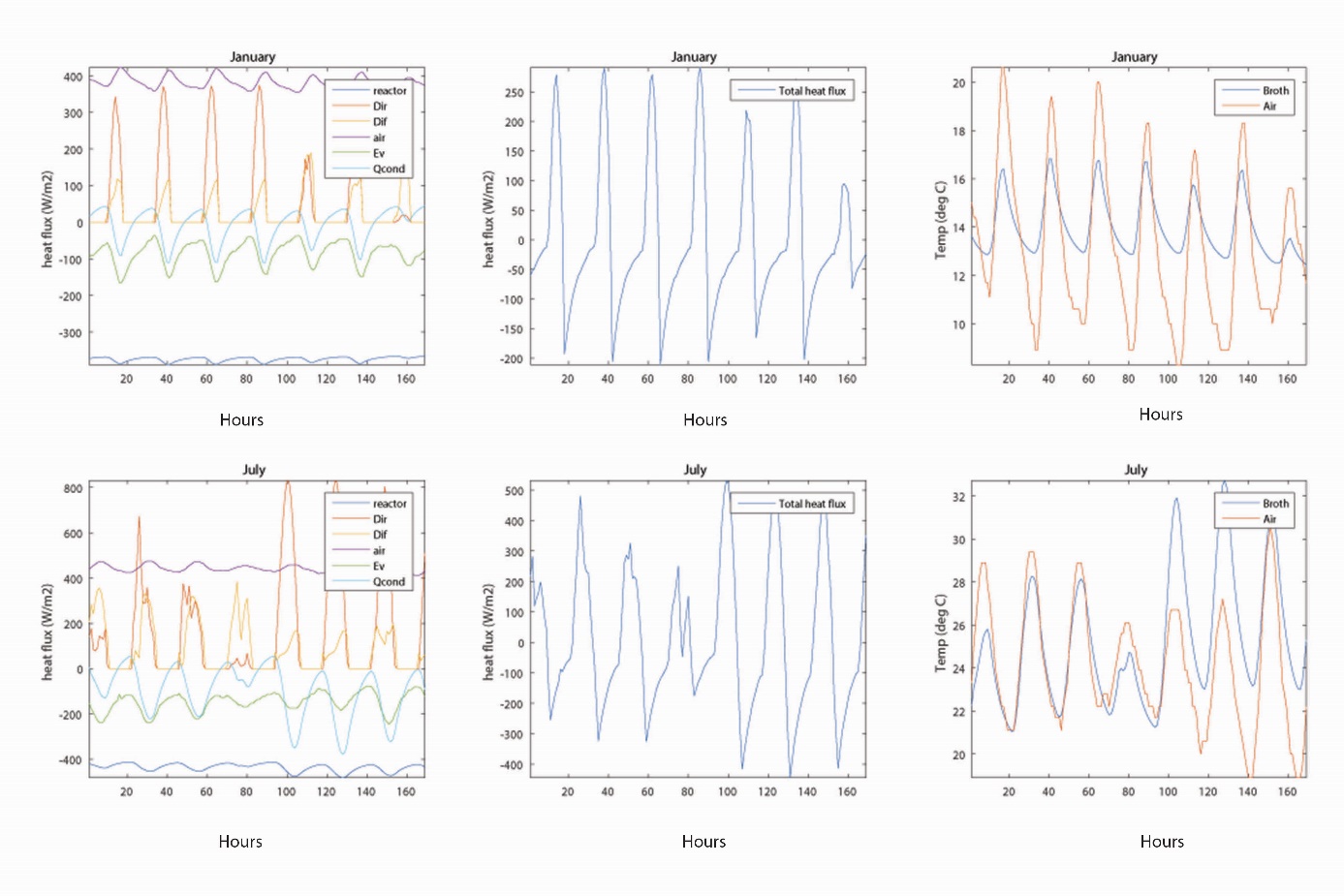


**Figure S6.** Temperature variations in **Almeria, Spain**. **A)** Hourly temperature of air (actual) and culture media (modelled). Daily mean **(B)** and min and max **(C)** air (actual) and culture temperature (modelled). Daily mean **(D)** evaporation, **(E)** global horizontal solar radiation, and **(F)** relative humidity. Comparison of a typical week in January and July showing relative contributions of radiation sources **(G, J)** to total changes in heat flux **(H, K)** and; the consequence to differences of model predicted culture temperature relative air temperature **(I, L)**.


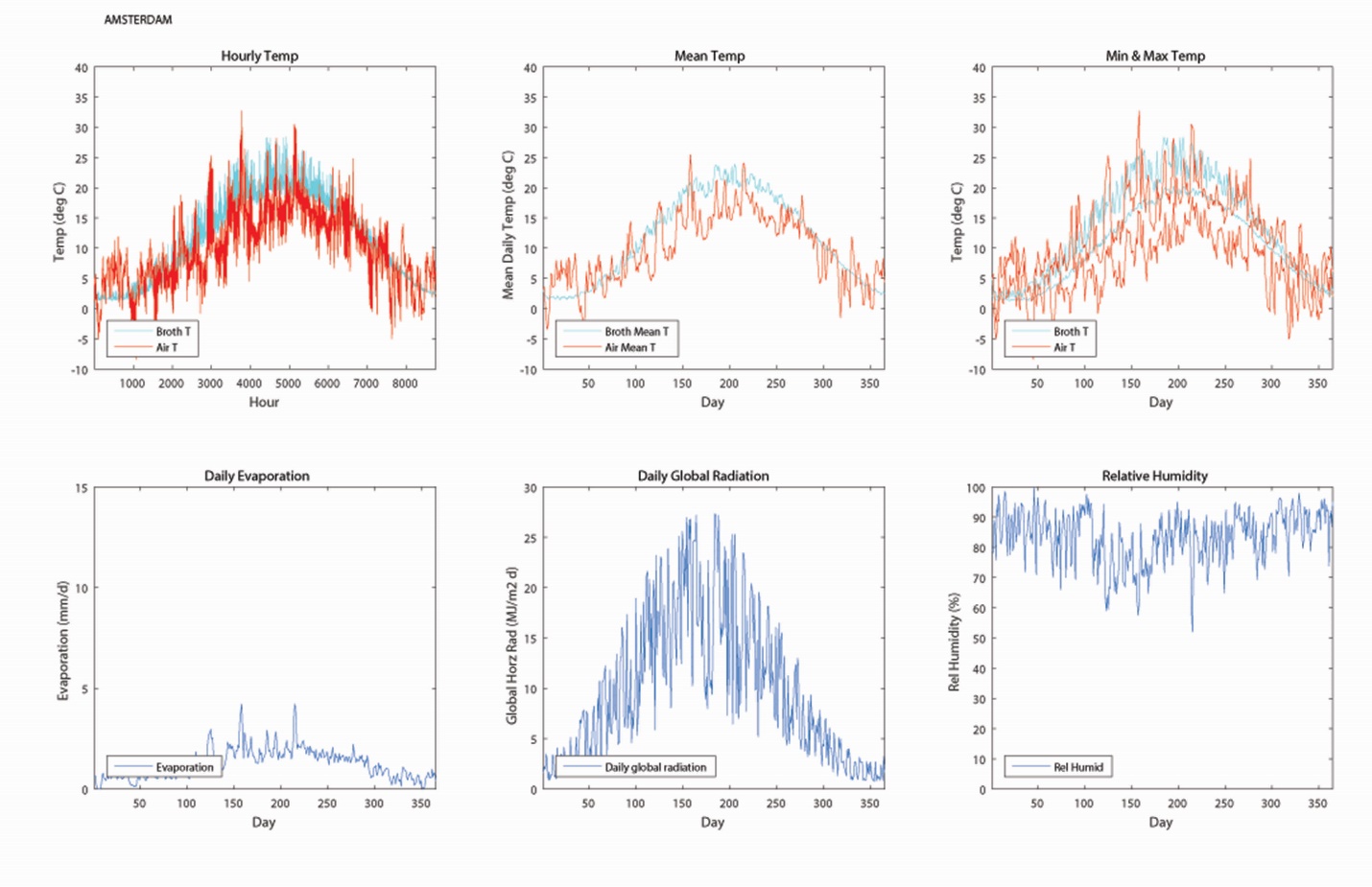


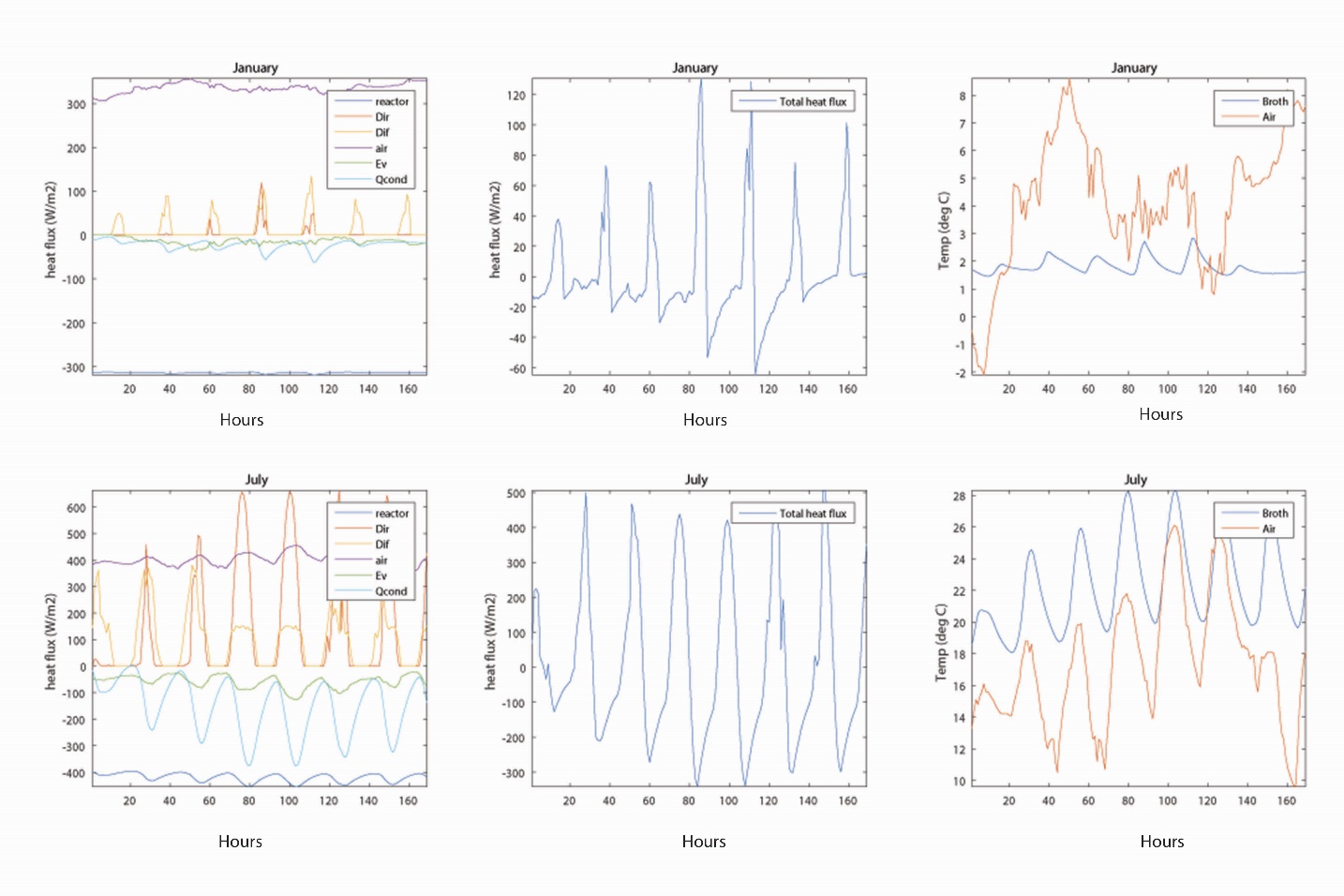


**Figure S7.** Temperature variations in **Amsterdam, The Netherlands**. **A)** Hourly temperature of air (actual) and culture media (modelled). Daily mean **(B)** and min and max **(C)** air (actual) and culture temperature (modelled). Daily mean **(D)** evaporation, **(E)** global horizontal solar radiation, and **(F)** relative humidity. Comparison of a typical week in January and July showing relative contributions of radiation sources **(G, J)** to total changes in heat flux **(H, K)** and; the consequence to differences of model predicted culture temperature relative air temperature **(I, L)**.


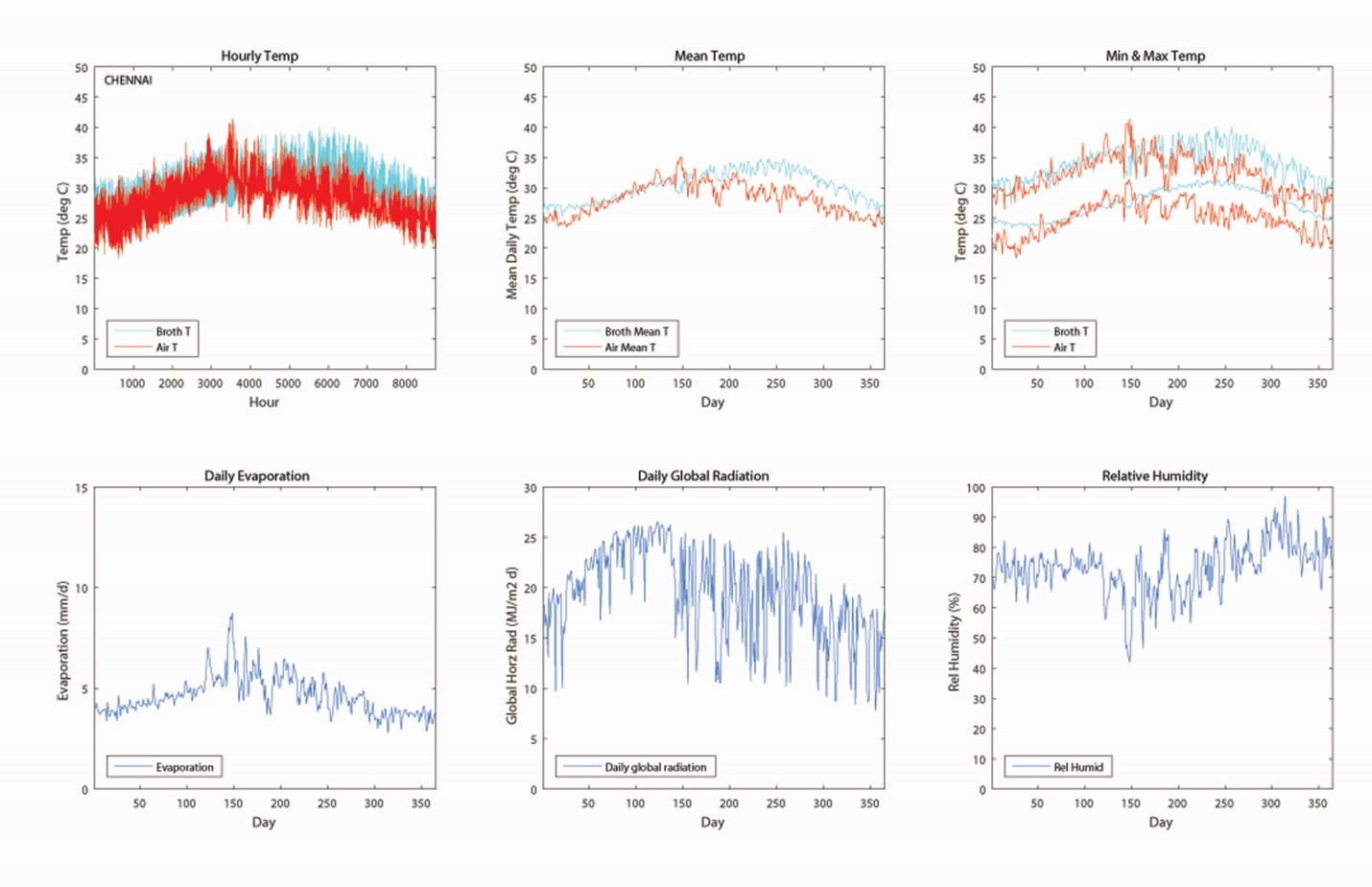

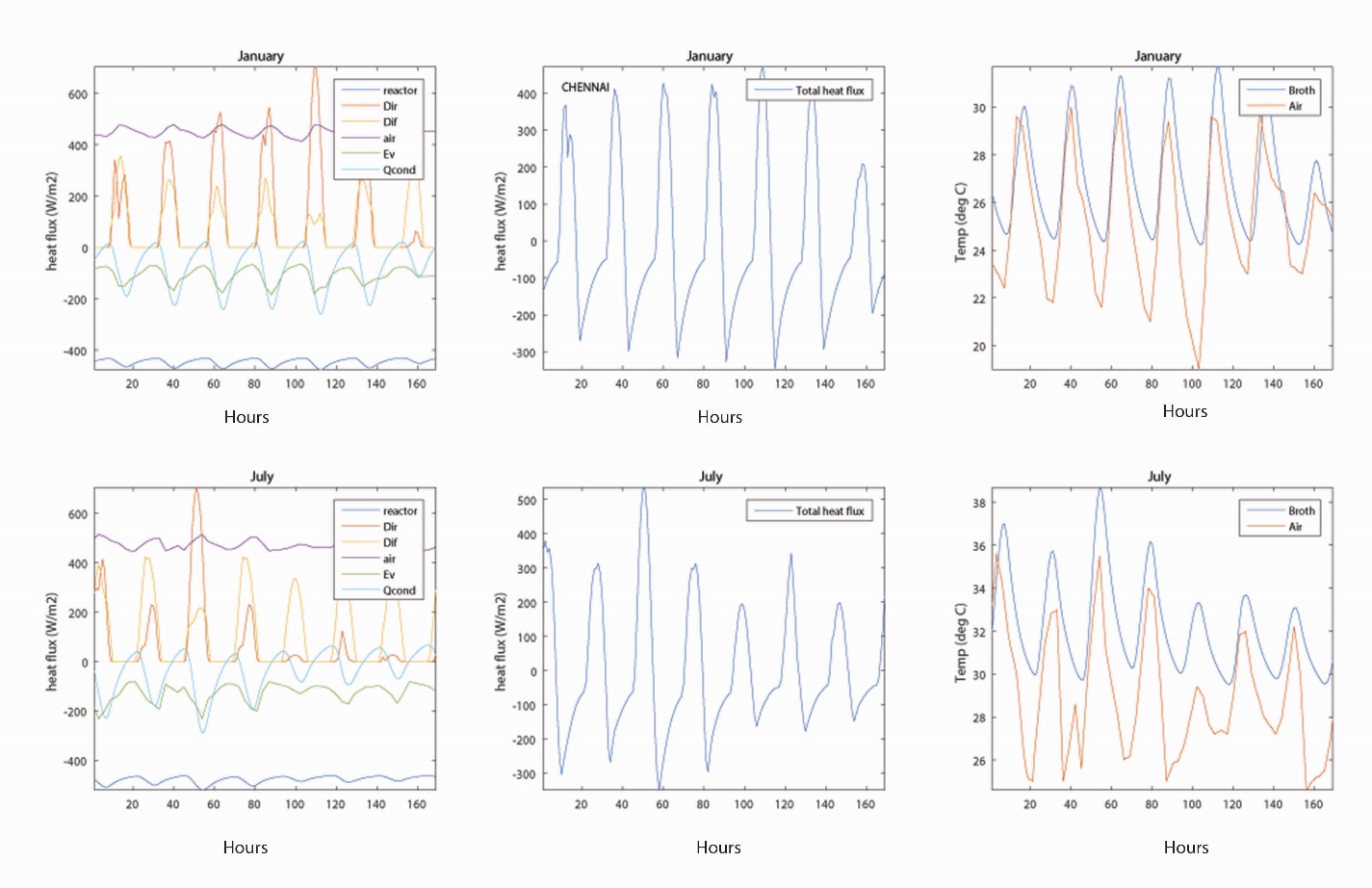


**Figure S8.** Temperature variations in **Chennai, India.** **A)** Hourly temperature of air (actual) and culture media (modelled). Daily mean **(B)** and min and max **(C)** air (actual) and culture temperature (modelled). Daily mean **(D)** evaporation, **(E)** global horizontal solar radiation, and **(F)** relative humidity. Comparison of a typical week in January and July showing relative contributions of radiation sources **(G, J)** to total changes in heat flux **(H, K)** and; the consequence to differences of model predicted culture temperature relative air temperature **(I, L)**.


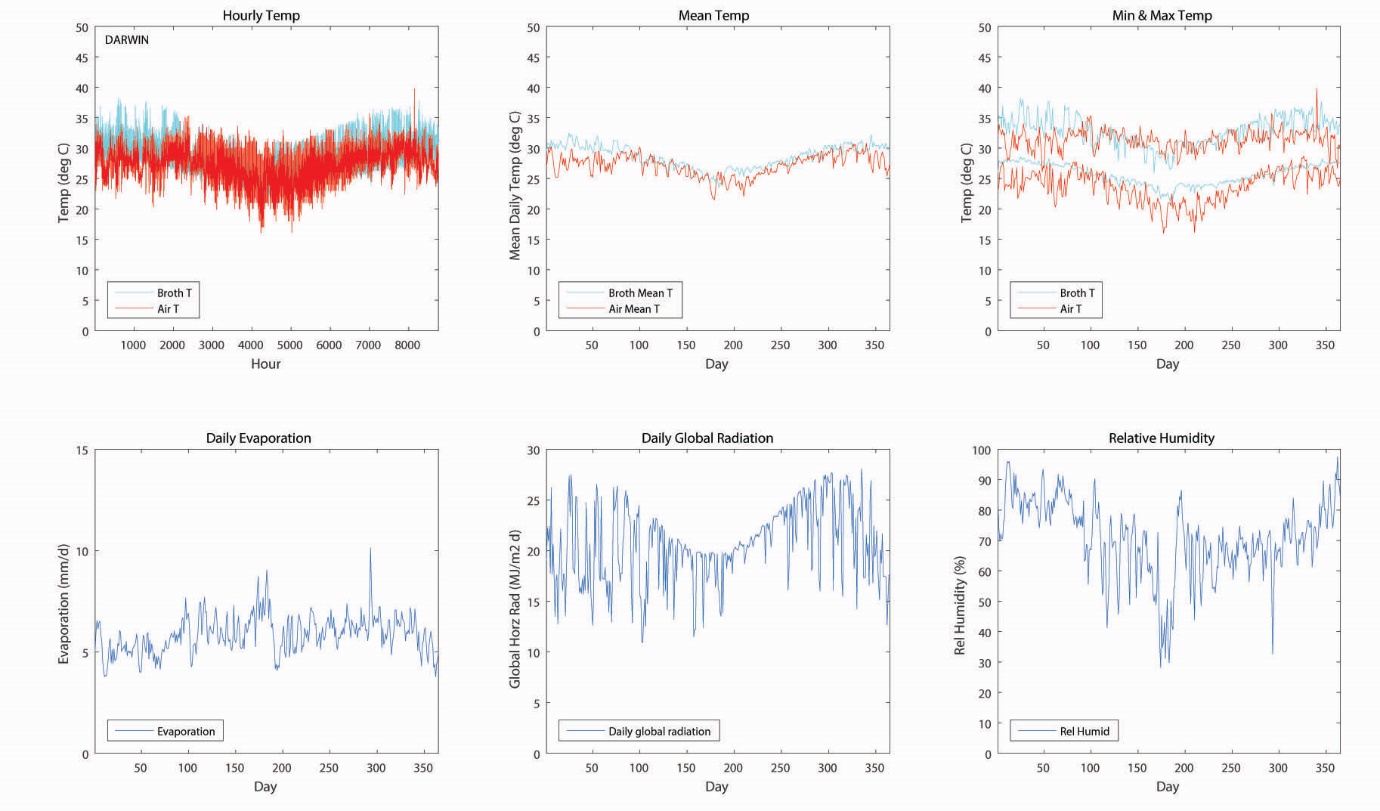

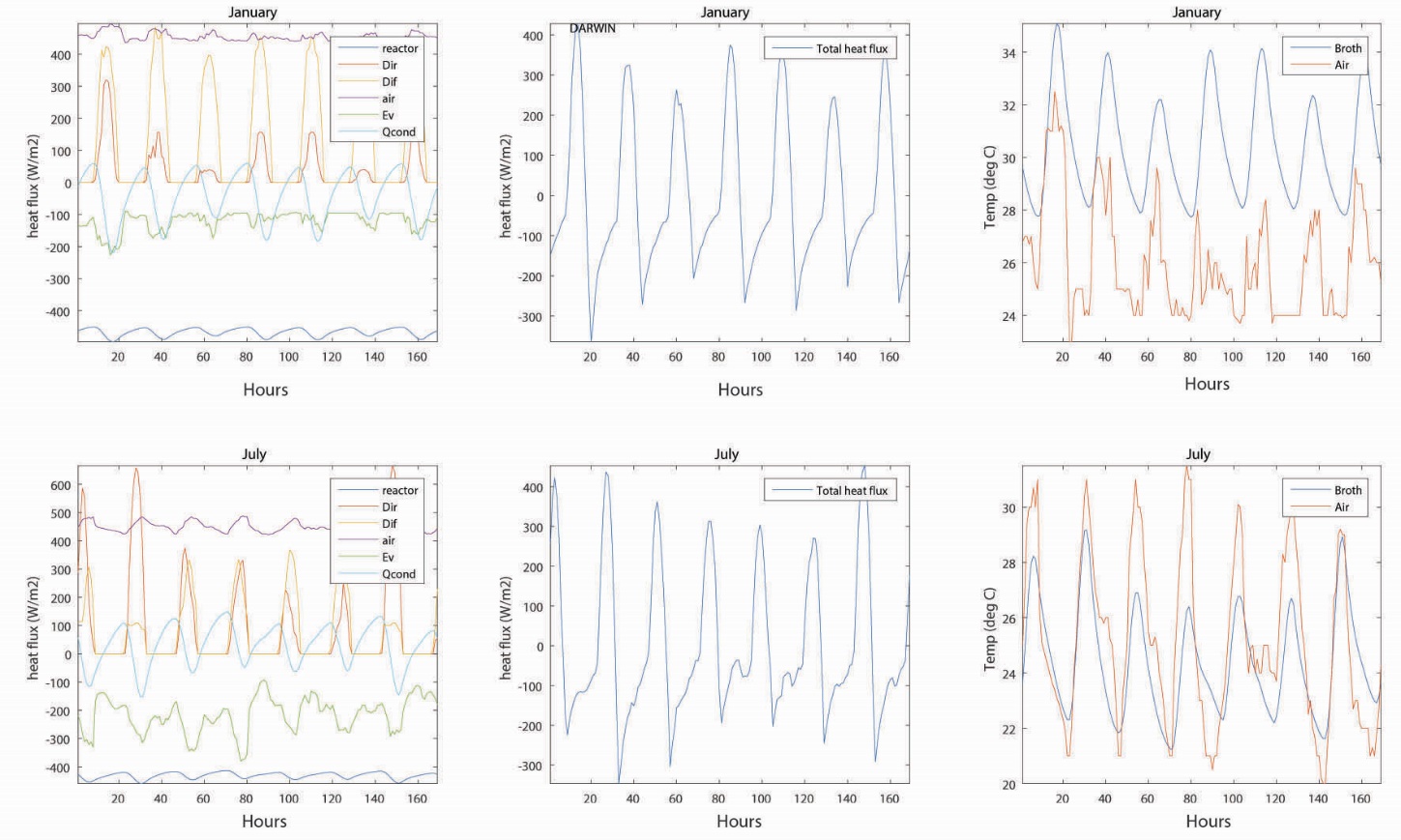


**Figure S9.** Temperature variations in **Darwin, Australia**. **A)** Hourly temperature of air (actual) and culture media (modelled). Daily mean **(B)** and min and max **(C)** air (actual) and culture temperature (modelled). Daily mean **(D)** evaporation, **(E)** global horizontal solar radiation, and **(F)** relative humidity. Comparison of a typical week in January and July showing relative contributions of radiation sources **(G, J)** to total changes in heat flux **(H, K)** and; the consequence to differences of model predicted culture temperature relative air temperature **(I, L)**.


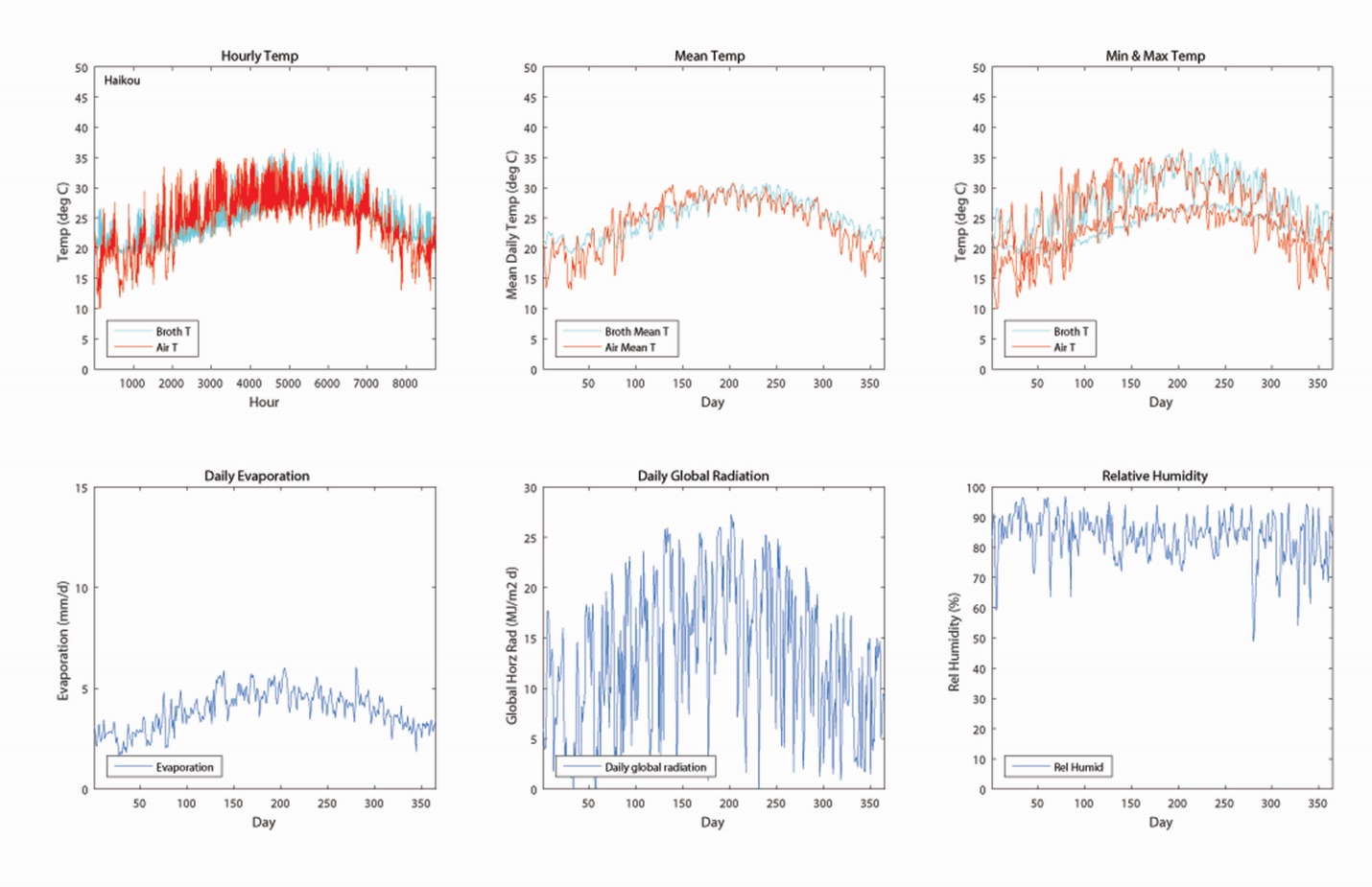

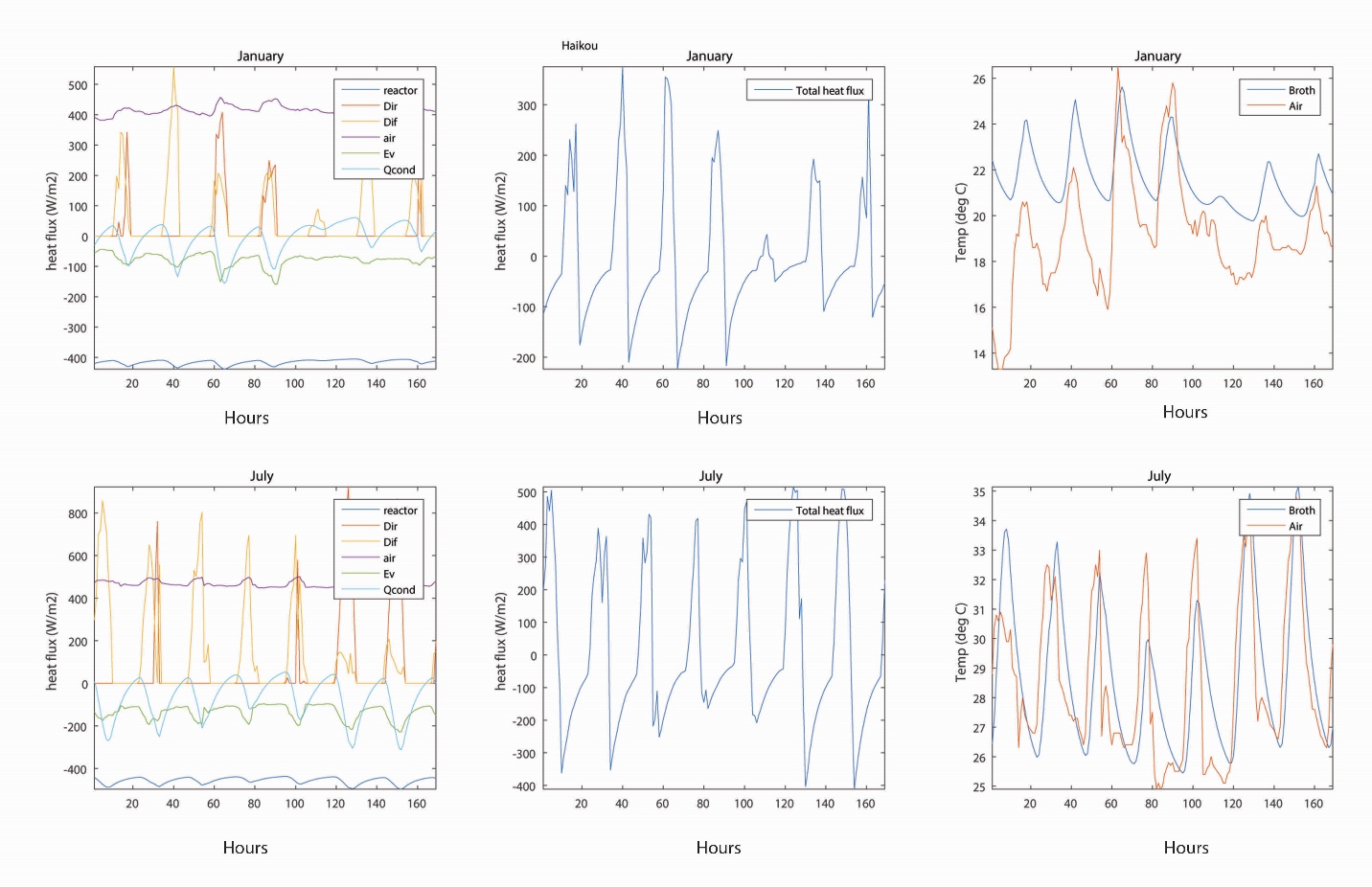


**Figure S10.** Temperature variations in **Haikou, China**. **A)** Hourly temperature of air (actual) and culture media (modelled). Daily mean **(B)** and min and max **(C)** air (actual) and culture temperature (modelled). Daily mean **(D)** evaporation, **(E)** global horizontal solar radiation, and **(F)** relative humidity. Comparison of a typical week in January and July showing relative contributions of radiation sources **(G, J)** to total changes in heat flux **(H, K)** and; the consequence to differences of model predicted culture temperature relative air temperature **(I, L)**.


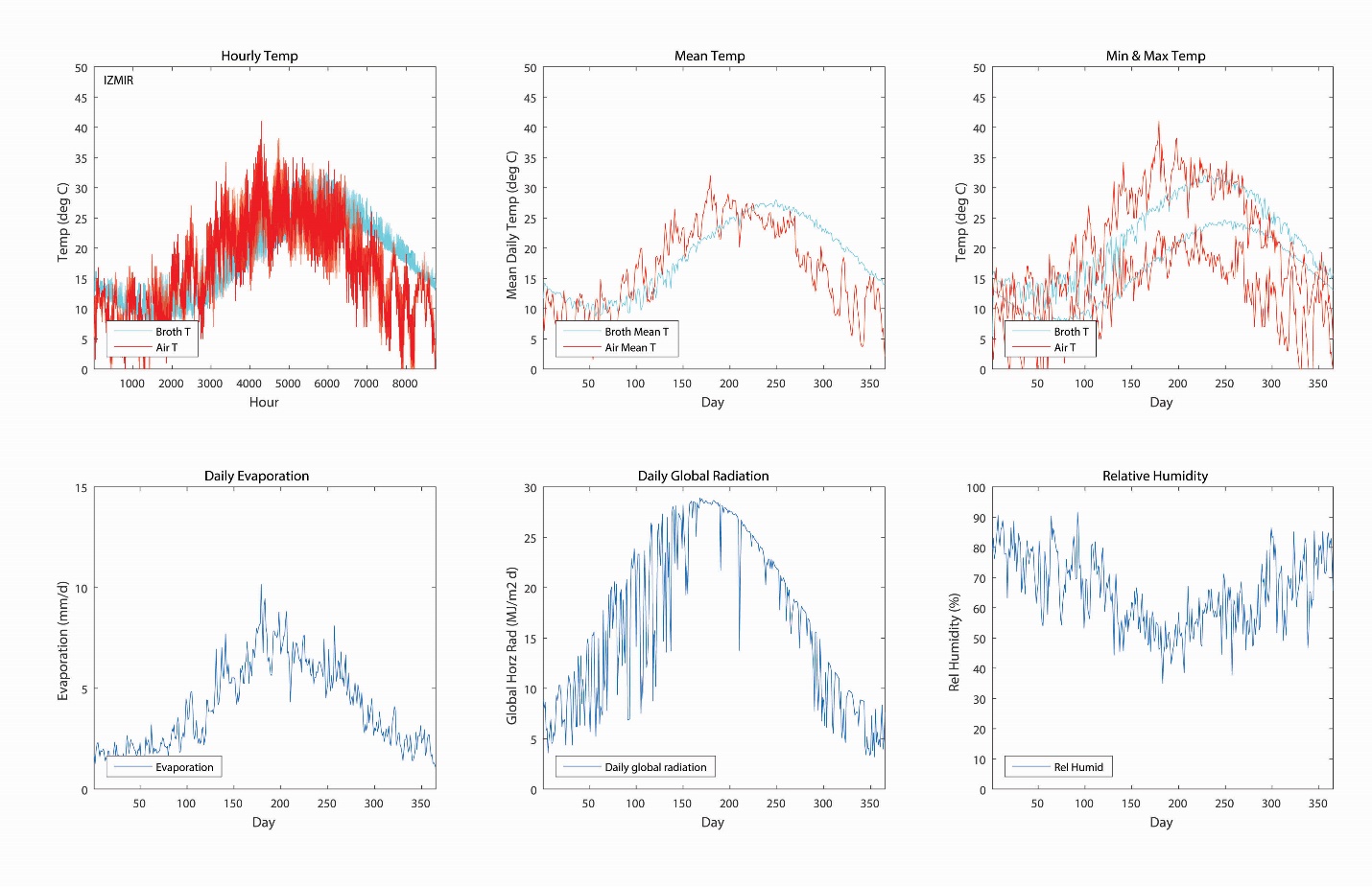

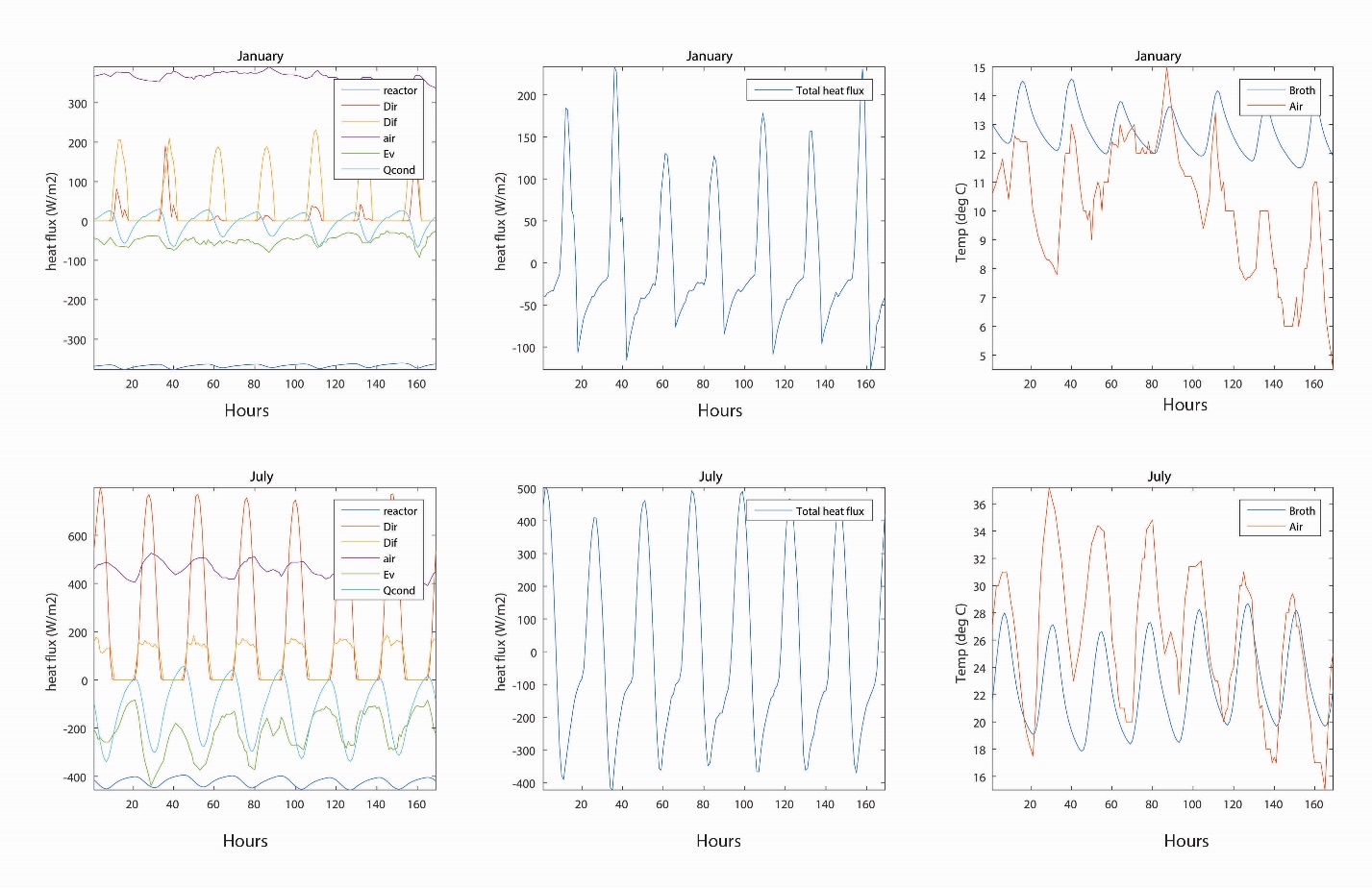


**Figure S11.** Temperature variations in **Izmir, Turkey**. **A)** Hourly temperature of air (actual) and culture media (modelled). Daily mean **(B)** and min and max **(C)** air (actual) and culture temperature (modelled). Daily mean **(D)** evaporation, **(E)** global horizontal solar radiation, and **(F)** relative humidity. Comparison of a typical week in January and July showing relative contributions of radiation sources **(G, J)** to total changes in heat flux **(H, K)** and; the consequence to differences of model predicted culture temperature relative air temperature **(I, L)**.


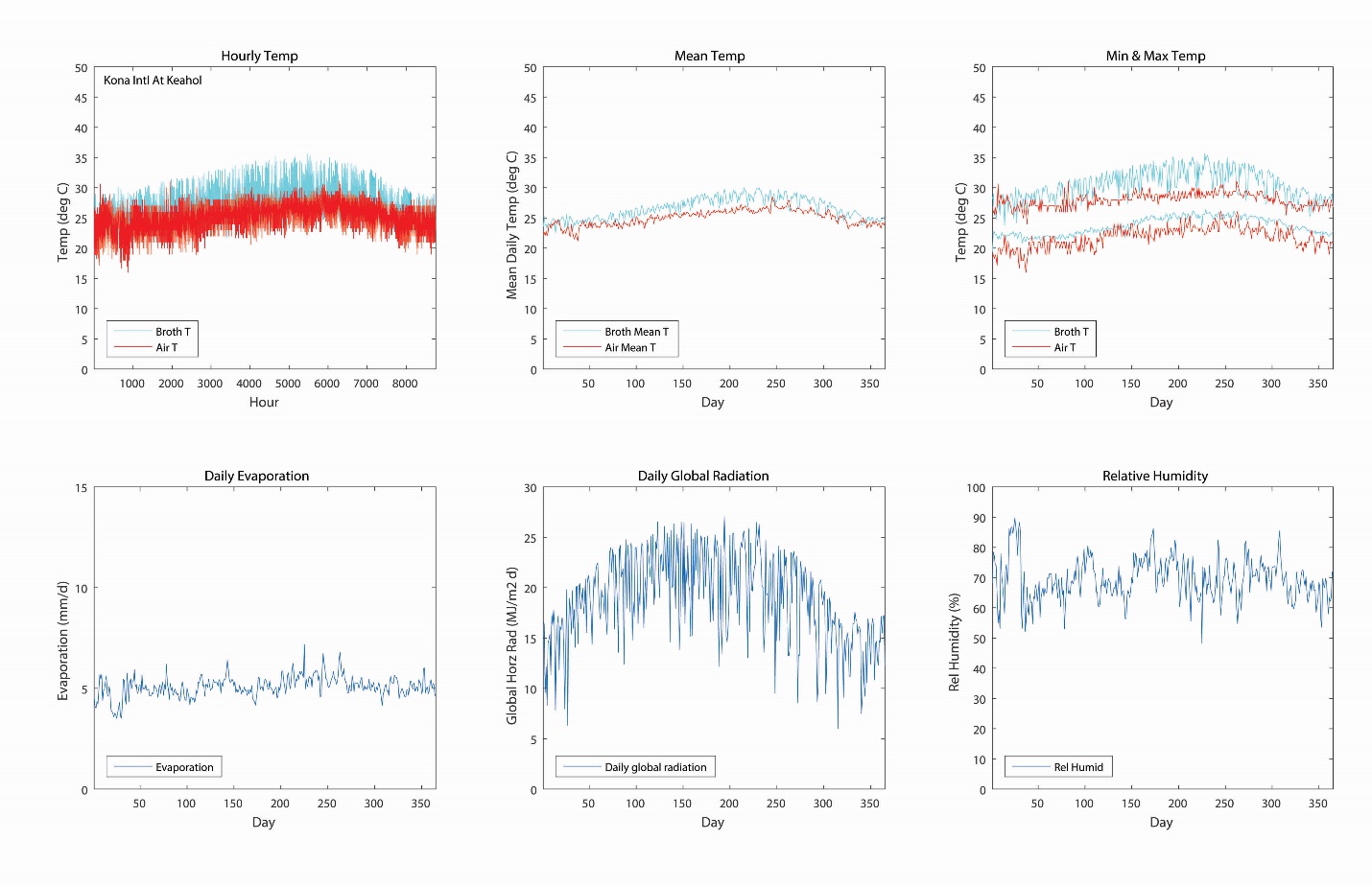

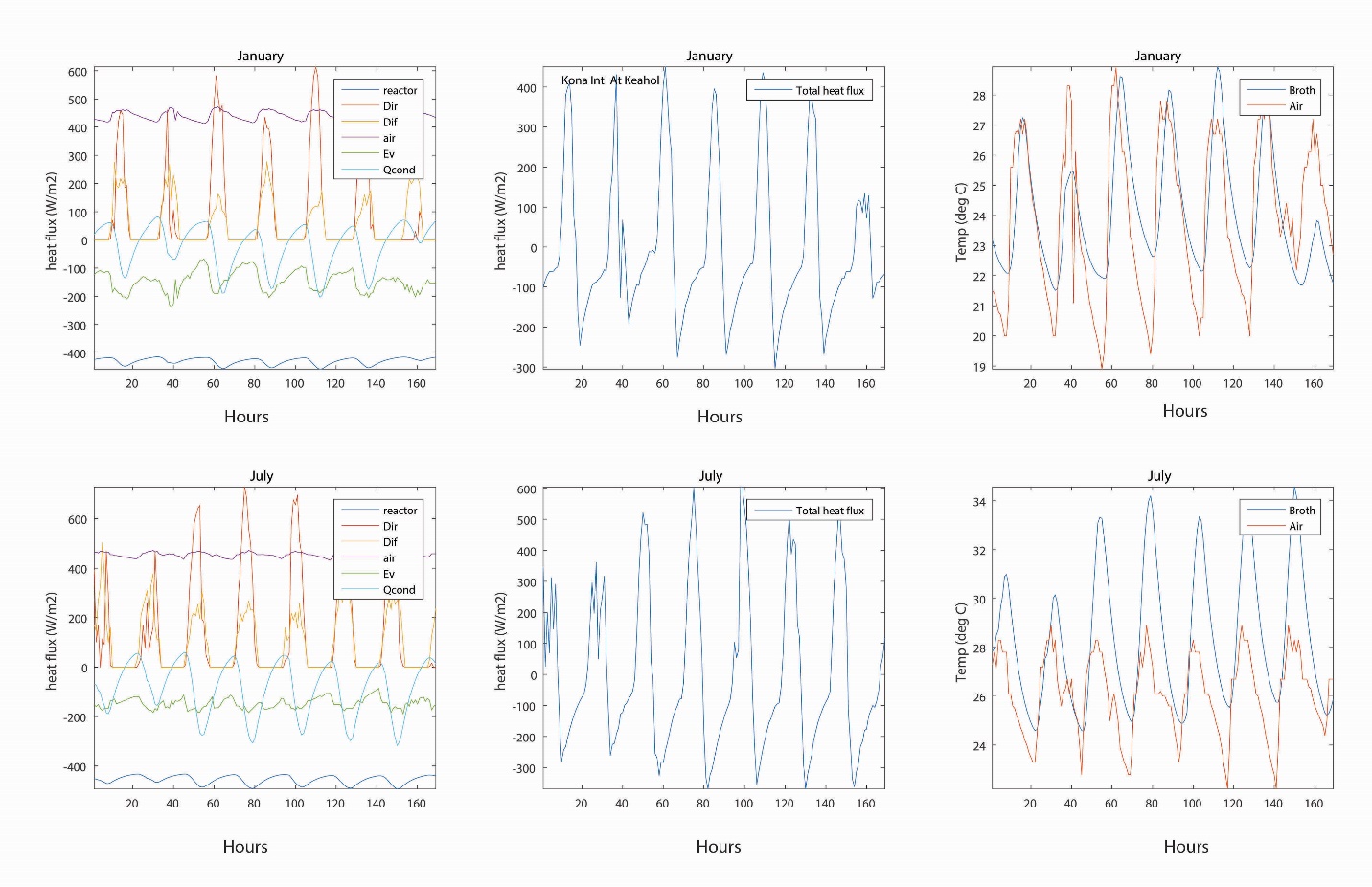


**Figure S12.** Temperature variations in **Kona, USA**. **A)** Hourly temperature of air (actual) and culture media (modelled). Daily mean **(B)** and min and max **(C)** air (actual) and culture temperature (modelled). Daily mean **(D)** evaporation, **(E)** global horizontal solar radiation, and **(F)** relative humidity. Comparison of a typical week in January and July showing relative contributions of radiation sources **(G, J)** to total changes in heat flux **(H, K)** and; the consequence to differences of model predicted culture temperature relative air temperature **(I, L)**.


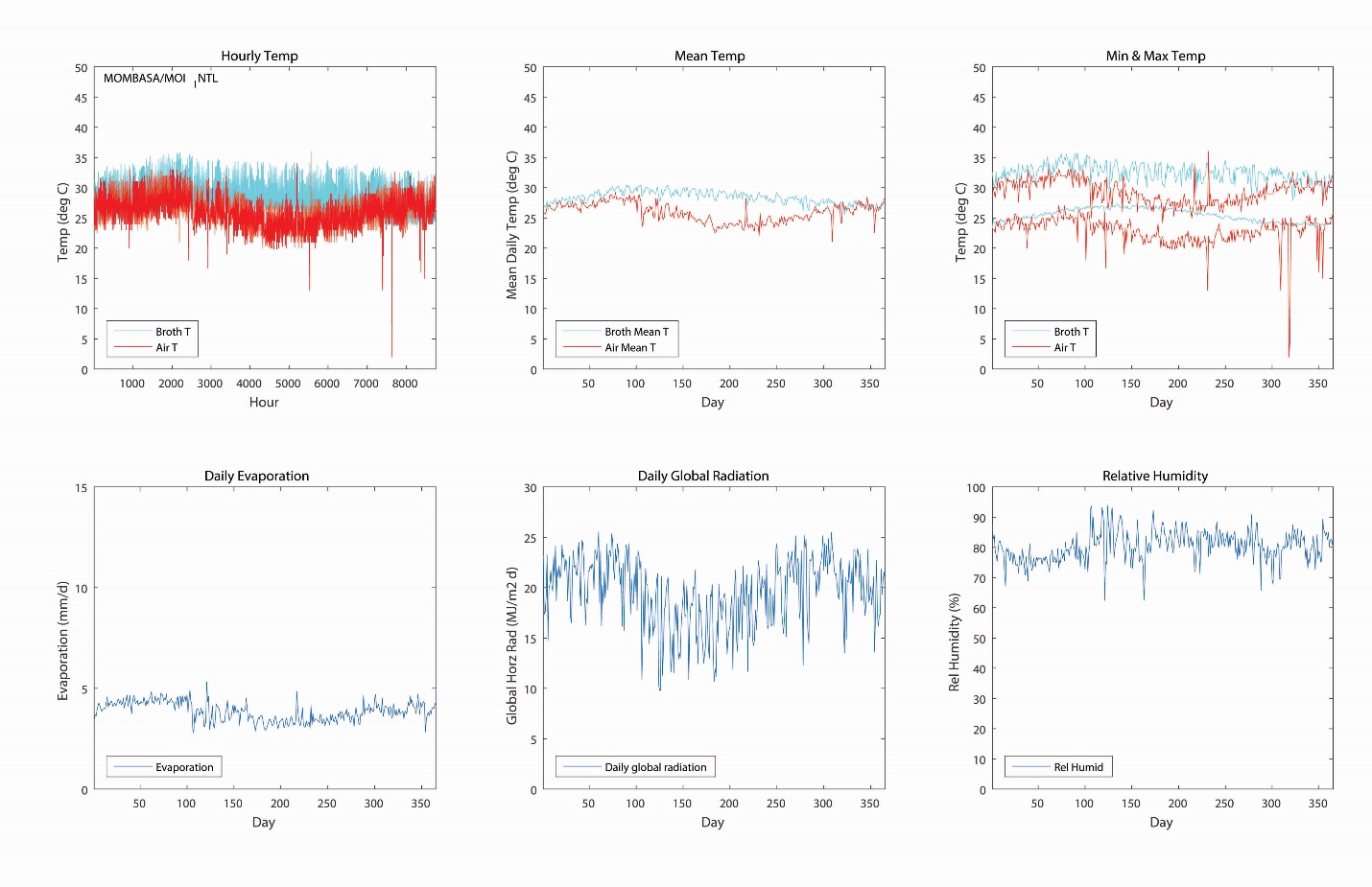

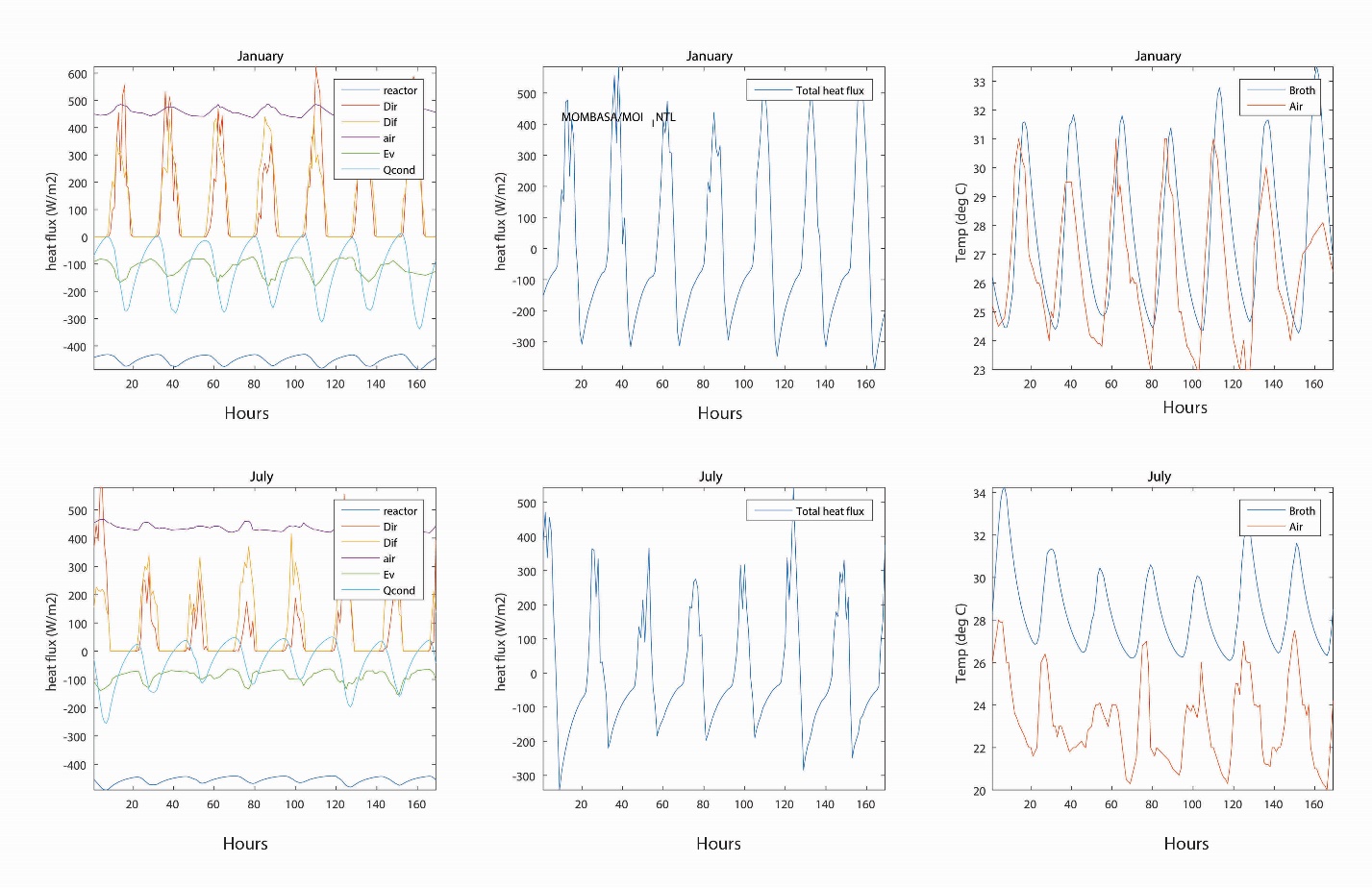


**Figure S13.** Temperature variations in **Mombasa, Kenya**. **A)** Hourly temperature of air (actual) and culture media (modelled). Daily mean **(B)** and min and max **(C)** air (actual) and culture temperature (modelled). Daily mean **(D)** evaporation, **(E)** global horizontal solar radiation, and **(F)** relative humidity. Comparison of a typical week in January and July showing relative contributions of radiation sources **(G, J)** to total changes in heat flux **(H, K)** and; the consequence to differences of model predicted culture temperature relative air temperature **(I, L)**.


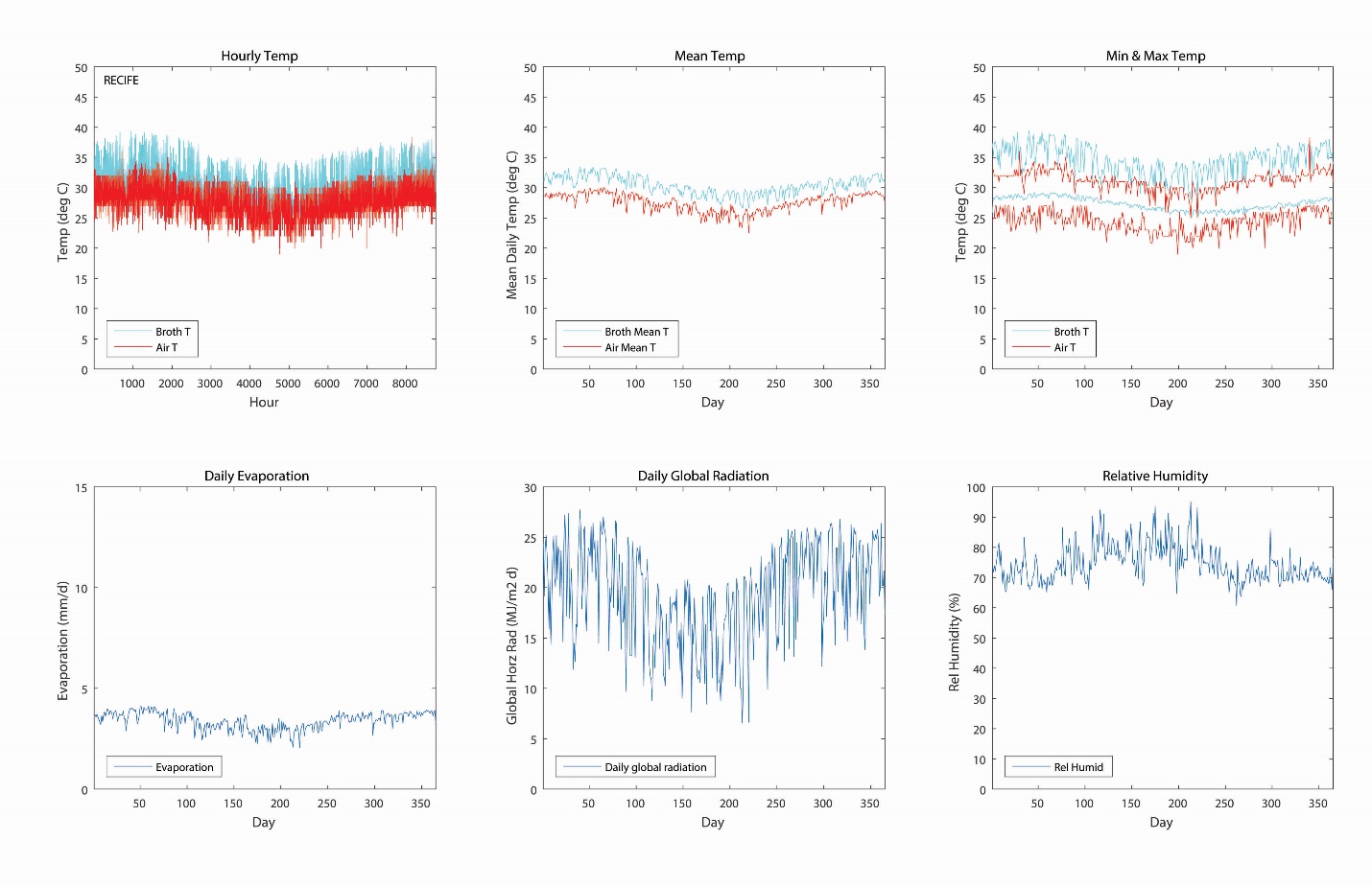

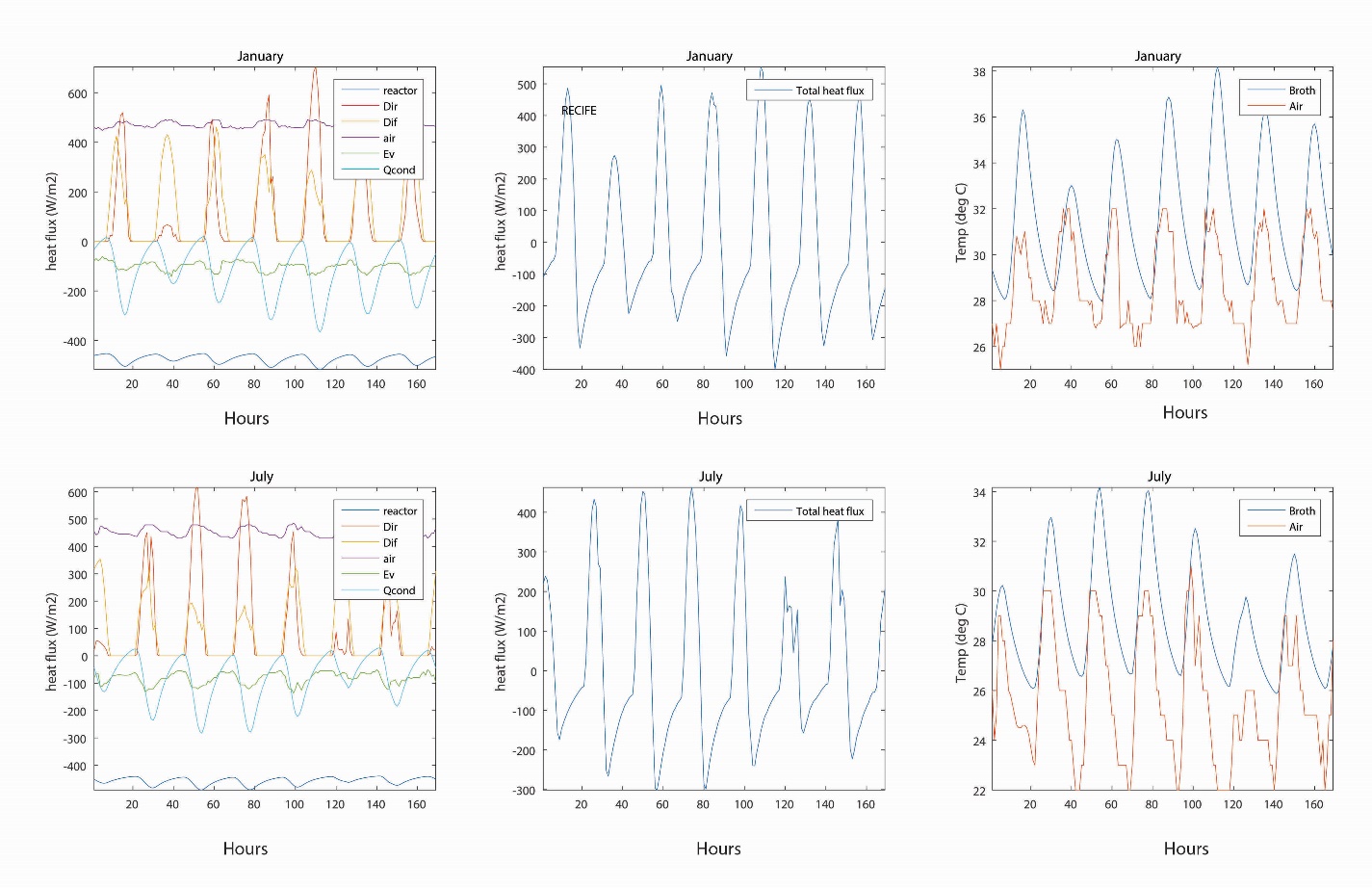


**Figure S14.** Temperature variations in **Recife, Brazil**. **A)** Hourly temperature of air (actual) and culture media (modelled). Daily mean **(B)** and min and max **(C)** air (actual) and culture temperature (modelled). Daily mean **(D)** evaporation, **(E)** global horizontal solar radiation, and **(F)** relative humidity. Comparison of a typical week in January and July showing relative contributions of radiation sources **(G, J)** to total changes in heat flux **(H, K)** and; the consequence to differences of model predicted culture temperature relative air temperature **(I, L)**.


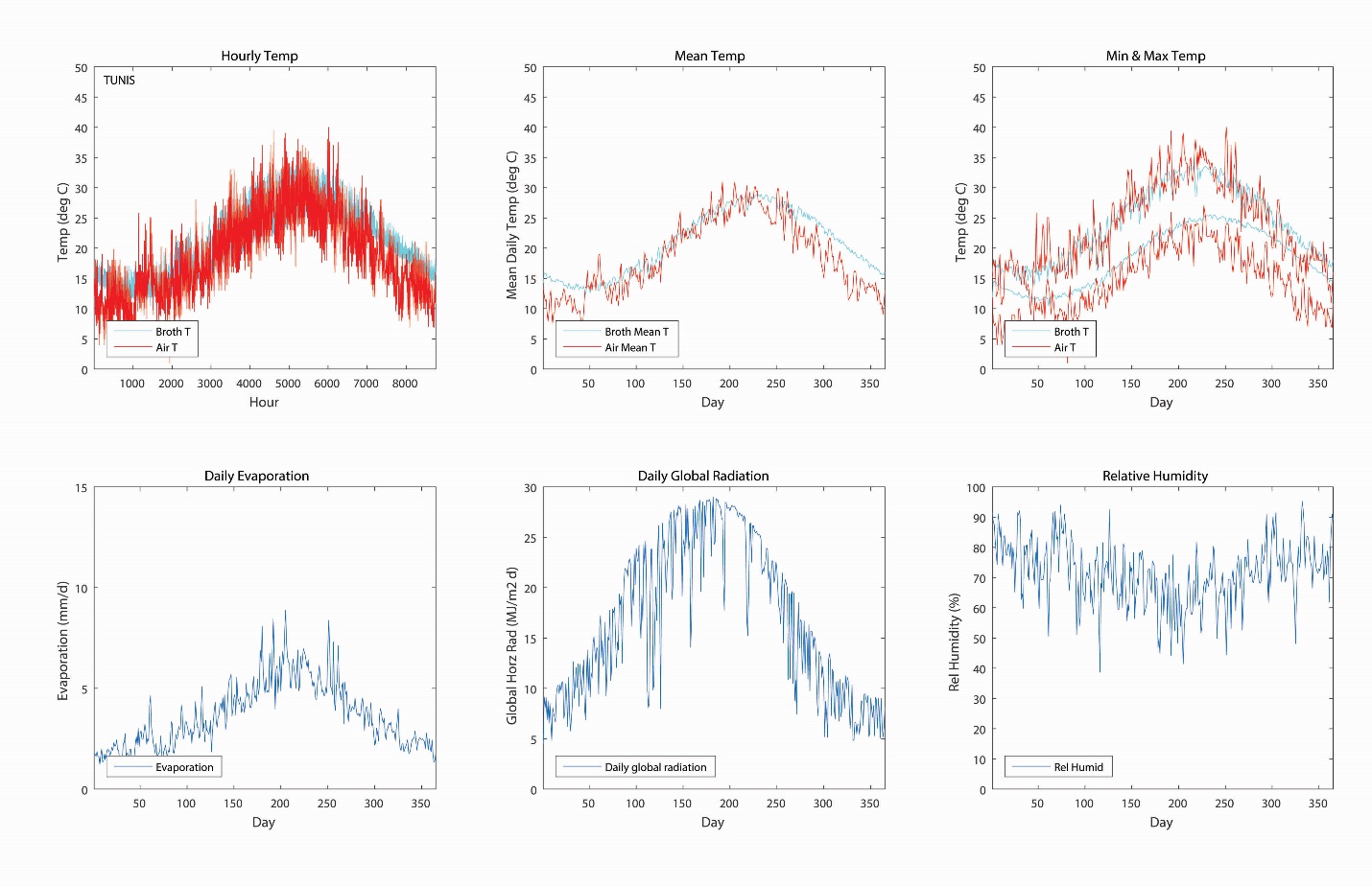

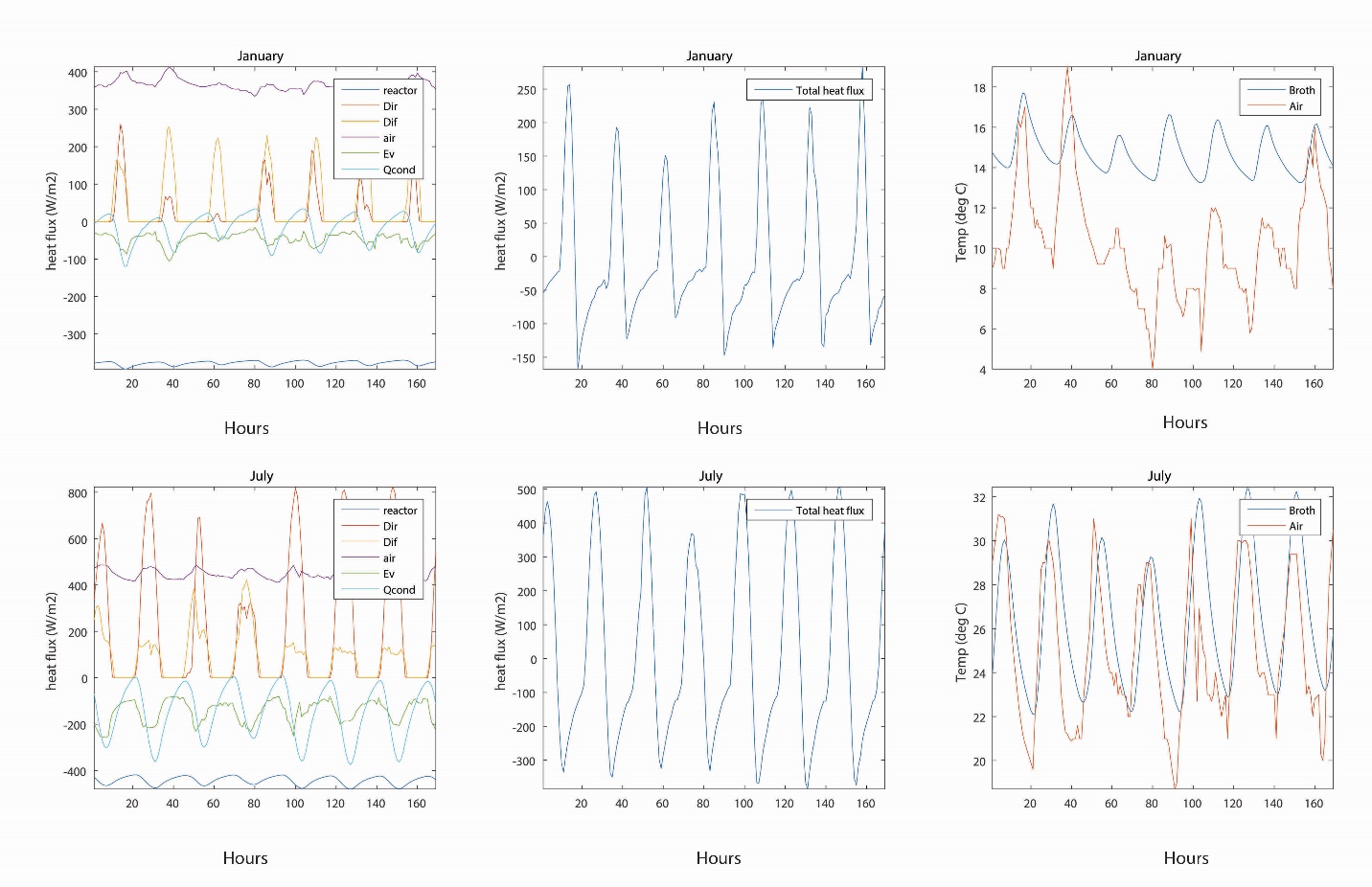


**Figure S15.** Temperature variations in **Tunis, Tunisia**. **A)** Hourly temperature of air (actual) and culture media (modelled). Daily mean **(B)** and min and max **(C)** air (actual) and culture temperature (modelled). Daily mean **(D)** evaporation, **(E)** global horizontal solar radiation, and **(F)** relative humidity. Comparison of a typical week in January and July showing relative contributions of radiation sources **(G, J)** to total changes in heat flux **(H, K)** and; the consequence to differences of model predicted culture temperature relative air temperature **(I, L)**.


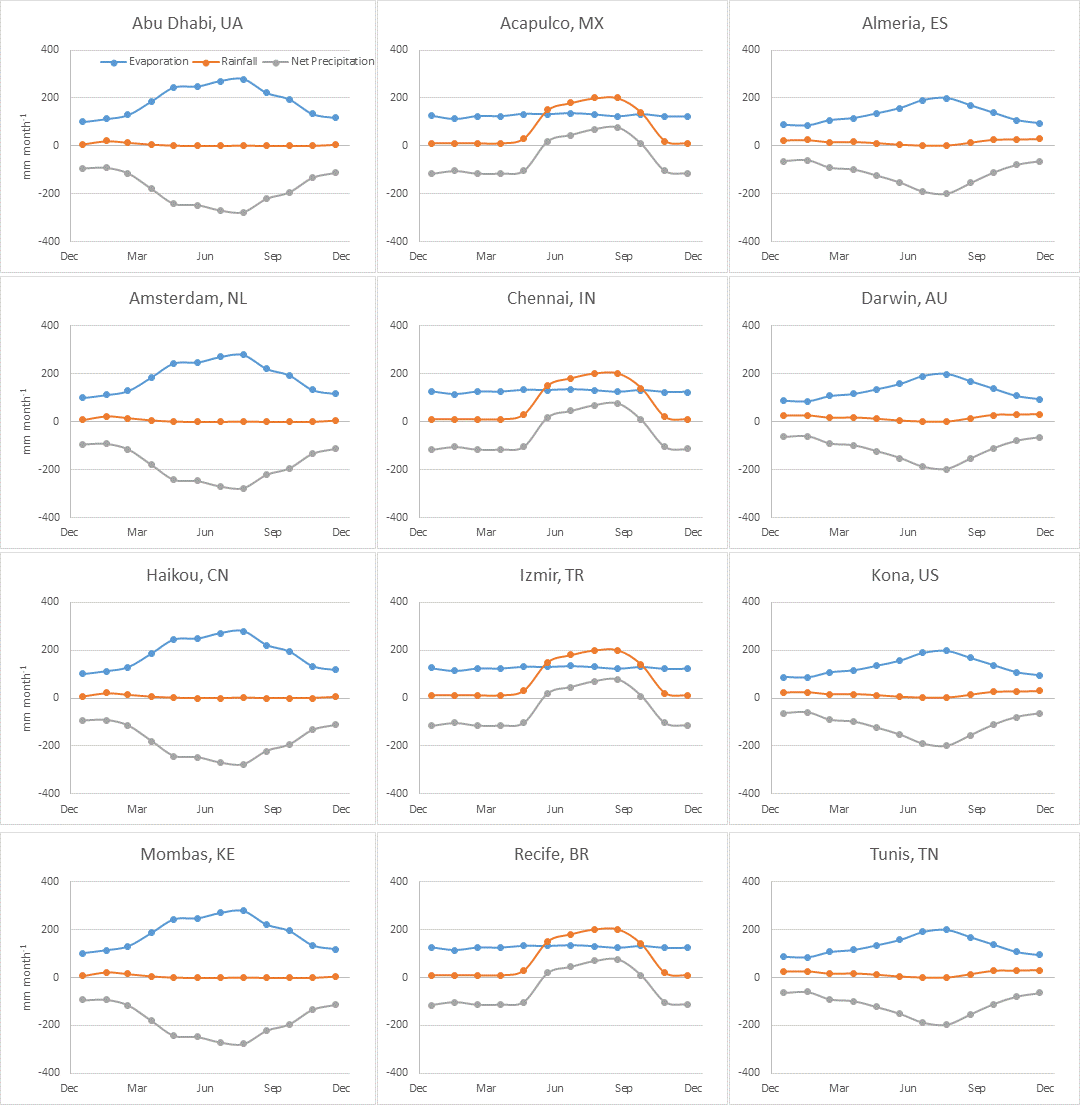


**Figure S16.** Monthly mean evaporation (modelled, blue line), rainfall (actual, orange line) and net precipitation (rainfall – evaporation, grey line) for each location.

**2.6 Simulated productivity data**


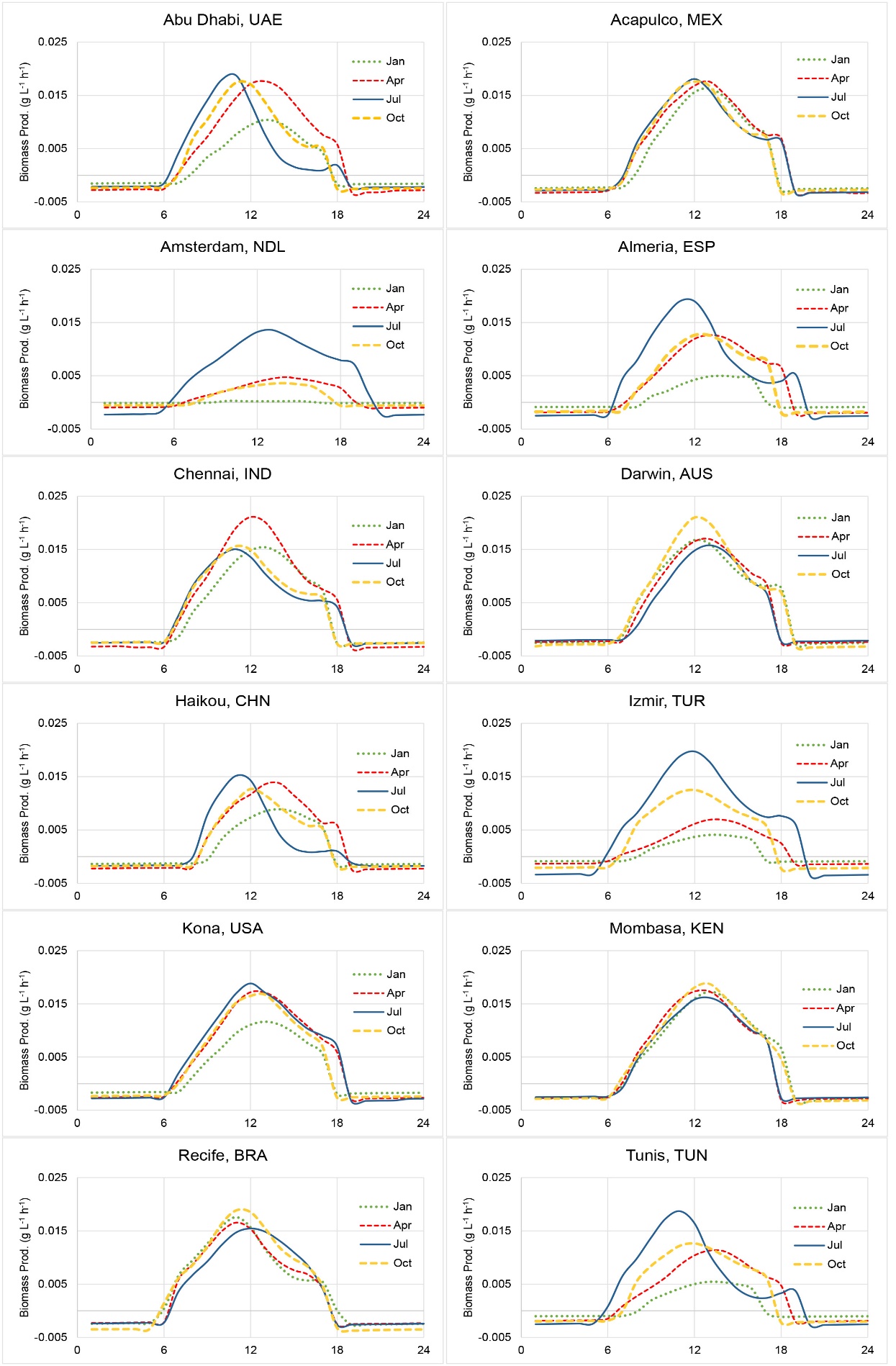


**Figure S17.** Simulation of biomass productivity over the day averaged for four months of the year, after optimization for harvesting regime and biomass concentration.

**3. TELCA 1.0 Model**

**3.1 Background**

**Summary:** *The TELCA (Technical Economic and Life Cycle Analysis) model is an excel-based platform that analyses complete algae-to-fuel production processes based on ~350 process variables and their interactions. It provides the unique capacity to analyse and optimise triple bottom line metrics in parallel (i.e. Economic: Internal Rate of Return; Social: Energy Return on Energy Invested - ERoEI; Environmental: Greenhouse Gas - GHG emissions reductions) and is designed to fast track systems optimisation, scale up and the development of profitable business models.*

**Previous Techno Economic Analyses (TEA)** of algae-to-fuel processes have reported diesel costs ranging from US$1.64 gal^-1^ (US$0.43 L^-1^) to US$30.00 gal^-1^ (US$7.93 L^-1^) ^26^. Three key factors that contribute to this variability include, production location, algae productivity assumptions, and variations in process equipment performance. Variations in process equipment performance has largely been addressed by work conducted by the National Renewable Energy Laboratory (NREL), Pacific Northwestern National Laboratory (PNNL) and Argonne National Laboratory (ANL) ^27,28^ who have provided a verifiable and consistent basis for the performance analysis of individual equipment components.

Much of the remaining variability of the modelled results is due to the use of differing production systems at different locations. For example, the optimum plant design and operating settings for a facility in the US Gulf Coast with associated weather conditions, tax regime and carbon pricing, is not the same as a corresponding facility being built in Haikou, China. The ultimate plant performance will consequently change as the physical plant variables are optimised to match the externally imposed commercial, social and environmental conditions.

**The TELCA platform** is designed to be able to address the above problems in two ways. First, TELCA’s single software platform eliminates the need to transfer data between software packages and so provides a seamless data structure that enables multifactorial analysis and optimisation of this complex space. Second, TELCA uses scalable input variables, with user-defined upper and lower range limits, rather than single fixed design parameter settings. The result is a model with over 350 process, design and economic input variables. Each one can be examined and optimised to achieve peak IRR, ERoEI and GHG performance, for a plant operating under a wide range of conditions.

**Model Structure:** TELCA was designed to be robust, user-friendly and flexible and so was developed using the widely adopted Microsoft Excel platform. A one-step fully automated model was generated with all operations including process simulation, equipment sizing, costing and financial modelling conducted on this single platform. This structure differs from most other TEA and LCA models that use multiple software platforms for process and cost estimation.


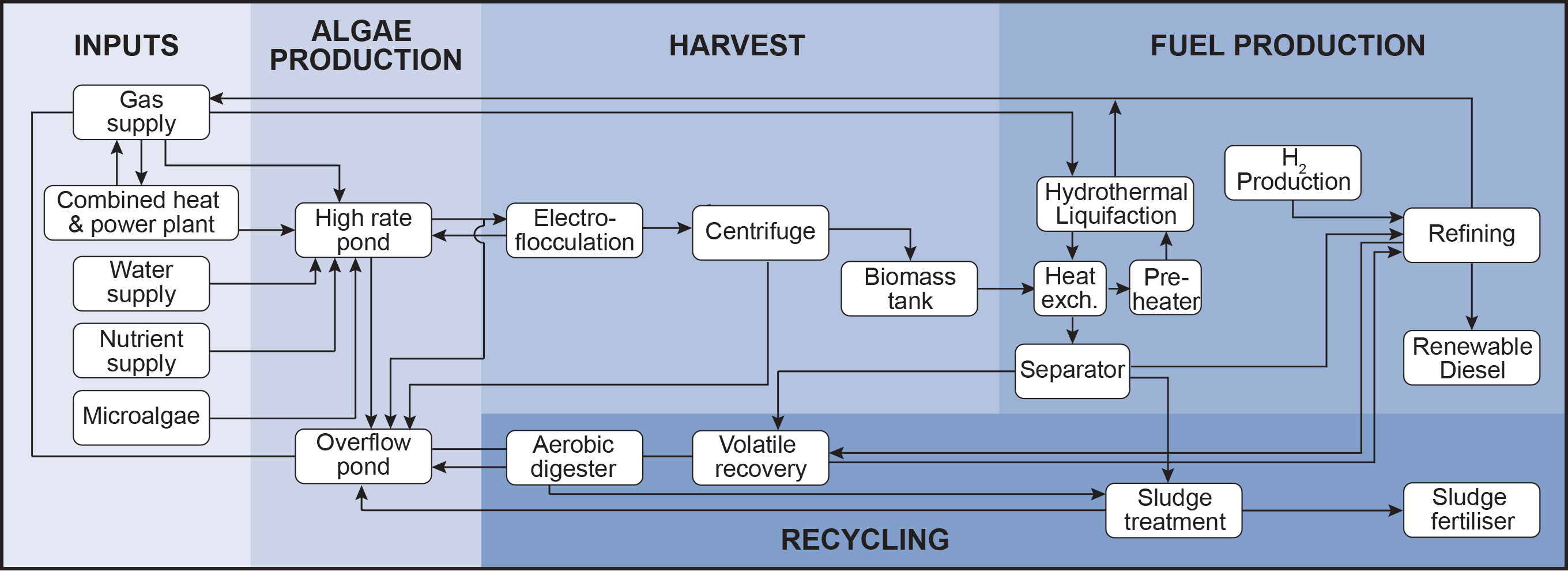


**Figure S18 Process System Overview.** Shows the relationships between major process elements.

The TELCA process flow was configured as a series of process modules detailed in Section.2, that the user can switch on or off, or substitute for other modules (e.g. electro-flocculation vs. chemical flocculation) to model a range of plant configurations. Each module contains scaled estimates of construction and operational costs generated from a central database of financial, energy and GHG intensity values. The system was configured with the necessary refining equipment to produce a full replacement product for conventional fossil oil derived diesel fuel.

For validation purposes the first system configuration selected for examination was designed to match the plant configuration reported by NREL/PNL/ANL ^29^, which is one of the most robust models available. Figure S18 provides a basic process overview of this configuration as it was initially examined. There were two exceptions to the direct matching of the models, and these were the use of:

**Anaerobic digestion** *in lieu* of catalytic hydrothermal gasification. This change reduces the MDSP by approximately 1.3% (see Section 4).

**A Proton Exchange Membrane (PEM**) electrolysis module was incorporated to supply hydrogen for diesel refining purposes. Electrolysis can be used to produce hydrogen, instead of the fossil fuels used as basis of the NREL/PNNL/ANL models. Through adoption of PV power this change enables reductions in GHG emissions important for the long-term sustainability of a renewable fuel production industry. This change increased the MDSP by approximately 1.4% (see Section.4).

After validation of the model against the published NREL/PNNL/ANL work (Section 4) a number of changes were made to the modelled process to improve economic, social and environmental performance. These changes were as follows:

**Seawater:** Converting the plant from the freshwater facility modelled by NREL/PNNL/ANL to a seawater-based operation. This change facilitates greater flexibility in operational locations and reduces potential competition for freshwater necessary for conventional agriculture.

**Electro-flocculation:** Use of electro-flocculation *in lieu* of chemical flocculation for the initial harvesting provides a marginally lower cost of harvesting to support increased adoption of renewable sources for production inputs.

**Working capital allowance:** The working capital allowance for operations post construction, was tied to OpEx rather than the initial plant CapEx. This change was made to reflect the fact that over the life of the production facility, the main financial risks are associated with the ongoing operational costs rather than the initial capital expenditure.

**Optimizing the high rate pond area** from 400 ha to 570 ha and configuring each farm as a stand-alone facility that includes processing and refining. TELCA optimization identified that a pond area of 570 ha was more efficient in terms of material distribution within the farm facility and linkage to CO_2_ sources.

**Pond size and shape:** TELCA was used to optimize individual pond size and shape to 1050 m x 45 m. This pond size corresponds to a pond water surface area of 4.27 ha rather than the 4-ha pond size used by NREL/PNNL/ANL.

**Operational day settings:** Operational days for the high-rate ponds were increased from 330 to 355 days per year, and new allowances were introduced to account for continuous ongoing maintenance of the ponds within the operational facility. Restricting operating periods to 330 days or less may be appropriate for some geographical locations (e.g. winter in temperate climates), but optimally selected sites (e.g. the subtropics) would be designed to operate all year round (365 days yr^-1^) with the understanding that there will be down time which is therefore separately accounted for. As algae growth can continue virtually independent of most equipment constraints, this level was set to 355 days per year. This figure is supported by the fact that the majority of pond equipment maintenance can be conducted at night without the need to schedule periods of no or limited productivity. In addition, the growth ponds provide a substantial buffer in overall production throughput i.e. short term outages (of a few hours) in refining will simply cause pond concentrations to increase marginally without substantially effecting the productivity of the ponds. The processing and refining equipment operational periods were set to a more conservative 345 days per year. This was set in consideration of the fact that, for the most part, the processing and refining portion of the facility will be operating well below rated capacity (less than 10% during winter in many locations).

This change was only applied after the validation comparisons between the TELCA and NREL/PNNL/ANL models. This adjustment allowed for *Scheduled Pond emptying* (2 days x 50% productivity as algae continue to grow), *Schedule Pond cleaning* (2 days x 0% productivity), *Schedule Pond filling* (2 days x 50% productivity as algae continue to grow), Unscheduled outages (8 days at 50% productivity). Furthermore, TELCA allows the user to specify operational algae growth downtime as well as operational processing and refining downtime.

**Corporate tax rate:** The corporate tax rate was changed from 35% to 21% to match current rates in the United States (2019).

The effects of these changes are detailed in Section 4.

**3.2 TELCA 1.0 Process Modules**

Individual spreadsheets were generated for each of the operational modules contained within the process. All inputs and outputs from these sheets were marshalled on a dedicated *Flowchart* module to streamline future addition and substitution of alternative process modules and variables. The full modules list of TELCA is provided in Table S19.

**Table S6.** TELCA Module List

| **Module Designation** | **Module Description** |
| --- | --- |
| ***Dashboard*** | Provides simple user interface to input variable parameters and test sensitivity |
| ***Summary*** | Provides detailed output of financial, energy and GHG results in summary and for individual components |
| ***Financials*** | Used to calculate IRR based on CapEx and OpEx inputs |
| ***Project Establishment*** | Used to calculate design, procurement and project management costs |
| ***Admin*** | Provides costs for non-process elements of construction and OpEx for indirect management and maintenance |
| ***Labour*** | Used to calculate all inclusive labour rates from base labour cost rates and other employment related parameters for both construction and operations |
| ***Light Calc*** | Analyses real location specific solar data and determines hourly direct and diffuse light conditions within the culture |
| ***Productivity*** | Calculates monthly/hourly productivity of the culture as a function of light |
| ***Flowchart*** | Used to marshal process flows between modules |
| ***Open HRP*** | Calculates production pond CapEx and OpEx costs |
| ***Electroflocculation*** | Calculates electro-flocculation CapEx and OpEx costs |
| ***Centrifuge*** | Calculates concentration centrifuge CapEx and OpEx costs |
| ***HTL*** | Calculates HTL CapEx and OpEx costs |
| ***Volatile Recovery*** | Calculates CapEx and OpEx costs for volatile recovery and separation units |
| ***Refining*** | Calculates refining unit CapEx and OpEx costs |
| ***CHP*** | Calculates CapEx and OpEx costs for combined heat and power facility |
| ***Hydrogen*** | Calculates CapEx and OpEx costs for electrolytic hydrogen production for refining input |
| ***Gas Supply*** | Calculates CapEx and OpEx costs for the collection, treatment and distribution of process gases |
| ***Water Supply*** | Calculates CapEx and OpEx costs for the treatment of process water |
| ***Digester*** | Calculates CapEx and OpEx costs for digester treatment of recycled material |
| ***Piping*** | Calculates CapEx and OpEx costs for inter-module piping |
| ***Power*** | Calculates CapEx and OpEx costs for inter-module power supply |
| ***Controls*** | Calculates CapEx and OpEx costs for plant control systems |
| ***PV Power*** | Enables the deployment of internally generated PV power in lieu of imported power supply |
| ***Nutrient Supply*** | Calculates CapEx and OpEx costs for the receive storage and distribution of solid nutrients |
| ***Nutrient Calculation*** | Provides data on mixed nutrient demand |
| ***Sludge Treatment*** | Calculates CapEx and OpEx costs for disposal of the digester waste stream |
| ***Carbon Balance*** | Used to ensure the inputs/outputs and circulation of elemental carbon flows throughout the *Flowchart* balance |
| ***Nitrogen Balance*** | Used to ensure the inputs/outputs and circulation of elemental nitrogen flows throughout the *Flowchart* balance |
| ***Fuel Security*** | Used to calculate total industry results for an Australian renewable fuel replacement program |

**Key operational variables:** Where possible, key operational variables were setup to enable user control and specification. This provision of user-defined variables enables a detailed analysis and optimisation of the process. Of over 350 defined variables available to the user, the most critical ones are listed in Appendix 1 together with units, set points and ranges, where applicable.

**Average annual and peak operating conditions:** Throughout the process model, two operating conditions were established: the *average annual operating* condition and the *peak operating* condition. The peak production rate for algae was used to size all downstream equipment throughout the plant. The peak operating condition was determined based on modelled average daily biomass production during the maximum production summer month. This relative productivity was calculated using the methodology detailed in Section.3 based on the assumption that light is the limiting growth factor.

**IRR/GHG/ERoEI Assessment:** From the process analysis, each item of equipment required for the particular module was sized and/or the quantity selected. The *capital* and *operational* costs (*financial, energy, GHG*) were then calculated based on this assessment of quantities together with the relative rate transferred from the internal database of material costs, labour costs, energy demand and GHG emissions.

**Equipment and materials:** For each item of equipment, industry standard techniques were used to establish the material quantity in a form related to the material cost database. These techniques included:

**Concrete:** In the case of concrete, 200 mm thick slabs with reinforcing beams were provided in all working areas, with 500 mm thick bases under pumps and equipment.

**Tank and pressure vessel steel thickness** was calculated based on 100MPa shell strength. A corrosion allowance of 3 mm was added to carbon steel equipment.

**Heat exchange equipment** was based on a heat exchange factor (k) of 1000 W.m^-2^.^o^C^-1^ for fluid to fluid exchange.

**The summary** **of the financial cost, energy cost and GHG emissions** for each module was then transferred to the *Summary* *worksheet* for overall project triple bottom line analysis.

**Task summaries:** In addition, the *$/Energy/GHG quantities*, the number of *labour hours* for operations was determined for each identified task and summed for the module. This summation, which is important to determine overall personnel levels and associated infrastructure, was also passed through to the *Summary* and *Admin* spreadsheets.

**Cost, Energy and GHG Commodities Database (Commodities)**

*Construction* and *operational activity hours* and *materials/commodity prices/energy content/GHG emissions* were calculated using a dedicated commodities database. These prices and hourly rates have been derived from a number of sources including:

**Piping activity hours** ^30^

**Material and equipment energy content and GHG emissions** ^31,32^.

**Experience:** Over 30 years of personal experience of the author (John Roles) covering construction and operations activities in the field of energy production.

**Other data** has been obtained through direct vendor quotations. Scaling of material supplies has been calculated using fixed and variable pricing assessments based on available vendor quotes for equipment where applicable, or the use of exponential scaling factors. All prices were assessed in Australian dollars to simplify the cost accounting processes. The final results have been expressed in United States Dollars for a more universal comparison to oil prices.

### **EROEI Calculations**

The boundary condition set for the calculation of ERoEI in the commodities database was generated with the EROI_2i_ condition as described in the work on energy investment return detailed in Murphy ^33^. All direct (excepting solar radiation) and indirect process energy flows, for construction and operations, were considered, but no allowance was made for energy related to labour inputs ^32,34-36^.

### **GHG Calculations**

GHG values were calculated based on *net emissions* from product use *and/or the embodied emissions* from supply of the commodities used in the construction and operations of the facility ^32^.

Current (2017) power generation in the southern United States has an average GHG emission profile of 0.72 T CO_2_eq.MWh^-1^. Similarly average GHG emission levels from the power sectors in Australia ^37^ and China ^38^ are 0.78 T CO_2_eq.MWh^-1^ and 0.92 T CO_2_eq.MWh^-1^ respectively. However, it is forecast that this profile will have to decrease significantly over time in line with COP21 commitments to achieve carbon neutrality by 2050. Any major execution of projects for the production of algae based liquid fuels will have operational periods that span over the period of GHG emission profile reduction. For the purpose of this analysis the value of external power generation GHG emissions was taken to be half the current southern US rate i.e. 0.36 T CO_2_eq.MWh^-1^.

Embodied emissions for a PV electricity generation plant will vary depending on the location of PV panel production and the respective emission profile in this location. To draw logical comparisons between operations based on external grid power to self-generated PV power, the embodied energy for the PV system was based on the same emission profile as indicated above. PV panels have an accepted ERoEI of approximately 7-8 ^39^, so the embodied emissions for the PV plant was taken to be 0.05 T CO_2_eq.MWh^-1^ capacity.

**3.3 TELCA 1.0 Module Descriptions**

The following descriptions define each of the modules used in the TELCA model. Several of the modules are designed to be replaceable components in the model (e.g. the *Open HRP* is designed as a growth unit that could be replaced with an alternate design for algae production such as a tubular bioreactor or flat panel bioreactor design). Where this is the case, the unit identifier (Growth Unit) has been provided after the module identifier.

The descriptions of the key process modules have been provided with a module summary table (Table S.3, Table S.4, Table S.5, Table S.7, Table S.8, Table S.9 and Table S.10) to help define the relative importance of each of the modules in terms of their financial *cost*, *energy cost* and *GHG emissions*. These tables are dependent on the particular configuration of over 350 model variables. The configuration used for the presentation here is the same as the one used to define Fig.1 Point 7 in the main paper. The figures relate to a 570ha pond area facility operating in the USA with an algae productivity of 25 g.m^-2^.d^-1^.

To help explain the model configuration, the modules are matched to individual spreadsheets and these spreadsheet names have been *Italicized in Orange.* Key factors and variables have been identified by *Italicized Blue.*

### **Dashboard spreadsheet**

The *Dashboard* spreadsheet is structured to provide a simple user interface. It allows the adjustment of key variables and the monitoring of impacts from these changes. The spreadsheet consists of five main panels:

**The variable input panel** lists the selected key input variables that were duplicated from key inputs in each of the process modules. The values entered against each of these key input variables in the *Dashboard* spreadsheet are transferred into the operational modules. Parallel to each of the variable entry cells, a second cell with default entry values is provided. This enables easy reset of the full set of *default values*. In addition, against each of the key variables *a high and low data* range is provided in separate cells.

**The results panel** was used to separately display:

**Financial results** including CapEx, OpEx and overall IRR

**Energy results** including construction energy input, operations energy input and product energy output and overall ERoEI

**GHG results** including construction emissions, operations emissions, product related emissions and overall GHG emission per MJ of product.

**Three sensitivity analysis panels** – one for each of *Financial, Energy* and *GHG settings*, providing a storage array to contain the results for each of these main outputs for a range of individual input variable settings.

A macro written into the *Dashboard* spreadsheet is compiled to adjust each of the variables in ten equal increments between the low and high ranges nominated (e.g. Tax Rate from 13% to 23%). The overall *IRR*, *ERoEI* and *GHG* emissions per MJ are written into the *sensitivity analysis panel*. These correspond to each of the incremental input variable settings. As the macro moved from one variable to the next the previously assessed variable is returned to the default (base case) value.

### **Summary spreadsheet**

The *Summary* spreadsheet is used to collect and collate the *financial, energy, GHG emissions* and *work hour* data from all of the other modules. It consists of three primary panels:

**Income panel**: where calculated product stream volumes are combined with applicable product selling rates to provide overall *yearly income*, *energy production* and *GHG absorption/emissions*. The user can turn on or off each of the income streams depending on the scenario being modelled.

**Cost summary panel:** which contains data transferred from each of the modules for overall construction and operations costs for each of *financial, energy, GHG emissions* and *work hours*. A toggle allows the user to switch modules *on* or *off* for inclusion or not, into the whole of plant costs.

**The relative value panel**: uses the overall plant ratio of construction to operational costs to assign *construction* and *operational values* to each of the modules. In this way, each of the modules can be assessed for its contribution to final financial outcomes. This relative value adjustment was only undertaken for the financial costs because of the future value discounting of monetary inputs. The calculation for energy and GHG analysis was conducted on the simpler basis than the financial analysis, in that no account was taken for difference in the values of the energy and emissions over time. Total annual energy inputs and emissions were equated to annual operational energy and emissions costs plus a straight-line proportion of the construction impact spread over the life of the plant in years.

### **Financials spreadsheet**

The *Financials* spreadsheet is used to assess the financial results associated with the particular plant configuration. Overall *plant construction* and *annual operational costs* were analysed with *tax, depreciation, loan, interest* and *equity* inputs to determine a project *IRR*. Yearly calculations of the financial position were used to determine the financial status rather than monthly calculations.

**Project Establishment module**

The *Project Establishment* spreadsheet is used to account for the project related costs not covered under individual process or infrastructure modules. These costs include:

***Land purchase costs***

***Design costs***

***Project approval costs*** including environment, community and development assessment

***Loan establishment*** and ***maintenance fees***

Most of these costs and fees were related to the total plant CapEx value. Design costs were assessed on a Section by Section basis (e.g. *mechanical, piping, controls*).

### **Admin module**

From a construction perspective the *Admin* spreadsheet covers all of the costs associated with *non-process areas of plant e.g. roads, fencing, drainage, security, maintenance and administration buildings* etc. Generally, *‘all in-plant’ roads* were designed to be compacted gravel, while asphalt was used for the *administration building access* and *car parking*. *Security fencing* was supplied around the entire facility.

Total operational work hours are transferred into the *Admin* spreadsheet, and used to calculate:

***Support staff requirements***

***Office and maintenance building sizes***

***Non-process power consumption***

***Maintenance equipment*** *and* ***vehicle requirements***

The *Admin* spreadsheet is also used to calculate the optimised solution for the *pond array*. Based on the user nominated values of *pond width, pond length* and *total pond area*, the spreadsheet calculates the array configuration to provide the closest approximation to a square array. It was assumed that all ponds would be oriented on a *north-south longitudinal axis*. The array was also provided with a central north-south spine, which included the *central processing facility, overflow ponds* and the *main piping corridor*. Ponds were arranged in rows either side of this spine with an east west access road between every pond. In this way, every pond could be provided with direct road access. See Figure S19 for a typical plant configuration.


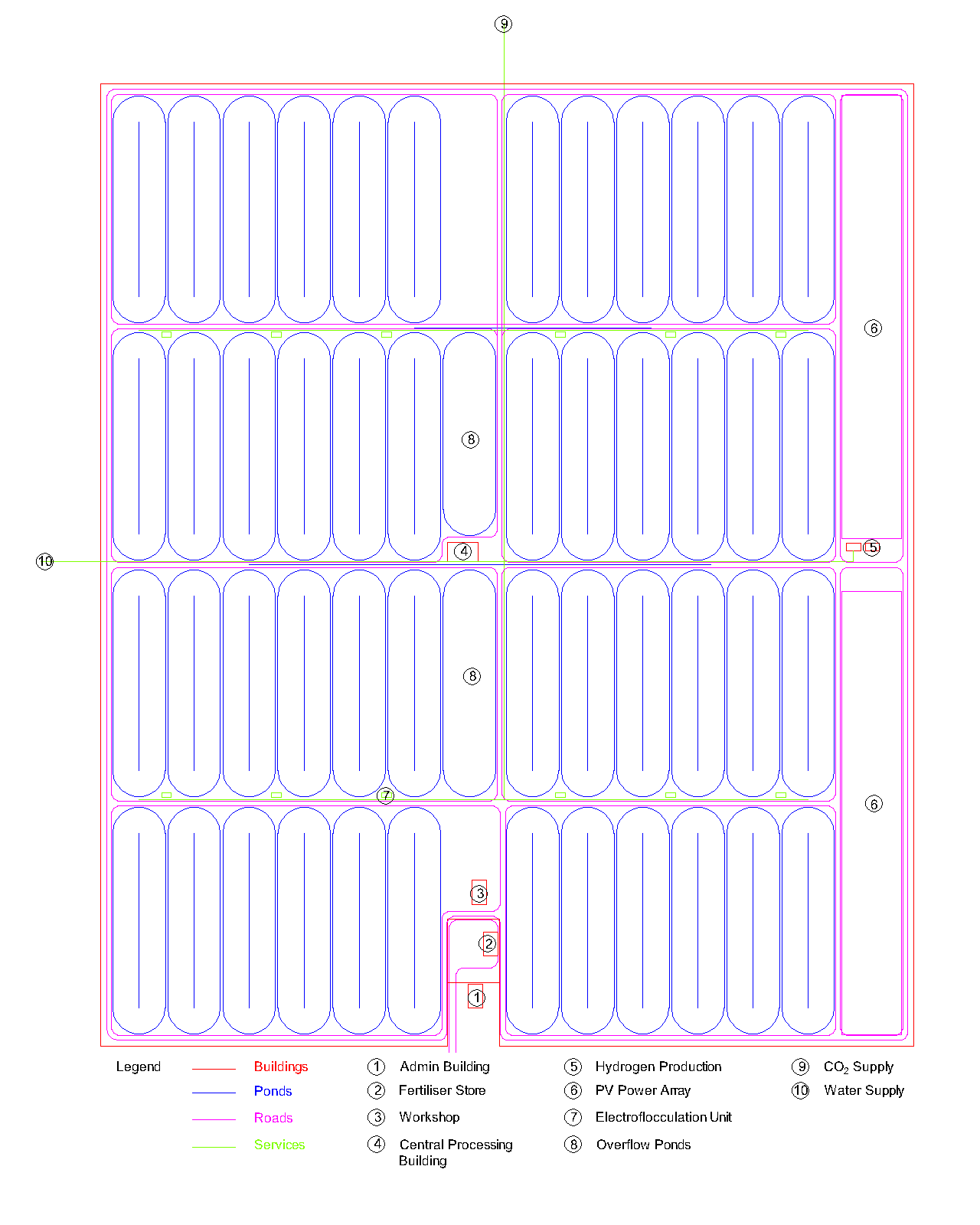


N

**Figure S19.** Plant Layout: Typical plant layout showing key buildings and components. Note that the length of ponds has been shortened to approximately 20% of optimal configurations to facilitate the display of the figure.

***Labour* module**

The *Labour* spreadsheet includes separate calculations for the overall applicable labour rates for *construction and operational activities*.

To calculate the construction labour rate, this spreadsheet was designed to incorporate all *supervision, non-productive time, overheads, tools,* *equipment* and *contractors profit* into a single hourly labour rate that was to be applied to direct productive work hour estimates for each construction task. This methodology is the same as that used across the construction industry in most developed countries. The inputs to this calculation include:

***Base work hour rate*** (rate for a skilled tradesperson)

***Loadings onto the base rate*** to cover unskilled workers, supervisors, administration personnel and management

***Crew build-up numbers*** to determine the proportions of these other personnel attributable to the numbers of tradespersons

***A labour efficiency factor*** to determine numbers of hours required to complete base estimated hours.

***Indirect hourly allowance*** to cover commencement, training, inductions and termination provisions

***Base working week*** duration

***Actual working week*** duration (a user defined variable)

***Overtime loading rate*** used as a multiplier for overtime rates applicable to hours exceeding the base working week

***Statutory on-costs*** used to provide for non-payroll costs for employing labour (e.g. workers’ insurance, holiday and annual leave pay)

***Consumable allowance*** used to make provision for hand tools, consumables and personal protective equipment

***Major equipment allowance*** used to make provision for major equipment items (e.g. cranes, welding machines, access equipment)

***Contractor margin*** to account for contractor profits and adverse weather and general risk

All of these items were used to calculate an overall *direct productive rate*. This rate was then transferred to the *Commodities* database for calculation of the costs associated with each construction task. A similar process was used to establish operational labour rates and the resulting *direct productive rate* was transferred to each of the operational modules for calculation of operation costs.

### **Light Calc module**

The *Light Calc* spreadsheet is used in TELCA to calculate irradiance at the pond surface and thus relative monthly productivity, based on latitude and month of the year. For each month of the year the midpoint day was selected for the calculation. Solar locations (Omega and Zenith) is calculated for each hour of the day. From this, the following equations were used to calculate hourly direct and diffuse irradiance:

$I=\pi\cdot Ho\cdot Kt\cdot0.0129\cdot(\frac{(cos\omega-cosS)}{sinS}-S\cdot cosS) \cdot(0.409+0.5016\cdot\sin\left( S-\frac{\pi}{3} \right)+0.1842\cdot\left( S-\frac{\pi}{3} \right)\cdot\cos\omega)$

$S={cos}^{-1}(-tan\delta.tan\emptyset)$

$\delta={23.45\cdot sin(\frac{360\cdot\left( 284+N \right)}{365})}$

$Id=I\cdot Hdf$

$Ib=I-Id$

Where:

*I*  = Total Irradiance

*Ho* = Extra-terrestrial solar radiation

*Kt =* Daily average clarity index

$\emptyset$ = Geographic latitude

*N =* Day of the year

$\omega$ = Angle corresponding to the solar hour [degrees]

*I_b_* = Incident direct beam radiation

*I_d_* = Incident diffuse radiation

*Hdf* = Light diffuse fraction

The *hourly direct* and *diffuse* irradiance factors for each month were transferred to the *Productivity* spreadsheet, together with a table of the angle of incidence for the direct light.

### **Productivity module**

The *Productivity* spreadsheet is used in TELCA to establish relative monthly productivities. Theoretical direct and diffuse irradiance figures transferred from the *Light Calc* spreadsheet, were initially corrected to reflect known irradiance numbers for Brisbane. This modification corrects for the difference between the theoretical clear sky condition and the actual ground irradiance taking into account the average of typical weather conditions. Note that while the Brisbane data was used universally in the TELCA analysis, this input only influences the difference factor between average and peak conditions. The yearly average productivity condition for TELCA is user defined.

From the irradiance number a three-dimensional array was generated reflecting light intensity for:

***Each hour of the day***

***The mid-month day*** of each month of the year

***At a range of 10 depth increments*** from the *pond surface* to the *pond bottom*.

The irradiance for each depth was calculated using the following equations:

I_z_ = I_col_ + I_dif_

I_col_ = I_b_ · secθ ·exp^(-dc·L)^

I_dif_ = I_d_ · 2 · exp^(-dd·L)^

d_c_ = E_a_· C_x_/cosθ

d_d_ = 2 · E_a_· C_x_

Where:

I_z_ = Local total photosynthetically active radiation (µmol m^-2^ s^-1^)

I_b_ = Incident direct beam radiation

I_d_ = Incident diffuse radiation

Θ = Angle of incidence for direct beam radiation (degrees)

E_a_ = Mass absorption coefficient of microalgae (m^2^ kg^-1^)

C_x_ = Dry biomass concentration (kg m^-3^)

L = Reactor pathlength i.e. depth (m)

From the irradiance at depth, a second *three-dimensional array* was generated reflecting the productivity at depth. This calculation was based on the following:

µ = µ_max_ · I_(z)_ / (K_s_ + I_(z)_ + I_(z)_^2^/K_i_) – (R_basal_ + R_light_)

Where:

µ_max_ = Theoretical maximum growth rate (h^-1^)

K_s_ = Half saturation irradiance (µmol m^-2^ s^-1^)

K_i_ = Irradiance of photoinhibition (µmol m^-2^ s^-1^)

R_basal_ = Basal respiration rate in the dark (h^-1^)

R_light_ = Constant for light-enhanced dark respiration (h^-1^)

From this *three-dimensional array* the productivity was averaged over the full depth of the culture. This yielded a *two-dimensional array* of productivity for *each hour of the average day, in each month of the year*.

In the initial TELCA analysis the productivity array was further reduced to an *average productivity for each month*. The proportional difference between *average yearly productivity* and *peak monthly productivity* was calculated, and this result was then transferred to the *Open HRP* spreadsheet.

### **Flowchart module**

The *Flowchart* spreadsheet is designed to provide a controlled routing of flow information between process modules. Each of the process modules represents a *process box* on the *Flowchart*. Each of these boxes is documented with an array of *input* and *output flow rates* representing the daily flow rates to and from the respective process units.

During the investigation a number of alternative *HTL* and *Harvesting* modules were investigated. This investigation was made considerably easier by simply switching *on* or *off* alternative modules and summing the outputs from them.

### **Open HRP (High-Rate Pond) – Growth Unit module**

Table S7 Open HRP module summary

| Impacts | Financial | Energy | GHGs |
| --- | --- | --- | --- |
| Construction | 50% | 82% | 66% |
| Operations | 13% | 5% | 5% |
| Combined | 37% | 14% | 11% |

**Description**: The *Open HRP* unit is designed to evaluate an array of raceway style, open, high-rate ponds. The array is configured to approximate, as accurately as possible, an overall square area using the user defined pond shape (*length & width*) and the user specified *total pond surface area*. Pond spacing and bund size (adjustable) are all automatically configured around the pond shape and depth. Perimeter and interstitial service roads are configured through the array to ensure accessibility.

The *main process building* and *overflow ponds* are arranged at the centre of the pond array. *Administration buildings* and *nutrient stores* are located adjacent to the pond array with road access to the *processing unit* at the centre. It has been assumed that *seawater access* for both water supply and discharge is available within a user defined radius on a second side of the array. Access to *CO_2_ flue gas* is available within a user-defined distance on a third side of the array.

Depending on the user-defined numbers, *initial dewatering* is carried out at nodes located throughout the array with *secondary separation* located in the main process building Figure S19. Services for *water collection* and *distribution, power distribution* and *control systems* are located underground. *Gas distribution piping* is arranged via above ground sleeper tracks.

All ponds including *overflow ponds* are lined with polyethylene liners. *Liner thickness* is a user defined variable.

*Pond circulation* is modelled to use conventional paddle wheels. *Circulation power demand* was calculated using Manning’s equations for open channel flow, at depth and velocity determined through user input.

h_t_ = h_f_ + h_k_

h_f_ = V^2^ * n^2^ * L / R_h_

R_h_ = A / P

h_k_ = 2 * V^2^/g

Where:

h_t_ = Total head loss (m)

h_k_ = Kinetic head loss (m)

h_f_ = Friction head (m)

V = Mixing velocity (m/s)

n = Friction coefficient of liner material

L = Average pond circulation path length (m)

R_h_ = Hydraulic radius (m)

A = Channel cross sectional area (m^2^)

P = Wetted channel perimeter (m)

***Paddlewheel efficiency*** was applied to the mixing energy demand derived from the total head loss. The *total head loss* around the pond was assessed against a user defined maximum allowable head loss across the individual paddle wheels to determine if multiple paddle wheel units are required on each pond ^40^.

***Primary CO_2_*** was supplied in the form of gas-fired power plant flue gas at nominal atmospheric pressure. The flue gas was supplemented with CO_2_ from the *Combined Heat and Power (CHP) plant* and other *refining processes*. CO_2_ distribution to the ponds was by compression, and the discharge into the ponds, via the dewater return lines to each pond. Pond CO_2_ concentration was maintained at a user defined level with a *CO_2_ Fixing Efficiency* also defined by the user.

***Algae Strain:*** To optimise access to the water supply and plant location options, the decision was made to select saline algal strains as the basis for the biomass production. Strain specific productivity data is not essential for the modelling process, as a user defined areal productivity value (g of biomass dry weight m^-2^ d^-1^) can be used for analysis.

***Saline concentration:*** Determination of pond salinity is a balance between nutrient losses through *blowdown* (i.e. discharge of waste to maintain chemical balances), optimising algal growth and reducing pond infection rates. *Top up water demand* is calculated by comparing *blowdown losses, process losses* and water changes through *rainfall* and *evaporation* determined from a *user defined weather array*. The pond *hyper-salinity level* is also a user defined variable.

***Pond shape dimensions, total pond area, pond depth, operating/harvest concentration*** are all user-defined variables that enable optimisation of the growth process. All other aspects of High-Rate Pond design are automatically adjusted to account for these variables.

***Growth rate:*** For the TELCA model a user-defined daily *Base Production Rate* was used. This average daily production rate was then modified by a factor transferred through from the *Productivity spreadsheet* to provide a *Peak Production Rate*.

***Overflow Ponds:*** Overflow ponds were provided to minimise environmental discharge from rainfall events and to facilitate stabilisation of nutrient recovery flows from downstream processes prior to recovery into the HRPs.

**Inputs**

***Dewater Return Water volumes*** for water, algae and dissolved nutrient

***Overflow Return Water volumes*** for water, algae and dissolved nutrient

***Makeup Water volumes*** for water and dissolved nutrient

***Nutrient additions***

***Rainfall***

***Biomass* *Base Productivity Rate*** and ***Peak production factor***

**Outputs**

***Harvest Water volumes*** for water, algae and dissolved nutrients for average and peak production conditions

***Evaporation losses:*** All equipment in the *Open HRP* module was sized to match the relevant peak harvest throughputs.

**User Defined Variables:**

***Pond Dimensions*** including ***Pond length****,* ***Pond width*** and ***Pond depth*** *(m)*

***Total Pond Area***

***Liner thickness*** and ***friction coefficient***

***Bund profile***

***Average excavation cut depth***

***Average algae concentration***

***Average yearly production rate***

***Pond CO_2_ concentration*** and ***CO_2_ Fixing Efficiency***

***Target salinity***

***Weather profile*** for ***sunlight, precipitation*** and ***evaporation rates***

***Paddlewheel mechanical efficiency, motor efficiency*** and ***maximum head depth***

***Overflow pond depth***

***Weather induced Overflow capacity***

### **Electro-flocculation – Harvest Unit module**

**Table S8.** Electro-flocculation module summary

| Impacts | Financial | Energy | GHGs |
| --- | --- | --- | --- |
| Construction | 2% | 2% | 2% |
| Operations | 10% | 5% | 5% |
| Combined | 5% | 5% | 5% |

**Description**: The primary biomass separation unit was configured to use *electro-flocculation* and settling to concentrate algae broth from ponds. The model is configured to locate these units centrally for a group of ponds. The *number of units* relative to the *number of ponds* is a user defined variable and can be optimised depending on the pond configuration. The *Electro-flocculation* *unit* consists of an aluminium plate flocculation tank in series with a continuous throughput *settling tank*. *Dewater Return Water* was returned directly to the *HRP units*. *Electrode size, thickness and spacing* was designed to optimise work associated with periodic replacement ^41^.

**Inputs**

***Harvest water*** volumes for water, algae and dissolved nutrient for average and peak conditions

**Outputs**

***Dewater Return*** volumes for water, algae and dissolved nutrients for average and peak conditions

***Concentrate volumes*** for water, algae and dissolved nutrients for average and peak conditions

All equipment in the *Electro-flocculation* module was sized to match the relevant peak harvest throughputs

**User Defined Variables**

***Number of units***

***Separator cut ratio***

***Separator efficiency***

***Separator residence time***

***Power Consumption Rate***

***Electrode Consumption Rate***

***Operating power*** and ***aluminium consumption rates*** were based on Lee et al. ^41^ and are listed in Appendix 1.

**Construction**

***Flocculation tank*** - fiberglass prefabrication

***Settling tank*** - concrete cast on site

### **Centrifuge – Dewatering Unit module**

**Table S9.** Dewatering Unit module summary

|  | Financial | Energy | GHGs |
| --- | --- | --- | --- |
| Construction | 4% | 0% | 1% |
| Operations | 12% | 2% | 2% |
| Combined | 7% | 2% | 2% |

**Description**: The *secondary biomass separation unit* is currently configured to use centrifugation to concentrate the algae concentrate flowing from the electro-flocculation units. The *Centrifuge* units are located at a processing facility central to the entire pond array. Unit size is determined by *volumetric throughput* and the user defined *minimum unit numbers* to ensure redundant capacity and operational security. Commercially available units (Alfa Laval) were evaluated with the objective of producing a slurry with 80% moisture content, considered optimal for *hydrothermal liquefaction*. A factored relationship between unit size and cost was established from quoted prices (Table S.6) and used to determine *power demand, area* and *cost* responses.

**Table S10.** Quoted Centrifuge Data

| **Capacity (L.hr^-1^)** | **Power (kW)** | **Footprint Area (m^2^)** | **Price (AUD$)** |
| --- | --- | --- | --- |
| 1000 | 5.9 | 1 | 68,000 |
| 2000 | 13.2 | 2 | 91,000 |
| 4000 | 18.5 | 3 | 205,000 |
| 20000 | 52 | 5 | 420,000 |

The *supernatant* from the centrifuge stage is returned to the growth ponds via the overflow ponds. It was assumed that the returned algae biomass would remain viable. *Access structures and building* sizes are proportioned to reflect equipment sizes and numbers.

**Inputs**

***Concentrate Water volumes*** for water, algae and dissolved nutrient for average and peak conditions

**Outputs:**

***Slurry Biomass volumes*** for water, algae and dissolved nutrients for average and peak conditions

***Filtrate volumes*** for water, algae and dissolved nutrients for average and peak conditions

All equipment in the *Centrifuge* module was sized to match the relevant peak harvest throughputs.

**User Defined Variables:**

***Minimum number of units***

***Separator cut ratio***

***Separator efficiency***

***Power Consumption Rate***

***Construction***

***Building*** – Masonry walls for noise containment with steel clad roof

### **HTL – Processing Unit module**

**Table S11.** Hydrothermal Liquefaction module summary

| Impacts | Financial | Energy | GHGs |
| --- | --- | --- | --- |
| Construction | 3% | 3% | 4% |
| Operations | 1% | 1% | 1% |
| Combined | 3% | 1% | 1% |

**Description:** Processing of slurry biomass is modelled on a continuous HTL process operating at 21.0 MPa and 340^o^C with three-phase separation of the products ^42^. Both *thermal and pressure energy recovery* is deployed across the *HTL* unit with primary thermal input derived from gas produced from the digester plant. Four product streams are generally associated with the post HTL three-phase separation. These are: a *gaseous stream* containing predominantly CO_2_; a *HTL Oil or Green Crude stream*; an *aqueous* stream with water soluble hydrocarbons and a *solid* stream (Figure S18). The flow rates for each of these streams was taken from the NREL/PNNL/ANL design case model. Several research groups have published papers discussing the processing methodology for the solid and aqueous product streams from the HTL process ^28,42-44^. The appropriate technology to maximise nutrient recovery for efficient algae growth and to extract further useful hydrocarbon products has not been fully resolved. It is appropriate, however, that some allowances for this process be considered in the model. These have been detailed in the modules *Anaerobic Digestion* and *Volatile Recovery*.

***Reactor heat input*** is obtained through emersion of the active ends of the plug flow reactor tubes into a molten salt bath heated via furnace firing using available combustion gas.

**Inputs**

***Slurry biomass mass flow*** for water, algae and dissolved nutrient for average and peak conditions

***Combustion gas mass flow*** for methane and CO_2_

**Outputs** (average and peak conditions for all)

***Gas phase mass flow*** for CO_2_ and methane

***Liquid phase kerogen mass flow*** for hydrocarbons, water and ash

***Liquid phase aqueous mass fl*ow** for water, volatile hydrocarbons, dissolved hydrocarbons, ash, nitrogen and CO_2_

***Solid phase mass flow*** for water, ash and solid hydrocarbons

***Combustion products mass flow* of CO_2_ and carry gas**

All equipment in the *HTL* module was sized to match the relevant peak harvest throughputs.

**User Defined Variables**

***Hydrocarbon conversion rates*** for kerogen, aqueous volatile and aqueous non-volatile flows

***Biomass feed tank residence time***

***Separator cut ratio***

***Separator efficiency***

***Power Consumption Rate***

**Construction**

***Building*** – Masonry walls for noise containment with steel clad roof sized to match equipment sizing

***Access platforms*** – Steel sized to match equipment size

### **Refining module**

**Table S12.** Refining module summary

|  | Financial | Energy | GHGs |
| --- | --- | --- | --- |
| Construction | 11% | 4% | 8% |
| Operations | 34% | 46% | 50% |
| Combined | 19% | 40% | 46% |

**Description**: *Refining* of the HTL product is modelled on *hydrotreatment* of the volatile *hydrocarbon product* stream followed by *fractionation*. Fifty percent of the hydrotreater product was further treated using *catalytic hydrocracking*. *Hydrogen consumption rates* were assumed to be 4% w/w and 2% w/w for the *Hydrotreater (HT)* and *Hydrocracker (HC)* respectively.

**Inputs**

***HTL Oil mass flow*** for hydrocarbon and water

***Volatile Recovery Liquids hydrocarbon content mass flow***

***Hydrogen mass flow***

**Outputs**

***Refined Renewable Diesel mass flow***

***Water mass flow***

***CO_2_ mass flow***

All equipment in the *Refining* module was sized to match the relevant peak throughput condition.

**User Defined Variables**

***Hydrocarbon portion of HTL Oil***

***Hydrotreater conversion rates*** for H_2_ consumption, hydrocarbon product, CO_2_ and water production

***Proportion of Hydrotreater product requiring hydrocracking***

***Hydrocracker conversion rates*** for H_2_ consumption, hydrocarbon product, CO_2_ and water production

***Pump efficiencies*** for low pressure feed, high pressure feed, HT recirculation, Fractionator recirculation and HC recirculation

***Operating temperatures*** for HT and HC

***Heat transfer rates*** for heat exchangers for product and water flows

**Construction**

***Building*** – Masonry walls for noise containment with steel clad roof sized to match equipment sizing

Equipment and piping generally alloy steel.

### **CHP (Combined Heat and Power) Plant module**

**Description**: The *CHP plant* is designed to utilise methane production from the *Anaerobic* *Digestion* facility and the *HTL* facility for production of *electrical power*. The plant utilises reciprocating *gas engines* sized to accommodate the *peak residual gas production* after accounting for the thermal gas supply to the *HTL* facility. *Flue gas* from the *CHP* facility is utilised for low level heating requirements (e.g. *anaerobic digester heating*), prior to passing the *flue gas* back to the *gas supply* module where it is used to supplement *CO_2_ supply* to the *high rate ponds*.

The unit as modelled is configured to operate on a *24-hour duty cycle*. In practice, it is probable that the unit is switched off during periods of high output from the *PV array*. In this way gas can be conserved for night operation and the plant operate generally without grid support.

**Inputs**

***Combustion gas mass flow*** including methane and carry gas components, for average and peak conditions.

**Outputs**

***Flue gas mass flow*** including CO_2_ and carry gas components, for average and peak conditions

***Power generation***

All equipment in the *CHP* module was sized to match the relevant peak throughput condition.

**User Defined Variables**

***Number of engine units***

***Generator heat rate*** (MJ.MWhr^-1^)

### **Hydrogen Supply**

**Description**: The *Hydrogen* *supply* module is designed to provide a continuous supply of hydrogen for use in the *refining plant (hydrotreating* and *hydrocracking***)**. The unit is based on the production of hydrogen using *electrolytic proton exchange membrane technology*. The unit size was configured to provide peak daily production requirements within an eight-hour operating window, designed to match peak PV output. *Building size* and *water efficiency* have been derived from supplier commercial literature. Loss of water occurs as a result of filtration and purification losses. *Hydrogen* is compressed through a *two stage compression unit* with *intercooling* and stored in *horizontal bullet type tanks*.

**Inputs**

***Water supply rate***

***Power Supply***

**Outputs**

***Hydrogen supply rate***

All equipment in the *Hydrogen* Supply module was sized to match the relevant peak throughput condition.

**User Defined Variables**

***Electrolytic power consumption rate*** kW.kg^-1^

***Water efficiency***

***Compressor efficiency***

***Storage pressure*** (kPa)

***Storage capacity based on peak demand*** (hr)

### **Utilities**

**Table S13.** Utilities Units module summary

|  | Financial | Energy | GHGs |
| --- | --- | --- | --- |
| Construction | 29% | 9% | 19% |
| Operations | 6% | 12% | 13% |
| Combined | 20% | 12% | 14% |

**Description:** Several utility modules are configured to support process activities in the plant. For the purpose of the above module summary table the following units have been amalgamated: *Gas Supply, Water Supply, Anaerobic Digestion, Piping, Power Supply* and *Controls*. All linear infrastructure such as *distribution and collection piping, power distribution* and *control systems* have been included into the *Utilities category*. As there were no significant processes occurring within these modules, isolated variable analyses (Section.4) were not undertaken.

### **Gas Supply module**

**Description**: The *Gas Supply* module balances methane and CO_2_ supply and demand from the various process sources. *Methane supply* from the *Anaerobic Digester* and *HTL* is matched to *heating gas demand* from the *HTL*, with any remainder channelled through to the Combined Heat and Power (*CHP*) facility. *CO_2_ supply* is assessed, and then compared with the demand from the *High Rate Ponds* with the additional demand being supplied from the external source.

The external *CO_2_ supply source location* is a user defined variable but for the purposes of the subsequent analysis, it was assumed to be 50m outside the plant boundary. This supply source was modelled on the *exhaust flue gas* from a *combined cycle gas fired power station*. Conventional *exhaust gas supply* for this type of source is at a temperature of approximately 120^o^C. To provide acceptable temperatures for the reticulation piping in Fiberglass Reinforced Plastic (FRP) material, the gas temperature must be reduced to around 60^o^C. As a result the main supply source was fitted with a *gas quencher*. This equipment is typically used for particulate suppression in biomass fired boilers. The unit consists of a chamber fitted with water mist sprays, used to deliver an evaporative cooling effect to the gas flow. This unit is then followed by a knockout drum and mist eliminator. Centrifugal fans provide the distribution pressure requirements derived from the *Piping* spreadsheet.

**Inputs**

***CO_2_ External Supply mass flow*** for CO_2_ and carry gas

***Volatile Recovery unit mass flow*** for CO_2_

***Anaerobic Digester mass flow*** for methane and CO_2_

***HTL Gas Stream mass flow*** for methane and CO_2_

***HTL Firing Gas stream flow*** for CO_2_ and carry gas

***CHP Firing Gas Stream*** for CO_2_ and carry gas

***Refining Plant for CO_2_***

***CO_2_ delivery pressure***

**Outputs**

***High Rate Ponds HRP gas supply mass flow*** for CO_2_ and carry gas

***HTL Heating Gas supply mass flow*** for methane, CO_2_ and carry gas

***CHP Gas supply mass flow*** for methane, CO_2_ and carry gas

All equipment was sized to match the relevant peak throughput condition.

**User Defined Variables**

***External CO_2_ supply*** conditions including *pressure, temperature* and *concentration*

***CO_2_ delivery*** conditions including *pressure* and *temperature*

***Methane delivery*** *pressure* and *temperature*

### **Water Supply module**

**Description**: The *Water Supply* module provides makeup water from a seawater intake into the plant via the *overflow ponds*. *Filtration* and *tank storage* is provided in the supply circuit with capacity based on peak makeup demand. The location of the *water supply point* is a user defined variable but for the purpose of subsequent analysis was assumed to be 50m outside the plant boundary. *Makeup water* delivery was directly to each of the ponds. It is of note that it might be possible to deliver makeup water into the *centrifuge return* *streams* thus eliminating distribution pipework. To be conservative, this position was not adopted due to uncertainty over control implications.

**Inputs**

***Intake water flow*** with associated natural seawater nutrient levels

**Outputs**

***Makeup water flow*** with associated natural seawater nutrient levels

All equipment in the *Water Supply* module was sized to match the relevant peak throughput condition.

**User Defined Variables**

***Seawater supply conditions*** including salinity

***Storage capacity*** (hours)

***Filter backwash rate***

### **Anaerobic Digester module**

**Description:** *Waste solids* and *liquids* from both the *HTL* and the *Volatile Recovery* unit are passed into an *Anaerobic Digester* to generate usable methane and to break down the products into water and biologically available nutrients. These are passed back to the high-rate growth ponds via the overflow pond. Unpublished data from personal discussions with researchers using *AD for digestions of HTL products* (including saline based systems) have indicated this process is possible using appropriate selection of *AD biological strains*. The system settings are defined in Appendix 1

**Inputs**

***Volatile Recovery waste mass flow*** for water, volatile hydrocarbons, non-volatile hydrocarbons, ash, nitrogen compounds and dissolved CO_2_

**Outputs**

***Anaerobic Digester Gas mass flow*** for methane and CO_2_

***Anaerobic Digester Liquids mass flow*** for water, nitrogen, soluble carbon products and dissolved CO_2_

***Anaerobic Digester sludge production including mass flow*** for ash, hydrocarbons, water and nitrogen products

All equipment in the *Anaerobic Digester* module was sized to match the relevant peak throughput condition.

**User Defined Variables**

***Anaerobic Digester residence time***

***Liquid/Gas ratio***

***Mixer demand power rate***

***Mixer reserve capacity***

***Anaerobic Digester Gas production rates for CO_2_ and CH_4_***

***Soluble Hydrocarbon production rates***

***Sludge moisture content***

### **Piping module**

**Description:** Pipework in the *Piping* module is configured based on the plant layout determined in the *Admin* spreadsheet. *Run lengths* were calculated with appropriate allowances for bends (one change of direction for every 4 metres of in-building pipework) and differences in elevation. Each typical piping run was analysed for *pipe capacity* and *pumping requirements*.

Generally, High Density Polyethylene (HDPE) piping was used for all liquid lines except where temperature considerations required the use of either carbon or alloy steel. *Liquid flow velocities* were set to 2 m.s^-1^ except for high viscosity flows of algae slurry post centrifugation. *CO_2_ supply* through flue gas was delivered throughout using FRP piping for larger diameters and HDPE piping for smaller diameters, with maximum flow rates of 20 m.s^-1^.

***Head loss calculation*** for liquid flows was performed in accordance with the Hazen-Williams formula ^45^:

H_f_ = 10.67 Q^1.852^/(C^1.852^.d^4.8704^)

Where:

H_f_ = Head loss (m)

Q = Volumetric flow rate (m.s^-1^)

C = Pipe roughness coefficient (generally 140 for HDPE piping)

d = Inside pipe diameter (m)

The following pump selection characteristics were used:

***Pump efficiency*** 70%

***Motor efficiency*** 90%

***Pump capacity*** based on peak flow conditions with maximum head differential

***Motor size*** based on pump capacity plus 25%

***Power consumption***: For the purposes of calculating power consumption the average operating condition was selected with normal operating head differentials.

***Pipe runs:*** Each of the pipe runs was assessed for application of appropriate supports. Large diameter *flue gas lines* were supported on sleepers with 6m spacings. Allowance was made for most long run liquid lines to be “direct buried”.

***Valves:*** Each of the typical piping runs was assessed for control requirements to determine quantities of valving including *motor operated valves, control valves, check valves* and general *shutoff valves.*

All of the cost quantification of *equipment, piping, pipe supports,* and *pipe welding* was assessed through rates applied from the ***Commodities* database**.

### **Power Supply module**

***Major power supply circuits:*** Each major power supply circuit was analysed to ensure that the correct cable sizes were selected. The spreadsheet is configured to select appropriate *circuit voltage* based on *peak demand*. *Circuit run lengths* were based on the plant layout determined in the ***Admin* spreadsheet**.

***Power demands*** were selected to service motor sizes identified through the process assessments in the *Piping module, Admin module* or other *Process modules*. Peak process conditions were used in the selection of motor sizes in these modules. The *Power Supply module* assembled each of these *motor loads* and applied appropriate *motor starting factors* to determine motor starting loads and currents. It was assumed that *soft starters* would be installed on all drives greater than 100kW. *Cable selection* was made through a lookup table of allowable current for different cable sizes in different support configurations i.e. conduit, cable tray or direct buried.

***Load assessment:*** For each load, an assessment was made concerning daily operating hours. *Voltage drop* and *power losses* were calculated based on program selected cable sizes. Power losses were applied in conjunction with operating hours to determine *power loss consumption*. *Motor Control Centre* (MCC) units were provided at each motor drive or group of motor drives.

***Transformers*** were selected for voltage change locations based on amalgamated loads including cable losses. A 25% contingency was allowed above the calculated loads.

***Central process plant cables:*** All central process plant cables were supported in cable trays or conduit. It was assumed that long in-plant runs would be direct buried.

***Flue gas:*** In the case of the flue gas supply, *compressors* and a separate *local power supply* was modelled.

***Lighting:*** Appropriate allowances were made for *central process plant lighting, harvesting equipment lighting* and *administration building lighting*. No allowance was made for *general access road lighting* or *perimeter lighting*.

***Cabling:*** From all of the cable runs calculated, the number of terminations was established. All of the material quantities for equipment, *cables, cable supports,* and *terminations* were assessed through rates applied from the *Commodities* database.

### **Controls module**

In the *Controls module*, each of the *process circuits* as well as the *process equipment* identified in the other process modules was assessed for control system requirements. In general, the control system requirements were assessed in relation to the following instrumentation groups:

***Level switches***

***Level controllers***

***Temperature controllers***

***Flow controllers***

***Pressure switches***

***Pressure Controllers***

***pH controllers***

***Control Valves*** (included in the Piping spreadsheet)

**Double redundancy** was generally utilised. In critical high-risk areas of the HTL module and refining module, **triple redundancy**, was employed in calculating instrument numbers. No specialisation of instrument selection was made to account for different service conditions namely *pressure* and *temperature*. Variations in operating conditions were accounted for in the average pricing of instrumentation.

***Circuit run lengths*** were based on the plant layout determined in the *Admin* spreadsheet and allowances were made for either direct buried or conduit support of the cable runs.

***Programmable Logic Controllers (PLCs)*** were provided at each of the major *process units* and the *harvesting units*. It was assumed that process control for the ponds would be handled in groups at the respective harvesting unit. PLC size selection was made based on total I/O count with a 50% redundancy level built into the assessment.

All of the material quantities for *equipment, cables, cable supports* and *terminations* were assessed through rates applied from the *Commodities* database.

**Nutrient Supply module**

**Table S14.** Nutrient supply module summary

| Impacts | Financial | Energy | GHGs |
| --- | --- | --- | --- |
| Construction | 0% | 0% | 0% |
| Operations | 24% | 31% | 23% |
| Combined | 9% | 27% | 21% |

*Total GHG emission mass flow from plant operations is negative and so the base for contribution calculation cannot be used.

**Description**: The nutrient supply module accounts for the materials and equipment required to *receive, store, dose* and *supply* required supplementary nutrients into the *Open HRP* (High Rate Ponds) ^46^. It was decided to locate the *receiving, blending and dosing facility* at the perimeter of the pond array rather than at the central processing facility. This decision was to prevent the need for heavy trucking to be transiting through the plant.

The facility consisted of a series of *ground storage bins* located within a *dust containment building*. Truck deliveries of nutrients would be discharged into these bins. From the bins a front-end loader would be used to transfer the fertilisers into a *mixing hopper* equipped with *load cells*. Water diverted from the *Centrifuge Return* water was directed into the mixing bin where granular fertilisers would be liquefied before returning them to the ponds via the *Centrifuge Return water*.

*Volumetric storage* requirements were calculated based on *fertiliser throughput* and appropriate *individual delivery volumes* for each fertiliser. The *storage volume buffer*, the volume above the delivery load, was calculated to be a *minimum number of days* or a *percentage* of the *individual delivery volume*. Both of these variables were user defined.

*Mixing hopper volume* was calculated based on a user defined *number of hopper loads per day* at peak demand.

*Lower volume nutrients* with less than 100 kg throughput per day would be dosed directly by bag (gradually to protect against local overdosing), into the ponds. *Labour requirements* for this activity were included in the operational costs.

**Inputs**

***Fertiliser demand*** for average and peak productivity conditions for each of the fertilisers was considered

**Outputs**

*Construction* and *operational* costs only

All equipment was sized to match the relevant peak throughput condition.

**User Defined Variables**

***Storage buffer volume***

***Storage buffer resupply period***

***Resupply delivery volumes***

***Supply storage types for different fertilisers***

### **Nutrient Calculation module**

The *Nutrient Calculation* module is designed to facilitate the supply of a range of different fertiliser chemicals to satisfy the nutrient demands of algae growth. The module is built around the Redfield ratio ^47,48^ of elemental constituents of algal biomass, but specific nutrient blends can be defined by the user.

**Description**: Based on *net biomass production*, an *elemental demand* is calculated in the *Open HRP* module and forwarded via the *Flowchart* module to the *Nutrient Calculation* module. Currently, the following elements are considered for the purposes of nutrient calculations but more can be added as required: *Nitrogen, Sodium, Magnesium, Phosphorous, Sulphur, Chlorine, Potassium, Calcium, Iron, Zinc, Manganese, Boron, Molybdenum* and *Copper.*

The *Nutrient Calculation* module contains six main panels:

***Fertiliser Component breakdown*** that details the elemental composition of 15 commonly available fertilisers

***Fertiliser calculation sequence table*** documents the priority sequence that fertilisers are to be “called” under four different sequence regimes.

**A quantity calculation table** for each of the four different regimes. Each of the regimes is designated with the primary nitrogen supplying fertiliser used within the regime. These were *Ammonium Sulphate, Urea, Calcium Nitrate* and *Potassium Nitrate.*

Depending on the user-selected regime, the spreadsheet applies *elemental quantities* to the elemental demand in the sequence determined by the *sequence panel*, until each of the required elemental demands is fully satisfied.

**Inputs**

***Elemental Demand*** for each of the key elements Nitrogen, Phosphorus, Potassium, Calcium, Magnesium, Sulphur, Sodium, Chlorine, Iron, Manganese, Zinc, Copper, Boron and Molybdenum based on average productivity.

***Selected primary nitrogen fertiliser*** e.g. urea.

**Outputs**

***Demand for various fertilisers***

**User Defined Variables**

***Selection of primary nitrogen supply fertiliser*.**

***Potential fertilisers*** and their respective *elemental mass compositions*.

***Fertiliser application sequence*** for each of four regime alternatives.

### **Carbon Balance module**

**Description**: The *Flowchart* provides an accounting balance for *water flows, nutrient flows* around the entire system and *biomass flows* between modules for the first portion of the process. As the process moves through the *HTL* unit and to the *Refining unit*, biomass ceases to be a relevant measure. During these stages, the process must take account of *elemental flows*. This analysis of elemental flows is particularly critical for carbon accounting. The *carbon balance module* was created to account for carbon flows around the entire system including biomass movements in the first portion.

Each process flow is assessed for *flow rate*. These flows mimic the flows nominated in the *Flowchart* module. Flows are then analysed for *carbon content* and a *carbon mass balance* determined for each of the process units. The spreadsheet flags any discrepancies in balance.

**Inputs**

***Process flows from Flowchart module***

***Carbon content of each process flow material***

**Outputs**

This module is used as a validation tool.

### **Nitrogen Balance module**

**Description**: The *Flowchart* provides an accounting balance for *water flows, nutrient flows* around the entire system and *biomass flows* between modules for the first portion of the process. As the process moves through the *HTL* unit and to the *Refining* unit, biomass ceases to be a relevant measure. During these stages the process must take account of elemental flows. This analysis of elemental flows is particularly critical for *nitrogen accounting*. The nitrogen balance module was created to account for *nitrogen flows* around the entire system including biomass movements in the first portion.

Each process flow is assessed for *flow rate*. These flows mimic the flows nominated in the *Flowchart* module. Flows are then analysed for *nitrogen content* and a *nitrogen mass balance* determined for each of the process units. The spreadsheet flags any discrepancies in balance.

**Inputs**

***Process flows*** *from Flowchart module*

***Nitrogen content*** *of each process flow material*

**Outputs**

This module is used as a validation tool.

**4. TELCA 1.0 Model Validation**

Validating the modelling of a complex process such as the microalgal renewable diesel production in Excel (~350 variables), requires thorough testing to eliminate all errors and, ensure the reliability of all programed results. To achieve this TELCA was subjected to robust testing at three levels: at the *module level, full system level* and via *benchmarking* TELCA against the best ‘*international standard’* models.

**Individual module testing:** Each of the individual modules was rigorously tested in isolation. To achieve this a range of input variables were adjusted and the correct output confirmed.

**Process testing:** Next the individual modules were connected into a series of subprocesses and finally the full algae-to-renewable diesel production process. To test the performance of the entire system sensitivity, analysis was conducted on financial results from key variables in each of the process modules. These sensitivity graphs (e.g. Figures S17 to S20) show that the model performed as expected. The same approach was also taken to analyse the greenhouse gas emissions and energy return on energy invested variables (e.g. Figures S.4 to S.8).

**Validating against independent models:** Next the fully validated TELCA model was validated against a broad range of independent techno-economic and life-cycle analyses (Figure S26). Of these, we consider the NREL model (Davis 2011, Figure S26) to be the most comprehensive. Given the complexity of our TELCA model and that of the NREL model, and the fact that when set to the same production conditions they yielded a mean diesel selling Price within 1% percent of one another, we conclude that the NREL and TELCA models independently validate each other. In summary, we believe that this analysis not only confirms robustness of TELCA but also of the NREL model.

**Industry validation:** Next we extensively validated our model by subjecting it to industry scrutiny as well as a broad range of technology specialists. We also built a $3 million pilot plant and conducted extensive experimentation to support our systems settings. John Roles first author on this paper also brings 30 years of energy infrastructure experience, which has allowed him to incorporate detailed specific unit costs and cost estimation techniques identical to those used by process facility constructors and operators. The off-the-shelf cost estimation packages used in other models are notoriously inaccurate when applied to non-conventional applications such as the development of entirely new process technologies.

**Module level testing**

**Formula checking:** As each module was created or modified, a quality checking process was implemented to verify that the formulae in each module and copying process to subsequent modules functioned correctly. In the initial set up of the model, fixed inputs were entered into each module, to confirm that the correct module outputs were calculated. This was a critical first validation step, as in the full process model, the output of a given module often provided inputs into subsequent modules. These initial input values were progressively replaced within each of the process modules by detailed formula-based calculations and tables. Each time this replacement was carried out, all high-level output results at a *module level*, and at the *full system level*, were thoroughly checked for consistency and accuracy. Any unexpected variations to these high-level results was investigated to identify the cause, and if necessary corrected until all any erroneous linkages had been corrected.

**Data entry quality control:** TELCA was designed so that all key variables were entered centrally from the *Dashboard* module and distributed to all respective modules from that location. The advantage of this approach is that variable entries can be easily and robustly checked in the *Dashboard module*. This eliminates the unreliable alternative to this approach; the entry of each variable into each relevant module field, which is likely to result in entry errors. By adjusting each variable setting (e.g. for a low to a high value) rapid and comprehensively testing and quality control the system response was facilitated and conducted.

Where the *Dashboard* module facilitated a central location for variable entry into the model, the Finance module and the *Flowchart* central collection points for outputs from the model. This meant that variable inputs could be checked for all critical responses without searching through individual spreadsheets.

**Response curves:** The third *module* level quality control conducted involved the generation and analysis of response curves. Here, each module was isolated from all external inputs (except cost database references) before key variable inputs were changed across a 100-fold range (0.1 times, 1 times and 10 times the base case value) to ensure that outputs responded appropriately (Figure S20 to S22). This response curve testing was not relevant for modules without process elements; consequently, it was conducted only on the modules with complex inter-related process elements. By isolating the module, complex interactions could be eliminated, and the individual module response tested. The response curves were generated for *financial* and *energy* results. *GHG emissions* were not tested as the results for GHG emissions are controlled by the above *energy* calculation drivers. The *CapEx* and *OpEx* results were analyzed independently as these demonstrate more clearly the correct operability of the module calculations. The following modules were tested in this way: *Open HRP module* (Figure S20)*, Electro-flocculation module* (Figure S21)***,*** *Centrifuge module* (Figure S22)***,*** *HTL module* (Figure S23) *Refining module* (Figure S24). All the curves displayed expected responses, thereby validating the performance of each module.

**Figure S20.** Open HRP (High-Rate Pond) Module Validation. Module validation graphs: Isolated single variable sensitivity analysis based on the use of 0.1(1), 1(10) and 10(100) times the base case values for pond area.

**Figure S21.** Electro-flocculation Module Validation. Module validation graphs: Isolated single variable sensitivity analysis based on the use of 0.1(1), 1(10) and 10(100) times the base case values for Harvest Water flowrate.

**Figure S22.** Centrifuge Unit Module Validation. Module validation graphs: Isolated single variable sensitivity analysis based on the use of 0.1(1), 1(10) and 10(100) times the base case values for Concentrate Water flowrate.

**Figure S23.** HTL Module Validation. Module validation graphs: Isolated single variable sensitivity analysis based on the use of 0.1(1), 1(10) and 10(100) times the base case values for biomass Slurry flowrate.

**Figure S24.** Refining Module Validation. Module validation graphs: Isolated single variable sensitivity analysis based on the use of 0.1(1), 1(10) and 10(100) times the base case values for HTL Oil flowrate.

### **System Level Testing**

With testing of the individual modules complete it was important to validate the linking and total system response. Upon the completion and linking of each process module, a system level response test was performed. These tests involved the generation and review of sensitivity graphs covering the three primary output variables (**financial**, **energy** and **GHG emissions**). Using the input variable ranges in the ***Dashboard*** over 50 input key variables were tested for consistent and contiguous responses over the ranges determine appropriate for each specific variable. Figure S25 provides examples of the types of sensitivity graphs produced.


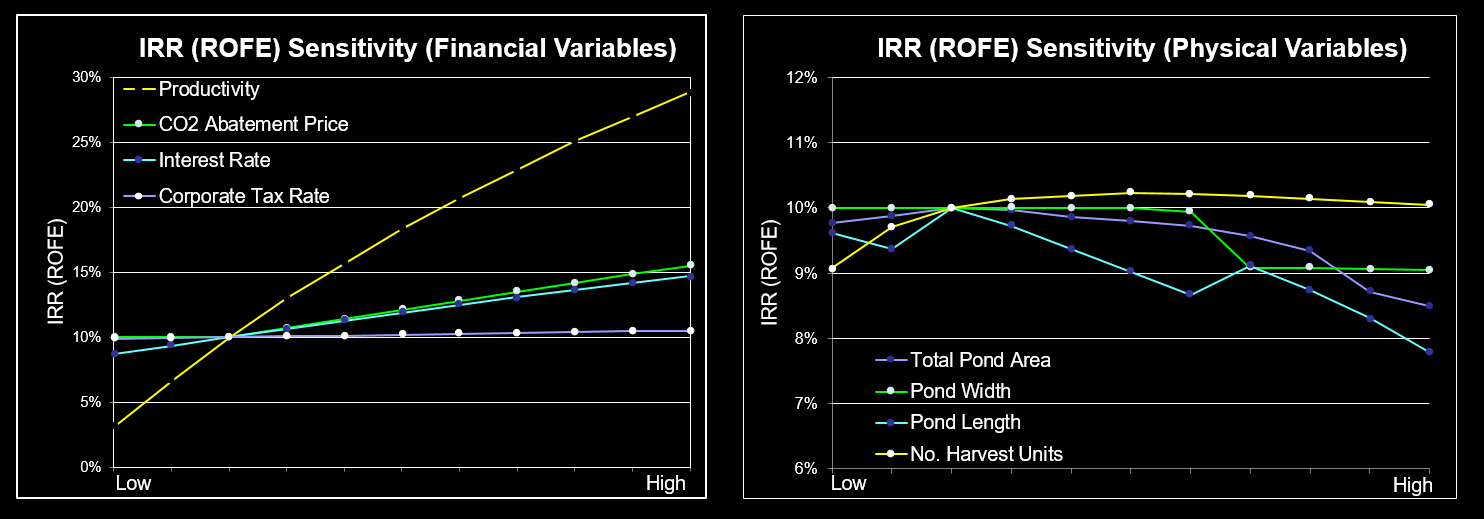


**Figure S25.** Sample Sensitivity Graphs. Output variable sensitivity graphs were produced to test responses (financial, energy and GHG emissions) to an extended array of input variables. This figure is a sample only of the full array of inputs and outputs tested. All the curves exhibit expected responses. Pond Length and Pond Width exhibit stepped responses as the model adjusts the pond array configuration to fit within the specified total pond area.

### **External validation**

With the testing of modules and the entire TELCA workflow complete (Section10, above), another level of testing was necessary; *benchmarking* TELCA against the best ‘*international standard’* models. A series of previous published papers documenting various models of microalgae fuel production processes have been published but reported a wide range of price results for the production of renewable diesel fuel (Figure S26).

The major discrepancy in these reported prices is due to the wide range of assumptions made regarding the critical input parameters. To validate the TELCA model it was important to mimic, as far as possible, inputs for the best of these existing models and investigate any output discrepancies. The *NREL/PNNL/ANL* model was selected for this purpose. This model^49^ was first published in 2011 with a follow up papers in 2012^29^ and in 2014 ^28^ which included HTL as the primary fuel extraction process, but with prices continuing to be referenced back to the 2011 base. In TELCA several key variables were adjusted to match the inputs nominated in the supplementary material provided with this published model. These included:

***405 ha facility built in the US Gulf Coast region***

***Target IRR of 10%***

***Annual average algae productivity of 14.6 g.m^-2^.d^-1^***

***The use of freshwater as the basis of production***

***Use of chemical flocculation and DAF for dewatering***

***Farm size and pond sizes***

***Annual operating days of 330***

***Corporate tax rate of 35%***

***Correcting the prices back to a 2011 base***

The MDSP price derived for the *TELCA model* under these variable settings was:

USD$11.10 gal^-1^ or USD$2.93 L^-1^

This MDSP price derived for the *NREL/PNNL/ANL* model was:

USD$11.00 gal^-1^ or USD$2.91 L^-1^

**TELCA external benchmarking:** The difference of the calculated Mean Diesel Selling Price of *less than 1%* (TELCA: $11.10 L^-1^ vs NREL/PNNL/ANL: $11.00) *provides a robust confirmation of TELCA’s performance*.

Due to the lack of sufficient data provided in the NREL/PNNL/ANL model, it was recognised however, that some of the process configuration (use of AD *in lieu* of CHG and the use of *PEM* versus *steam reformation* derived hydrogen) did not match exactly the configuration used by NREL/PNNL/ANL. The expected effects of these discrepancies is however minor and detailed below:

**Use of Anaerobic digesters versus use of CHG**. The capital cost for AD is approximately USD$3.1M instead of the cost nominated by NREL/PNNL/ANL of USD$4.1M for the CHG plant. This difference translates to a decrease in the MDSP of USD$0.007 L^-1^ (0.06%). Catalyst demand for the CHG plant translates to an additional USD$0.032 L^-1^ (0.3%).^.^ Clear information on the other operating costs of the CHG facility were not available and so it was assumed that these costs would be similar to the AD plant. *The net effect is that the NREL/PNNL/ANL system should be USD$0.04 L^-1^ (1.3%) higher in MDSP*.

**Use of PEM hydrogen generation versus use of Steam Reforming**. The TELCA PEM plant including hydrogen storage has a capital cost of USD$13.8M and an annual power supply cost of USD$0.4 M. The NREL/PNNL/ANL steam reformer has a capital cost of USD$2.9M. The cost of natural gas supply was not provided in the supplementary information. Based on a price of USD$4.00 GJ^-1^ (average Henry Hub price for 2011, www.macrotrends.net/2478/natural-gas-prices-historical-chart) and the stated hydrogen production efficiency of 90%, the annual natural gas cost would be approximately USD$1.3M. As no information was available for the other operating costs, it was reasonable to assume that these costs would be similar for both alternative methods of hydrogen production. *The net effect is that the NREL/PNNL/ANL system should be USD$0.04 L^-1^ (1.4%) lower in MDSP.*

The net effect of the above two process changes is that the NREL/PNNL/ANL plant should deliver a MDSP equivalent to that of the TELCA model. The actual difference in results (USD$2.93 L^-1^ compared to USD$2.91 L^-1^ represents a difference of 0.7% with the TELCA model reflecting a slightly higher and so more conservative price.

The comparison of the TELCA modelled MDSP to a range of other models is shown on Figure S26. Note, however, that the detailed comparison analysis carried out for the NREL/PNNL/ANL model was not performed on the other results referenced in the figure. The validation of TELCA has therefore been very rigorous compared to most published studies.


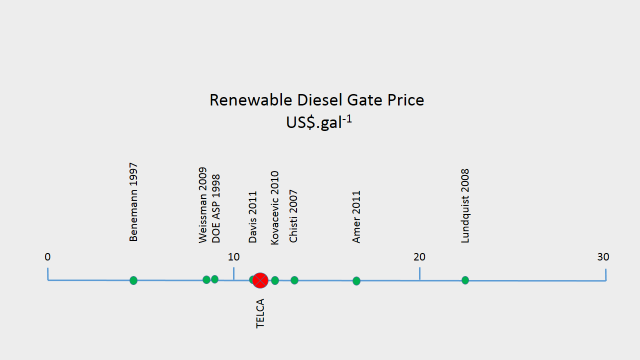


**Figure S26.** Range of Reported Renewable Diesel Prices. Range of renewable diesel refinery gate prices reported by various published models.

**ERoEI and GHG emissions**

The comparative analysis of the *energy return* and the *GHG emissions* was more complex than the *financial* comparison. NREL/PNNL/ANL reported a fossil energy consumption rate of 0.68 MJ.MJ^-1^ in the original 2011 study and 0.44 MJ.MJ^-1^ in the 2014 analysis including the HTL process for extraction. If all external energy supply was assumed to be derived from fossil based sources then these rates would translate to ERoEI values of 1.47 ^29^ and 2.72 ^28^ respectively. The TELCA model in the comparative format used for validation returned an ERoEI figure of 1.35. This fossil energy input rate is dependent on the fossil energy component of external power supplies. The TELCA model was based on an assumption that external power supply was derived partially from fossil fuel sources with an emissions rate of 0.35 kgCO_2_eq.kWhr^-1^ i.e. approximately half the current prevailing rate. It was unclear from the NREL/PNNL/ANL data what assumptions were made in this respect. It was also unclear as to why the energy consumption altered so dramatically between the two studies by NREL/PNNL/ANL.

There was a similar discrepancy between the GHG comparisons in the NREL/PNNL/ANL study. In the 2011 NREL/PNNL/ANL study a GHG emissions rate of 67 gCO_2_eq.MJ^-1^ was reported, while in the 2014 study incorporating HTL a rate of 38 gCO_2_eq.MJ^-1^ was used. The difference is difficult to explain and was not covered in the supplementary data. The TELCA model returned a GHG emission rate of 66 gCO_2_eq.MJ^-1^. While this rate closely matches the initial 2011 NREL/PNNL/ANL study, it is significantly different from the rate reported for the HTL process version.

The author (John Roles) therefore contacted one of the NREL/PNNL/ANL authors to establish the basis for the discrepancies and the following items were identified. Importantly, both models used the same methodology in respect to the treatment of fugitive emissions from pond injection of CO_2_. One of the reasons for the change in emissions rates between the two papers was the method of hydrogen production. In the first NREL/PNNL/ANL paper, *hydrogen was considered a purchased item* and the *associated CO_2_ produced* in generating the hydrogen using steam reforming would have resulted in significant CO_2_ emissions. These emissions were eliminated in the subsequent process, incorporating *onsite production of hydrogen* where CO_2_ is captured for injection into the algae ponds.

**Appendix 1. TELCA Base case variable settings and ranges for sensitivity analysis.**

*User defined variables* are shaded orange, *calculated variables* are shaded blue, *descriptions* are shaded white. Where *ranges have been fixed lower (Low) and upper (High) limits* are stated in the table.

| **Parameter** | **Units** | **Base Case** | **Range** | | **Description/Reference** |
| --- | --- | --- | --- | --- | --- |
|  |  | **Set Point** | **Low** | **High** |  |
| **Financial** |  |  |  |  |  |
| Currency Conversion |  |  |  |  |  |
| USD = AUD * | USD | 1.33 |  |  | Input Variable |
| USD = CNY * | USD | 6.67 |  |  | Input Variable |
|  |  |  |  |  |  |
| Annual Production Duration | h | 8,640 |  |  | Based on continuous year round operation with the only down time being a total of 126 hours for pond repairs/maintenance and unplanned outages. |
| Financial Analysis |  |  |  |  | Discounted cash-flow |
| Project Life (including construction and start up) | Years | 30 |  |  | Based on average expected equipment life. |
| Loan Repayment Period | Years | 15 |  |  |  |
| Target IRR |  | 10% |  |  |  |
|  |  |  |  |  |  |
| Interest Rate |  | 8% | 0% | 10% | Input Variable |
| Equity Ratio |  | 40% |  |  | Based on typical industry standard given this is an emerging technology in a competitive field |
| Depreciation Rate |  | 5% |  |  | Input Variable |
| Tax Rate |  | 21% | 13% | 23% | Input Variable |
|  |  |  |  |  |  |
| **Development Costs** |  |  |  |  |  |
| Land purchase price | A$.ha-1 | 10,000 | 0 | 12,500 | Land acquisition price including transfer costs |
| Development Approval |  | 0.20% |  |  | Percent of CapEx value |
| Environmental Approval |  | 0.10% |  |  | Percent of CapEx value |
| Community Consultation |  | 0.10% |  |  | Percent of CapEx value |
| Loan Establishment |  | 1.50% |  |  | Percent of Loan value |
| Loan maintenance fee (Bankers Engineer) |  | 0.30% |  |  | Percent of Loan value |
| Working Capital (Alternative 1) |  | 5% |  |  | Percent of CapEx over plant life. |
| Working Capital (Alternative 2) |  |  |  |  | Alternative based on a significant portion of the asset being low risk civil works and a staged start-up regime. |
|  |  | 5% |  |  | Percentage of CapEx during construction period |
|  |  | 15% |  |  | Percentage of OpEx after construction period completed |
|  |  |  |  |  |  |
| CapEx Expenditure Year 1 |  | 20% |  |  | Percent of CapEx costs |
| CapEx Expenditure Year 2 |  | 50% |  |  | Percent of CapEx costs |
| CapEx Expenditure Year 3 |  | 30% |  |  | Percent of CapEx costs |
| OpEx Setup Costs Year 2 |  | 50% |  |  | Percent of standard yearly OpEx costs |
| OpEx Setup Costs Year 3 |  | 100% |  |  | Percent of standard yearly OpEx costs |
|  |  |  |  |  |  |
|  |  |  |  |  |  |
| **Design and Project Management Costs** |  |  |  |  |  |
| Civil Design | h | 200 |  |  | Design hours per A$M of respective CapEx |
| Structural Design | h | 250 |  |  | Design hours per A$M of respective CapEx |
| Piping Design | h | 250 |  |  | Design hours per A$M of respective CapEx |
| Mechanical Design | h | 350 |  |  | Design hours per A$M of respective CapEx |
| Electrical Design | h | 800 |  |  | Design hours per A$M of respective CapEx |
| Controls Design | h | 1,000 |  |  | Design hours per A$M of respective CapEx |
| 3rd Party Design Approvals |  | 10% |  |  | Percent of total design hours |
| Project Management | h | 1,000 |  |  | Base hours plus |
|  | h | 80 |  |  | Hours per A$M of project CapEx |
| Project Support | h | 500 |  |  | Base hours plus |
|  | h | 100 |  |  | Hours per A$M of project CapEx |
| Procurement | h | 200 |  |  | Base hours plus |
|  | h | 150 |  |  | Hours per A$M of project CapEx |
|  |  |  |  |  |  |
| Design hourly rate | A$.h^-1^ | 150 |  |  | Range representing the difference between China and US costs |
| 3rd party design approval rate factor |  | 1.25 |  |  | Cost factor applied to base design hourly rate |
| Project Management Rate Factor |  | 1.3 |  |  | Cost factor applied to base design hourly rate |
| Project Support Rate Factor |  | 0.4 |  |  | Cost factor applied to base design hourly rate |
| Project Procurement Rate Factor |  | 1 |  |  | Cost factor applied to base design hourly rate |
| Design and Project Management Office Costs | A$.h^-1^ | 20 |  |  | Rate applied to total design and project management hours |
| Design and Project Management Travel and Accomodation | A$.h^-1^ | 5 |  |  | Rate applied to total design and project management hours |
|  |  |  |  |  |  |
| **Open High Rate Ponds** |  |  |  |  |  |
| Production Concentration | g.L^-1^ | 0.163 | 0.1 | 0.415 | Production concentration in g.L^-1^ |
|  |  |  |  |  |  |
| Latitude |  | 28 | 20 | 57.5 | Latitude in degrees |
| Location Impact Factor |  | 0.95 |  |  | Allowance factor representing growth loss due to weather at location |
| Corrected Production Rate | g.m^-2^.d^-1^ | 14.62 | 10.8 | 30 | Production rate incorporating weather, location, depth and concentration inputs |
| Biomass Carbon to green crude conversion efficiency |  | 55.0% |  |  |  |
| CO_2_ Fixing Efficiency |  | 80% |  |  | Rate of injected CO_2_ retained within the culture and available for biomass take-up |
| CO_2_ Supply concentration |  | 11.0% |  |  | Concentration of CO_2_ in supply gas to the plant from conjoined CO_2_ emitter |
| Pond Culture CO_2_ Conc. |  | 1% |  |  | Culture average concentration |
| CO_2_ Pricing | US$.T^-1^ | 0 | 0$ | $120 | Forecast carbon emission prices ^50^ |
| Culture Salt Concentration |  | 5% |  |  | Nominal concentration under standard operating conditions |
| Makeup Water Salt Conc. |  | 2.995% |  |  | Average salinity for natural sea water |
|  |  |  |  |  |  |
| Total Pond Area | ha | 570 | 470 | 970 | Nominal pond wet surface area not including buffer/overflow ponds |
| Nominal Pond Length | m | 1,050 | 650 | 1150 | Pond length to centreline of bund |
| Nominal Pond Width | m | 48 | 40 | 50 | Pond length to centreline of bund |
| Nominal Pond Depth | m | 0.21 | 0.20 | 0.25 | Average pond depth over working surface during operation |
| Bund Freeboard | m | 0.2 |  |  | Bund height above average pond water level |
| Bund Width | m | 0.5 |  |  | Width at top of bund |
| Average Excavation Depth | m | 0.10 |  |  | Average excavation volume for ponds based on cut and fill over each pond area. |
|  |  |  |  |  |  |
| Liner Type |  |  |  |  | Polyethylene |
| Liner Thickness | mm | 1 |  |  | Selected to match PNNL nominated liner thickness |
| Liner Substrate depth | m | 0.05 |  |  | Refined sand fill to support liner |
| Friction Coefficient |  | 0.015 |  |  | Friction coefficient for PE lined channel. |
| Mixer Type |  |  |  |  | Paddlewheel |
| Pond Flow Speed | m.s^-1^ | 0.25 |  |  | Selected value to optimise between mixing required for maximum growth and power cost |
| Mixing Power Consumption |  |  |  |  | Calculated using Manning equations |
| Mixer mechanical efficiency |  | 80% |  |  |  |
| Max. Mixer Head Loss (m) | m | 0.05 |  |  | The number of mixers are calculated based on maximum head loss across each mixer |
| Mixing energy | kWhr/ha/d | 16.1 |  |  |  |
|  |  |  |  |  |  |
| Overflow Pond Capacity |  | 10% |  |  | Percentage of total pond volume set aside for recycling of pond water during cleaning operations and allowance for containment during rain events. |
| Overflow Pond Depth | m | 5 |  |  | Cost optimised pond construction depth under normal site and soil conditions. |
|  |  |  |  |  |  |
| **Concrete works** |  |  |  |  |  |
| Paddle Wheels per pond | m^3^ | 12.48 |  |  | Scaled based on equipment size and number |
| Emergency Spillway per pond | m^3^ | 42 |  |  | Scaled based on pond volume |
| Minor Footings per pond | m^3^ | 2 |  |  | Scaled based on equipment size and number |
|  |  |  |  |  |  |
| **Electro-flocculation** |  |  |  |  |  |
| Number Of Units |  | 16 |  |  | Number of flocculation units dispersed among ponds |
| Separator Cut Ratio |  | 5% |  |  | Product stream proportion of inlet stream v/v |
| Separation Efficiency |  | 90% |  |  | Proportion of input algal biomass directed into the product stream |
| Flocculation residence time | s | 30 |  |  | Used for setting flocculation tank volume |
| Settling residence time | h | 1 |  |  | Used for setting settling tank volume |
| EF Tank | m^3^ | 4.0 |  |  | PE Tank volume scaled based on anode area and spacing requirements |
| Settling Tank | m^3^ | 405 |  |  | Concrete tank scaled based on residence time |
| Harvest Power Consumption | kWh.m^-3^ | 0.09 |  |  | Harvesting Marine Microalgae Lee A. 2012 |
| Electrode Consumption Rate | kg.m^-3^ | 0.0086 |  |  | Harvesting Marine Microalgae Lee A. 2012 |
|  |  |  |  |  |  |
| **Centrifuge** |  |  |  |  |  |
| Product Moisture Content |  | 80% |  |  | Water content w/w |
| Separation Efficiency |  | 95% |  |  | Proportion of input algal biomass directed into the product stream |
| Power Consumption Rate | kWh.m^-3^ | 0.386 |  |  | Power consumption based on input volume |
| Building Footprint | m^2^ | 90 |  |  | Scaled based on number and size of machines with additional foundation allowance for vibration suppression. |
|  |  |  |  |  |  |
| **HTL** |  |  |  |  |  |
| Reactor Type |  |  |  |  | Plugged flow reactor with tube in tube heat exchange and pressure energy recovery |
| Reactor Temperature | C^o^ | 351 |  |  |  |
| Reactor Pressure | kPaA | 20,947 |  |  |  |
| Reactor Residence Time | h | 0.25 |  |  |  |
| Mass input to Oil Phase |  | 59% |  |  | Yield based on w/w of algae ash free dry weight |
| Mass input to Aqueous Phase |  | 34% |  |  | Yield based on w/w of algae ash free dry weight |
| Mass input to Gas phase |  | 4% |  |  | Yield based on w/w of algae ash free dry weight |
| Mass input to Solids phase |  | 2% |  |  | Yield based on w/w of algae ash free dry weight |
| HEX K Rate | W.m^-2^.K^-1^ | 1,000 |  |  | Reactor heat exchanger heat transfer rate |
| LP Pump mechanical efficiency |  | 60% |  |  | Based on high viscosity fluid |
| HP Pump mechanical efficiency |  | 65% |  |  | Based on high viscosity fluid |
| Pressure Energy Recovery Rate |  | 50% |  |  | Based on high viscosity fluid |
| Reactor Heater Temperature | C^o^ | 373 |  |  | Heater fluid working temperature |
| Heater Flue Gas TTD | C^o^ | 60 |  |  | Terminal temperature differential for flue heater flue gas |
| Heater K Rate | W.m^-2^.K^-1^ | 60 |  |  | Gas firing heat transfer rate |
| Building Footprint | m^2^ | 298 |  |  | Scaled based on number and size of equipment |
| Separator Residence time | h | 0.5 |  |  |  |
|  |  |  |  |  |  |
| **Volatile Recovery** |  |  |  |  |  |
| Type |  |  |  |  | Distillation column with reflux and heat recovery |
| Residence Time | h | 0.1 |  |  |  |
| Heat Supply |  |  |  |  | Waste heat from HTL |
| Heater K Rate | W.m^-2^.K^-1^ | 60 |  |  | Gas firing heat transfer rate |
| Heater Flue Gas TTD | C^o^ | 30 |  |  | Terminal temperature differential for heater |
| Recovery HEX K Rate | W.m^-2^.K^-1^ | 1,000 |  |  | Recovery heat exchanger heat transfer rate |
| Recovery HEX TTD | C^o^ | 10 |  |  | Terminal temperature differential for Recovery heat exchanger |
| Product Cooler K Rate | W.m^-2^.K^-1^ | 60 |  |  | Product Cooler heat transfer rate |
| Product Cooler Average Temp Diff (C^o^) | C^o^ | 60 |  |  | Average temperature differential for product cooler |
| Product Forwarding Pump Efficiency |  | 70% |  |  | Combined motor pump efficiency |
| Aqueous Forwarding Pump Efficiency |  | 70% |  |  | Combined motor pump efficiency |
| Non Condensibles pump Efficiency |  | 70% |  |  | Combined motor pump efficiency |
| Building Footprint | m^2^ | 46 |  |  | Scaled based on number and size of equipment |
| Volatile Recovery Rate |  | 50% | 35% | 60% |  |
|  |  |  |  |  |  |
| **Refining** |  |  |  |  |  |
| Hydrotreater H_2_ Consumption |  | 4% |  |  |  |
| Hydrotreater Hydrocarbon product |  | 78% |  |  |  |
| Hydrotreater water product |  | 11% |  |  |  |
| Hydrotreater CO_2_ product |  | 11% |  |  |  |
| Hydrotreater Temperature | C^o^ | 400 |  |  |  |
| Hydrotreater Preasure | kPa | 13,600 |  |  |  |
| Liquid space velocity | h^-1^ | 0.2 |  |  |  |
|  |  |  |  |  |  |
| LP Feed Pump Efficiency |  | 60% |  |  | Combined motor pump efficiency |
| HP Feed Pump Efficiency |  | 65% |  |  | Combined motor pump efficiency |
| Recirc. Pump Efficiency |  | 70% |  |  |  |
|  |  |  |  |  |  |
| Distillation Column Temperature | C^o^ | 370 |  |  |  |
| Distillation Column Pressure | kPa | 7,000 |  |  |  |
| Liquid space velocity | h^-1^ | 0.2 |  |  |  |
| Recirc. Pump Efficiency |  | 70% |  |  | Combined motor pump efficiency |
|  |  |  |  |  |  |
| HT Product requiring cracking |  | 50% |  |  |  |
| Hydrocracker H2 Consumption |  | 2% |  |  |  |
| Hydrocracker Fuel product |  | 86% |  |  |  |
| Hydrocracker water product |  | 6% |  |  |  |
| Hydrocracker CO_2_ product |  | 8% |  |  |  |
| Hydrocracker Temperature | C^o^ | 370 |  |  |  |
| Hydrocracker Pressure | kPa | 7000 |  |  |  |
| Liquid space velocity | h^-1^ | 0.2 |  |  |  |
| Recirculation Pump Efficiency |  | 70% |  |  | Combined motor pump efficiency |
|  |  |  |  |  |  |
| Product HEX K Rate | W.m^-2^.K^-1^ | 1,000 |  |  | Recovery heat exchanger (HEX) heat transfer rate |
| Product HEX Average Temp Differential | C^o^ | 30 |  |  | Average temperature differential for heat exchanger |
| Product HEX K Rate | W.m^-2^.K^-1^ | 1,000 |  |  | Recovery heat exchanger heat transfer rate |
| Product HEX Average Temp Diff. | C^o^ | 135 |  |  | Average temperature differential for heat exchanger |
|  |  |  |  |  |  |
| H_2_ Production PEM Unit operating hours per day |  | 8 |  |  | Capacity oversize contingency |
| Power Consumption Rate | kWh.kg^-1^ | 54 | 46 | 56 | Power input for hydrogen production. DOE Hydrogen Fuel Cell Program Record 2015 |
| H_2_ Storage | h | 18 |  |  | Storage size determined by peak usage rate |
| Storage Pressure | kPa | 8,000 |  |  |  |
| Building Footprint | m^2^ | 118 |  |  | Scaled based on number and size of equipment |
|  |  |  |  |  |  |
| **Digester** |  |  |  |  | Lined steel tank |
| Residence time | d | 30 |  |  |  |
| Liquid capacity |  | 90% |  |  | Proportion of tank size for liquid residence |
| Mixing demand | kWhr.m^-3^.d^-1^ | 0.017 |  |  | Tank mixer size basis. Lemmer 2013 |
| Mixer Selection Size |  | 150% |  |  | Tank mixer size basis |
|  |  |  |  |  |  |
| CH_4_ Production Rate of VS | kg.kg^-1^ | 0.357 |  |  | De Mes ^51^ |
| CO_2_ Production Rate of VS | kg.kg^-1^ | 0.148 |  |  | De Mes ^51^ |
| Soluble Carbon Products of VS | kg.kg^-1^ | 0.416 |  |  |  |
| Sludge production rate of VS | kg.kg^-1^ | 0.104 |  |  |  |
|  |  |  |  |  |  |
| **CHP Plant** |  |  |  |  |  |
| Type |  |  |  |  | Gas fired reciprocating engine |
| Engine Heat Rate | kJ.kWh^-1^ | 10,000 |  |  | Combined net power heat rate. |
| Building Footprint | m^2^ | 42 |  |  | Scaled based on number and size of equipment |
|  |  |  |  |  |  |
| **Piping** |  |  |  |  |  |
| CO2 Piping type |  |  |  |  | GRP |
| Low Temperature/Pressure Applications |  |  |  |  | PE |
| Other |  |  |  |  | Carbon or Stainless Steel as applicable |
| Maximum Gas velocity | m.s^-1^ | 20 |  |  |  |
| Maximum water velocity | m.s^-1^ | 2 |  |  |  |
| Flow Capacity Factor |  | 110% |  |  |  |
| Plant Layout |  |  |  |  | Overall plant layout consists of an array of ponds oriented with the longitudinal axis in the North-South direction. Ponds are arranged in rows with the rows extending equally east and west of a central North-South spine. The main processing facility is located at the centre of the plant. The pond related piping systems consist of stems running the width of the plant from East to West between pairs of pond rows. |
| Pond Configuration |  |  |  |  | Automatic configuration to optimise layout to a approximate a square plot of minimum dimensions. All ponds configured North- South |
| Plot Length East - West | m | 2,199 |  |  | Calculated from inputs |
| Plot Length North - South | m | 2,134 |  |  | Calculated from inputs |
| Plot Area | ha | 469 |  |  | Calculated from inputs |
| CO_2_ supply point | m | 50 |  |  | Metres outside plant boundary |
| Seawater Supply point | m | 50 |  |  | Metres outside plant boundary |
| Freshwater supply point | m | - |  |  | Metres outside plant boundary |
| Pump efficiency |  | 70% |  |  | General efficiency for pumping low solids water unless indicated elsewhere. |
| Pump Motor Efficiency |  | 90% |  |  |  |
|  |  |  |  |  |  |
| **Power Systems** |  |  |  |  |  |
| System |  |  |  |  | 11kV supply to central processing unit and distributed to primary dewatering nodes. 415V distribution within CPU and to pond pumps from nodes. Separate 11kV supply locally to CO_2_ blowers. |
| Support systems |  |  |  |  | Direct buried cable for all field runs. |
| 11 kVSupply |  |  |  |  | Plant boundary. |
| Normal Demand Load | kW | 4,888 |  |  | Scaled by equipment demand excluding CO_2_ blower and H_2_ production |
| Peak Load | kW | 9,211 |  |  | Scaled by equipment demand excluding CO_2_ blower and H_2_ production |
| CO_2_ Blower Normal Demand | kW | 1,226 |  |  | Scaled by equipment demand. |
| CO_2_ Blower Peak Demand | kW | 1,571 |  |  | Scaled by equipment demand. |
| H_2_ Production Demand | kW | 17,880 |  |  | Scaled by equipment demand. |
| H_2_ Peak Production Demand | kW | 17,970 |  |  | Scaled by equipment demand. |
| Power supply from Renewable sources |  | 0% | 0% | 100% |  |
| Non- Renewable Power supply price | A$/kWh | 0.116 |  |  |  |
| Non- Renewable Power GHG emissions | TCO_2_eMWh^-1^ | 0.36 |  |  |  |
| Renewable Power supply price | A$/kWh | 0.04 |  |  |  |
| Renewable Power supply embodied GHG emissions | TCO_2_eMWh^-1^ capacity | 0.05 |  |  |  |
| **Control Systems** |  |  |  |  |  |
| System |  |  |  |  | Localised PLC linked through ethernet to central control room |
| I/O |  | 1,023 |  |  | Scaled by equipment numbers |
|  |  |  |  |  |  |
| **Water Supply System** |  |  |  |  |  |
| Average Flow Rate | kL.d^-1^ | 8,158 |  |  | Calculated from site environmental conditions |
| Peak Flow Rate | kL.d^-1^ | 29,973 |  |  | Calculated from site environmental conditions |
| Filter screen size | Micron | 100 |  |  |  |
| Feed Tank Residence time | h | 2 |  |  |  |
|  |  |  |  |  |  |
| **Gas Supply System** |  |  |  |  |  |
| CO_2_ Supply Temperature | C^o^ | 120 |  |  | Assumed gas supply taken from outlet conditions after CCGT power station. |
| CO_2_ Transmission Temperature | C^o^ | 60 |  |  |  |
| Cooling Equipment |  |  |  |  | Gas quenching using spray evaporator |
| Water Volume | L.hr^-1^ | 3,074 |  |  | Fresh water scaled to gas flow requirements |
| Water Removal |  |  |  |  | Knockout Drum with mist eliminator |
| Knockout Drum Retention Time | s | 2 |  |  |  |
|  |  |  |  |  |  |
| **Nutrient Supply** |  |  |  |  |  |
| Primary Nutrient |  | CH_4_N_2_O |  |  | Primary nutrient for the supply of Nitrogen. Other nutrients quantified to match. |
| Storage Replenish Time | d | 7 |  |  | Bulk delivery of nitrogen and phosphate. Other nutrients by bag. |
| Storage Buffer |  | 50.0% |  |  |  |
| Primary Nutrient Supply Price | AUD$.T^-1^ | 600 | 400 | 650 |  |
|  |  |  |  |  |  |
| **Construction Labour** |  |  |  |  |  |
| **Location Alternative 1** |  | **USA Rural** |  |  |  |
| Productivity |  | 90.9% |  |  | Productivity factor applied to base estimate hours |
| Base Rate | AUD$.h^-1^ | 34.67 |  |  | Base labour rate before OT and loadings |
| Base Hours | h.w^-1^ | 40.00 |  |  | Weekly hours at base rate |
| OT Hours | h.w^-1^ | 10.00 |  |  | Weekly overtime hours |
| OT Loading |  | 50% |  |  | Overtime loading to base rate |
| Crew Loading |  | 12% |  |  | Average loading onto base rate for full construction crew (gang rate) |
| Statutory Oncosts |  | 23% |  |  | On costs including health cover, payroll tax, leave costs and superannuation. |
| Acc/Travel, Consumables and Equipment | AUD$.h^-1^ | 40.40 |  |  | Site equipment and consumable costs |
| Contractors Margin |  | 15% |  |  | Profit and risk margin by contractor. |
|  |  |  |  |  |  |
| **Location Alternative 2** |  | **China Rural** |  |  |  |
| Productivity |  | 66.7% |  |  | Productivity factor applied to base estimate hours |
| Base Rate | AUD$.h^-1^ | 4.81 |  |  | Base labour rate before OT and loadings |
| Base Hours | h.w^-1^ | 40.00 |  |  | Weekly hours at base rate |
| OT Hours | h.w^-1^ | 10.00 |  |  | Weekly overtime hours |
| OT Loading |  | 0% |  |  | Overtime loading to base rate |
| Crew Loading |  | 12% |  |  | Average loading onto base rate for full construction crew (gang rate) |
| Statutory Oncosts |  | 69% |  |  | On costs including health cover, payroll tax, leave costs and superannuation. |
| Acc/Travel, Consumables and Equipment | AUD$.h^-1^ | 30.40 |  |  | Site equipment and consumable costs |
| Contractors Margin |  | 20% |  |  | Profit and risk margin by contractor. |
|  |  |  |  |  |  |
| **Location Alternative 3** |  | **Australia Rural** |  |  |  |
| Productivity |  | 75.2% |  |  | Productivity factor applied to base estimate hours |
| Base Rate | AUD$.h^-1^ | 17.65 |  |  | Base labour rate before OT and loadings |
| Base Hours | h.w^-1^ | 38.00 |  |  | Weekly hours at base rate |
| OT Hours | h.w^-1^ | 12.00 |  |  | Weekly overtime hours |
| OT Loading |  | 58% |  |  | Overtime loading to base rate |
| Crew Loading |  | 12% |  |  | Average loading onto base rate for full construction crew (gang rate) |
| Statutory Oncosts |  | 37% |  |  | On costs including health cover, payroll tax, leave costs and superannuation. |
| Acc/Travel, Consumables and Equipment | AUD$.h^-1^ | 49.40 |  |  | Site equipment and consumable costs |
| Contractors Margin |  | 15% |  |  | Profit and risk margin by contractor. |
|  |  |  |  |  |  |
| **OPERATIONS** |  |  |  |  |  |
|  |  |  |  |  |  |
| **Open High Rate Ponds** |  |  |  |  |  |
| Liner Repairs | h.y^-1^ | 27 |  |  | Maintenance and repairs on a per pond basis |
| Mixer Repairs | h.y^-1^ | 4 |  |  | Maintenance and repairs on a per mixer basis |
| CO_2_ Diffuser Repairs | h.y^-1^ | 33 |  |  | Maintenance and repairs on a per pond basis |
| Online pond cleaning | h.y^-1^ | 134 |  |  | Maintenance and repairs on a per pond basis |
| Full pond cleaning | h.y^-1^ | 100 |  |  | Maintenance and repairs on a per pond basis |
| General Maintenance | h.y^-1^ | 36 |  |  | Maintenance and repairs on a per pond basis |
|  |  |  |  |  |  |
| **Electroflocculation** |  |  |  |  |  |
| Anode Change per unit | h.y^-1^ | 126 |  |  | Service hours per unit. Scaled based on anode mass |
| General maintenance per unit | h.y^-1^ | 380 |  |  | Service hours per unit. Scaled based on equipment size |
|  |  |  |  |  |  |
| **Centrifuge** |  |  |  |  |  |
| Inspections | h.y^-1^ | 208 |  |  | Inspection hours per machine |
| Service Hours | h.y^-1^ | 133 |  |  | Scheduled service hours per machine. Scaled by machine size. |
| Unscheduled repairs | h.y^-1^ | 16 |  |  | Repair hours per machine |
| Replacement wear liners | AUD$.y^-1^ | 41,834 |  |  | Supply costs per machine. Scaled based on equipment CapEx |
| Replacement parts | AUD$.y^-1^ | 52,293 |  |  | Supply costs per machine. Scaled based on equipment CapEx |
|  |  |  |  |  |  |
| **HTL** |  |  |  |  |  |
| Inspections | h.y^-1^ | 728 |  |  | Inspection hours including third party inspections |
| Service Hours | h.y^-1^ | 114 |  |  | Scheduled service hours. Scaled by machine size. |
| Unscheduled repairs | h.y^-1^ | 100 |  |  | Repair hours. Scaled by machine size |
|  |  |  |  |  |  |
| **Volatile Recovery** |  |  |  |  |  |
| Inspections | h.y^-1^ | 368 |  |  | Inspection hours including third party inspections |
| Service Hours | h.y^-1^ | 48 |  |  | Scheduled service hours. Scaled by machine size. |
| Unscheduled repairs | h.y^-1^ | 40 |  |  | Repair hours. Scaled by machine size |
|  |  |  |  |  |  |
| **Refining** |  |  |  |  |  |
| Inspections | h.y^-1^ | 1050 |  |  | Inspection hours including third party inspections |
| Service Hours | h.y^-1^ | 605 |  |  | Scheduled service hours. Scaled by equipment size. |
| Maintenance Materials | AUD$.y^-1^ | 607,492 |  |  | Scaled based on equipment CapEx including catalyst replacement |
| Unscheduled repairs | h.y^-1^ | 260 |  |  | Repair hours. Scaled by equipment size |
|  |  |  |  |  |  |
| **Digester** |  |  |  |  |  |
| Inspections | h.y^-1^ | 104 |  |  | Inspection hours including third party inspections |
| Service Hours | h.y^-1^ | 144 |  |  | Scheduled service hours. Scaled by equipment size. |
| Maintenance Materials | AUD$.y^-1^ | 22,216 |  |  | Scaled based on equipment and liner size |
| Unscheduled repairs | h.y^-1^ | 208 |  |  | Repair hours. Scaled by equipment size |
|  |  |  |  |  |  |
| **CHP Plant** |  |  |  |  |  |
| Inspections | h.y^-1^ | 720 |  |  | Inspection hours including third party inspections |
| Service Hours | h.y^-1^ | 124 |  |  | Scheduled service hours. Scaled by equipment size. |
| Maintenance Materials | AUD$.y^-1^ | 34,000 |  |  | Scaled based on equipment size |
| Unscheduled repairs | h.y^-1^ | 52 |  |  | Repair hours. Scaled by equipment size |
|  |  |  |  |  |  |
| **Piping** |  |  |  |  |  |
| Inspections | h.y^-1^ | 921 |  |  | Inspection hours including third party inspections |
| Service Hours | h.y^-1^ | 3,450 |  |  | Scheduled service hours. Scaled by equipment size. |
| Maintenance Materials | AUD$.y^-1^ | 136,143 |  |  | Scaled based on equipment size |
|  |  |  |  |  |  |
| **Power Systems** |  |  |  |  |  |
| Inspections | h.y^-1^ | 830 |  |  | Inspection hours including third party inspections |
| Service Hours | h.y^-1^ | 240 |  |  | Scheduled service hours. Scaled by equipment size. |
| Maintenance Materials | AUD$.y^-1^ | 126,497 |  |  | Scaled based on equipment size |
| Unscheduled repairs | h.y^-1^ | 1597 |  |  | Repair hours. Scaled by equipment size |
|  |  |  |  |  |  |
| **Controls Systems** |  |  |  |  |  |
| Inspections | h.y^-1^ | 513 |  |  | Inspection hours including third party inspections |
| Service Hours | h.y^-1^ | 769 |  |  | Scheduled service hours. Scaled by equipment size. |
| Maintenance Materials | AUD$.y^-1^ | 53,973 |  |  | Scaled based on equipment size |
| Unscheduled repairs | h.y^-1^ | 1281 |  |  | Repair hours. Scaled by equipment size |
|  |  |  |  |  |  |
| **Water Supply System** |  |  |  |  |  |
| Inspections | h.y^-1^ | 720 |  |  | Inspection hours including third party inspections |
| Service Hours | h.y^-1^ | 104 |  |  | Scheduled service hours. Scaled by equipment size. |
| Unscheduled repairs | h.y^-1^ | 130 |  |  | Repair hours. Scaled by equipment size |
|  |  |  |  |  |  |
|  |  |  |  |  |  |
|  |  |  |  |  |  |
| **Gas Supply System** |  |  |  |  |  |
| Inspections | h.y^-1^ | 360 |  |  | Inspection hours including third party inspections |
| Service Hours | h.y^-1^ | 240 |  |  | Scheduled service hours. Scaled by equipment size. |
| Unscheduled repairs | h.y^-1^ | 208 |  |  | Repair hours. Scaled by equipment size |
|  |  |  |  |  |  |
| **Nutrient Supply** |  |  |  |  |  |
| Unloading and mixing | h.y^-1^ | 4503 |  |  | Nutrients distributed via pond water return systems |
| Unscheduled repairs | h.y^-1^ | 104 |  |  | Repair hours. Scaled by equipment size |
|  |  |  |  |  |  |
| **Operations Labour** |  |  |  |  |  |
| Location Alternative 1 |  | USA Rural |  |  |  |
| All up rate Manager | AUD$.hr^-1^ | 100.50 |  |  | All up rate including productivity factors |
| All up rate Supervisor | AUD$.hr^-1^ | 66.12 |  |  | All up rate including productivity factors |
| All up rate Skilled worker | AUD$.hr^-1^ | 52.89 |  |  | All up rate including productivity factors |
| All up rate Unskilled worker | AUD$.hr^-1^ | 33.06 |  |  | All up rate including productivity factors |
| All up rate Lab Technician | AUD$.hr^-1^ | 58.18 |  |  | All up rate including productivity factors |
| All up rate Operator | AUD$.hr^-1^ | 63.47 |  |  | All up rate including productivity factors |
|  |  |  |  |  |  |
| Location Alternative 2 |  | China Rural |  |  |  |
| All up rate Manager | AUD$.hr^-1^ | 23.80 |  |  | All up rate including productivity factors |
| All up rate Supervisor | AUD$.hr^-1^ | 15.66 |  |  | All up rate including productivity factors |
| All up rate Skilled worker | AUD$.hr^-1^ | 12.53 |  |  | All up rate including productivity factors |
| All up rate Unskilled worker | AUD$.hr^-1^ | 7.83 |  |  | All up rate including productivity factors |
| All up rate Lab Technician | AUD$.hr^-1^ | 13.78 |  |  | All up rate including productivity factors |
| All up rate Operator | AUD$.hr^-1^ | 15.03 |  |  | All up rate including productivity factors |
|  |  |  |  |  |  |
| Location Alternative 3 |  | Australia Rural |  |  |  |
| All up rate Manager | AUD$.hr^-1^ | 67.08 |  |  | All up rate including productivity factors |
| All up rate Supervisor | AUD$.hr^-1^ | 44.13 |  |  | All up rate including productivity factors |
| All up rate Skilled worker | AUD$.hr^-1^ | 35.31 |  |  | All up rate including productivity factors |
| All up rate Unskilled worker | AUD$.hr^-1^ | 22.07 |  |  | All up rate including productivity factors |
| All up rate Lab Technician | AUD$.hr^-1^ | 38.84 |  |  | All up rate including productivity factors |
| All up rate Operator | AUD$.hr^-1^ | 42.37 |  |  | All up rate including productivity factors |

**References**

1 Roles, J. *et al.* Charting a development path to deliver cost competitive solar fuels. *Algae Research* (2018).

2 Bernard, O. & Remond, B. Validation of a simple model accounting for light and temperature effect on microalgal growth. *Bioresource Technol* **123**, 520-527, doi:10.1016/j.biortech.2012.07.022 (2012).

3 Rosso, L., Lobry, J. R. & Flandrois, J. P. An Unexpected Correlation between Cardinal Temperatures of Microbial-Growth Highlighted by a New Model. *J Theor Biol* **162**, 447-463, doi:DOI 10.1006/jtbi.1993.1099 (1993).

4 Bechet, Q., Shilton, A., Fringer, O. B., Munoz, R. & Guieysse, B. Mechanistic Modeling of Broth Temperature in Outdoor Photobioreactors. *Environmental science & technology* **44**, 2197-2203, doi:10.1021/es903214u (2010).

5 Duffie, J. A. & Beckman, W. A. *Solar engineering of thermal processes*. (: Wiley, 1980).

6 Kohler, M. A. & Parmele, L. H. Generalized Estimates of Free-Water Evaporation. *Water Resour Res* **3**, 997-&, doi:DOI 10.1029/WR003i004p00997 (1967).

7 Blaney, H. F. & Morin, K. V. Evaporation and consumptive use of water empirical formulas. *Eos T Am Geophys Un* **23**, 76-83 (1942).

8 BIGELOW, F. H. STUDIES ON THE PHENOMENA OF THE EVAPORATION OF WATER OVER LAKES AND RESERVOIRS %J Monthly Weather Review. **35**, 311-316, doi:10.1175/1520-0493(1907)35<311:Sotpot>2.0.Co;2 (1907).

9 Rohwer, C. *et al.* Evaporation from a Free Water Surface - Discussion. *T Am Soc Civ Eng* **111**, 34-66 (1946).

10 Mansfield, W. W. Influence of Monolayers on the Natural Rate of Evaporation of Water. *Nature* **175**, 247-249, doi:DOI 10.1038/175247a0 (1955).

11 Reca, J., Garcia-Manzano, A. & Martinez, J. Optimal pumping scheduling model considering reservoir evaporation. *Agr Water Manage* **148**, 250-257, doi:10.1016/j.agwat.2014.10.008 (2015).

12 Martinez, J. M. M., Alvarez, V. M., Gonzalez-Real, M. M. & Baille, A. A simulation model for predicting hourly pan evaporation from meteorological data. *J Hydrol* **318**, 250-261, doi:10.1016/j.jhydrol.2005.06.016 (2006).

13 Gallego-Elvira, B., Baille, A., Martin-Gorriz, B. & Martinez-Alvarez, V. Energy balance and evaporation loss of an agricultural reservoir in a semi-arid climate (south-eastern Spain). *Hydrol Process* **24**, 758-766, doi:10.1002/hyp.7520 (2010).

14 Assouline, S. *et al.* Evaporation from three water bodies of different sizes and climates: Measurements and scaling analysis. *Adv Water Resour* **31**, 160-172, doi:10.1016/j.advwatres.2007.07.003 (2008).

15 Assouline, S. *et al.* Evaporation from a shallow water table: Diurnal dynamics of water and heat at the surface of drying sand. *Water Resour Res* **49**, 4022-4034, doi:10.1002/wrcr.20293 (2013).

16 Murphy, T. E. & Berberoğlu, H. Effect of algae pigmentation on photobioreactor productivity and scale-up: A light transfer perspective. *Journal of Quantitative Spectroscopy and Radiative Transfer* **112**, 2826-2834, doi:10.1016/j.jqsrt.2011.08.012 (2011).

17 Lee, S. Crop Oils, Biodiesel, and Algae Fuels. *Green Chem Chem Eng*, 17-51 (2013).

18 Murphy, T. E. & Berberoglu, H. Effect of algae pigmentation on photobioreactor productivity and scale-up: A light transfer perspective. *J Quant Spectrosc Ra* **112**, 2826-2834, doi:10.1016/j.jqsrt.2011.08.012 (2011).

19 Fuentes, M. M. R. *et al.* Outdoor continuous culture of Porphyridium cruentum in a tubular photobioreactor: quantitative analysis of the daily cyclic variation of culture parameters. *Journal of biotechnology* **70**, 271-288 (1999).

20 Pruvost, J., Cornet, J. F., Goetz, V. & Legrand, J. Modeling Dynamic Functioning of Rectangular Photobioreactors in Solar Conditions. *Aiche J* **57**, 1947-1960, doi:10.1002/aic.12389 (2011).

21 Papadakis, I. A., Kotzabasis, K. & Lika, K. A cell-based model for the photoacclimation and CO2-acclimation of the photosynthetic apparatus. *Bba-Bioenergetics* **1708**, 250-261, doi:10.1016/j.bbabio.2005.03.001 (2005).

22 Yarnold, J., Ross, I. L. & Hankamer, B. Photoacclimation and productivity of Chlamydomonas reinhardtii grown in fluctuating light regimes which simulate outdoor algal culture conditions. *Algal Res* **13**, 182-194, doi:10.1016/j.algal.2015.11.001 (2016).

23 Geider, R. J. & Osborne, B. A. Respiration and Microalgal Growth - a Review of the Quantitative Relationship between Dark Respiration and Growth. *New Phytol* **112**, 327-341 (1989).

24 Çengel, Y. A. & Ghajar, A. J. *Heat and mass transfer : fundamentals & applications*. Fifth edition. edn, (McGraw Hill Education, 2015).

25 Lee, E., Pruvost, J., He, X., Munipalli, R. & Pilon, L. Design tool and guidelines for outdoor photobioreactors. *Chem Eng Sci* **106**, 18-29, doi:10.1016/j.ces.2013.11.014 (2014).

26 Quinn, J. C. & Davis, R. The potentials and challenges of algae based biofuels: A review of the techno-economic, life cycle, and resource assessment modeling. *Bioresource Technol* **184**, 444-452, doi:10.1016/j.biortech.2014.10.075 (2015).

27 Canter, C. E., Davis, R., Urgun-Demirtas, M. & Frank, E. D. Infrastructure associated emissions for renewable diesel production from microalgae. *Algal Res* **5**, 195-203, doi:10.1016/j.algal.2014.01.001 (2014).

28 Davis, R. E. *et al.* Integrated Evaluation of Cost, Emissions, and Resource Potential for Algal Biofuels at the National Scale. *Environmental science & technology* **48**, 6035-6042, doi:10.1021/es4055719 (2014).

29 Davis, R. *et al.* Renewable diesel from algal lipids: an integrated baseline for cost, emissions, and resource potential from a harmonized model. (National Renewable Energy Lab.(NREL), Golden, CO (United States), 2012).

30 Page, J. S. *Estimator's Piping Man-Hour Manual*. Fourth Edition edn, Vol. 1 (Gulf Publishing Company, 1958).

31 Moerschner, J. & Lucke, W. Substance chain analysis and LCA in agro industry: Essential contribution of farm machinery manufacturers. *Vdi Bericht* **1503**, 311-316 (1999).

32 Hammond, G., Jones, C., Lowrie, F. & Tse, P. *Embodied carbon : the Inventory of Carbon and Energy (ICE)*. (BSRIA, 2011).

33 Murphy, D. J., Hall, C. A. S., Dale, M. & Cleveland, C. Order from Chaos: A Preliminary Protocol for Determining the EROI of Fuels. *Sustainability-Basel* **3**, 1888-1907, doi:10.3390/su3101888 (2011).

34 Ciceri, N. D., Gutowski, T. G. & Garetti, M. A Tool to Estimate Materials and Manufacturing Energy for a Product. *Proceedings of the 2010 Ieee International Symposium on Sustainable Systems and Technology (Issst)* (2010).

35 Hall, C. A. S., Dale, B. E. & Pimentel, D. Seeking to Understand the Reasons for Different Energy Return on Investment (EROI) Estimates for Biofuels. *Sustainability-Basel* **3**, 2413-2432 (2011).

36 Dixit, M. K., Culp, C. H. & Fernandez-Solis, J. L. Embodied energy of construction materials: integrating human and capital energy into an IO-based hybrid model. *Environmental science & technology* **49**, 1936-1945, doi:10.1021/es503896v (2015).

37 Australian Government. Electricity sector emissions and generation data 2016-2017. (Australian Government,, 2017).

38 Institute for Global Environmental Strategies. IGES GHG Emissions Database. (Institute for Global Environmental Strategies, 2017).

39 Raugei, M. & Leccisi, E. A comprehensive assessment of the energy performance of the full range of electricity generation technologies deployed in the United Kingdom. *Energ Policy* **90**, 46-59, doi:10.1016/j.enpol.2015.12.011 (2016).

40 Rogers, J. N. *et al.* A critical analysis of paddlewheel-driven raceway ponds for algal biofuel production at commercial scales. *Algal Res* **4**, 76-88, doi:10.1016/j.algal.2013.11.007 (2014).

41 Lee, A. K., Lewis, D. M. & Ashman, P. J. Harvesting of marine microalgae by electroflocculation: The energetics, plant design, and economics. *Appl Energ* **108**, 45-53, doi:10.1016/j.apenergy.2013.03.003 (2013).

42 Elliott, D. C., Biller, P., Ross, A. B., Schmidt, A. J. & Jones, S. B. Hydrothermal liquefaction of biomass: Developments from batch to continuous process. *Bioresource Technol* **178**, 147-156, doi:10.1016/j.biortech.2014.09.132 (2015).

43 Fushimi, C., Kakimura, M., Tomita, R., Umeda, A. & Tanaka, T. Enhancement of nutrient recovery from microalgae in hydrothermal liquefaction using activated carbon. *Fuel Process Technol* **148**, 282-288, doi:10.1016/j.fuproc.2016.03.006 (2016).

44 Lee, A., Lewis, D., Kalaitzidis, T. & Ashman, P. Technical issues in the large-scale hydrothermal liquefaction of microalgal biomass to biocrude. *Current opinion in biotechnology* **38**, 85-89, doi:10.1016/j.copbio.2016.01.004 (2016).

45 Liou, C. P. Limitations and proper use of the Hazen-Williams equation. *Journal of Hydraulic Engineering* **124**, 951-954 (1998).

46 Johnson, M. C., Palou-Rivera, I. & Frank, E. D. Energy consumption during the manufacture of nutrients for algae cultivation. *Algal Res* **2**, 426-436, doi:10.1016/j.algal.2013.08.003 (2013).

47 Radzun, K. A. *et al.* Automated nutrient screening system enables high-throughput optimisation of microalgae production conditions. *Biotechnology for biofuels* **8**, doi:UNSP 6510.1186/s13068-015-0238-7 (2015).

48 Geider, R. J. & La Roche, J. Redfield revisited: variability of C : N : P in marine microalgae and its biochemical basis. *Eur J Phycol* **37**, 1-17 (2002).

49 Davis, R., Aden, A. & Pienkos, P. T. Techno-economic analysis of autotrophic microalgae for fuel production. *Appl Energ* **88**, 3524-3531, doi:10.1016/j.apenergy.2011.04.018 (2011).

50 Luckow, P. S., E. 2015 Carbon Dioxide Price Forecast. (2015).

51 De Mes, T., Stams, A., Reith, J. & Zeeman, G. Methane production by anaerobic digestion of wastewater and solid wastes. *Bio-methane & Bio-hydrogen*, 58-102 (2003).
